# Short Enantioselective Total Synthesis of Tatanan A and 3‐*epi*‐Tatanan A Using Assembly‐Line Synthesis

**DOI:** 10.1002/anie.201609598

**Published:** 2016-11-16

**Authors:** Adam Noble, Stefan Roesner, Varinder K. Aggarwal

**Affiliations:** ^1^ School of ChemistryUniversity of Bristol, Cantock's CloseBristolBS8 1TSUK

**Keywords:** alkynylation, lithiation–borylation, Matteson homologation, tatanan A, total synthesis

## Abstract

Short and highly stereoselective total syntheses of the sesquilignan natural product tatanan A and its C3 epimer are described. An assembly‐line synthesis approach, using iterative lithiation–borylation reactions, was applied to install the three contiguous stereocenters with high enantio‐ and diastereoselectivity. One of the stereocenters was installed using a configurationally labile lithiated primary benzyl benzoate, resulting in high levels of substrate‐controlled (undesired) diastereoselectivity. However, reversal of selectivity was achieved by using a novel diastereoselective Matteson homologation. Stereospecific alkynylation of a hindered secondary benzylic boronic ester enabled completion of the synthesis in a total of eight steps.

Iterative strategies are highly attractive for the synthesis of complex molecules,^1^ particularly when minimal or no functional‐group manipulations between chain‐extension steps are required.^2^, ^3^ Iterative aldol reactions provide one such strategy,^3^ but if the target molecule is devoid of appropriate functional‐group handles, alternative methodologies are required. We recently reported an iterative strategy for the homologation of boronic esters that notably does not require any functional‐group manipulations between chain‐extension steps.^4^, ^5^ The process involves the repeated addition of chiral lithiated carbamates or triisopropylbenzoate (TIB) esters and leads to carbon chains bearing multiple contiguous methyl‐substituted stereogenic centers (Figure 1 A). This approach enabled the generation of extended chains of vicinal stereocenters (up to 10) with complete control over the relative and absolute stereochemistry and applications to complex natural products have also been reported.^6^

**Figure 1:**
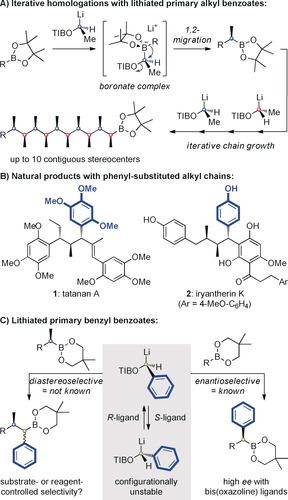
A) Iterative homologation of boronic esters. B) Natural products with alkyl‐ and aryl‐substituted carbon chains. C) Use of primary benzyl benzoates in homologation of boronic esters.

The power of iterative homologation of boronic esters lies in its versatility since other alkyl groups can be easily incorporated simply by varying the groups on the lithiated benzoate reagent. In extending the reach of this method, we sought to introduce aryl substituents as this would enable access to an even broader array of targets, for example tatanan A (**1**) and iryantherin K (**2**) (Figure 1 B).^7^, ^8^ We targeted the sesquilignan tatanan A (**1**) as this structurally unique molecule had been reported to display potent glucokinase‐activating properties, thereby having implications for the development of antihyperglycemic drugs, although its bioactivity has since been questioned by Zakarian, who also reported its first synthesis.^9^ The synthesis of such a molecule would require homologation with a mixture of alkyl‐ and aryl‐substituted lithiated benzoates. Whilst alkyl‐substituted lithiated benzoates were known to be effective in assembly‐line synthesis, little was known about the aryl‐substituted lithiated benzoates.^10^, ^11^ Such species present additional challenges in that, unlike the alkyl‐substituted lithiated benzoates, they are configurationally unstable, although they can be generated in high enantioselectivity upon deprotonation in the presence of chiral bis(oxazoline) ligands.^12^ Furthermore, they have been employed in homologations of neopentyl glycol boronic esters (which give higher selectivity than pinacol boronic esters), but how they would perform with chiral boronic esters was not known (Figure 1 C).^13^ Herein, we describe our investigations into the use of lithiated primary benzyl benzoates in diastereoselective homologations with chiral boronic esters and its application to the synthesis of tatanan A and 3‐*epi*‐tatanan A.

We envisioned that tatanan A (**1**) could be prepared through an olefination of benzylic boronic ester **3** with β‐styrenyl iodide **4** by using either a Pd‐catalyzed Suzuki cross‐coupling or a Zweifel‐type reaction (Scheme 1).^14^, ^15^ Neither reaction had extensive precedent so as a contingency plan, we considered employing a reaction sequence consisting of stereospecific alkynylation of **3**,^16^ forming terminal alkyne **5**, followed by *syn* carboalumination/iodination and Suzuki cross‐coupling.^17^ Importantly, boronic ester **3** could be generated using our assembly‐line synthesis by sequential reaction of aryl boronic ester **6** with lithiated building blocks **7**, **8** and **9**.

**Scheme 1:**
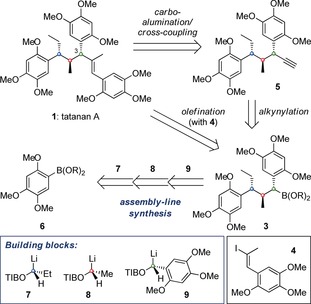
Retrosynthetic analysis of tatanan A.

The synthesis of **1** began with the preparation of secondary neopentyl glycol boronic ester **10**, which was required in order to investigate the key lithiation–borylation with building block **9** (Scheme 2).13a Iodination of 1,2,4‐trimethoxybenzene (**11**) afforded aryl iodide **12**, which was converted to aryl boronic ester **13** in excellent yield by halogen‐lithium exchange, trapping with triisopropyl borate, and esterification with neopentyl glycol. Iterative homologations of **13** with building blocks **7**, giving benzylic boronic ester **14**, and **8** yielded **10** in 54 % yield with excellent diastereo‐ and enantioselectivity.

**Scheme 2:**
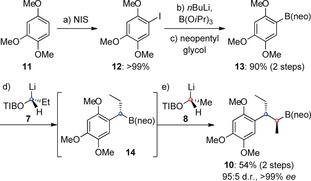
Synthesis of secondary alkyl boronic ester **10**. NIS=*N*‐iodosuccinimide; neo=neopentyl glycolato; TIB=2,4,6‐triisopropylbenzoyl.

With boronic ester **10** in hand, our attention turned to the key lithiation–borylation reaction (Table 1). Deprotonation of benzyl benzoate **16** with *s*BuLi in the presence of chiral bis(oxazoline) (*S*,*S*)‐L* followed by addition of boronic ester **10** gave benzylic boronic ester **15** in high yield and with excellent diastereoselectivity after transesterification with pinacol (entry 1).^18^ Surprisingly, switching to the enantiomeric ligand (*R*,*R*)‐L* led to the same major diastereoisomer, albeit with slightly lower selectivity (entry 2). Furthermore, the use of the achiral ligand TMEDA also gave very high selectivity again for the same major isomer (entry 3). Unfortunately, the major diastereoisomer, **15 b**, was determined to have the undesired *S*‐configuration at the newly formed stereocenter.^19^ These results demonstrate that boronic ester **10** shows a very high level of substrate control, which dominates the thermodynamically preferred configuration of the lithiated benzoate. It also shows that the diastereoselectivity is affected by the nature of the ligand ligated to lithium. Switching from the neopentyl glycol boronic ester **10** to the corresponding pinacol boronic ester **17** resulted in lower yield and almost complete loss of diastereocontrol (entry 4).

**Table 1:** Ligand effects in the reaction of **10** with lithiated benzoate **9**. 
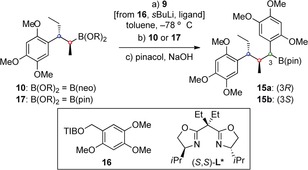

Entry^[a]^	B(OR)_2_	Ligand	Conv. [%]^[b]^	Yield [%]^[c]^	**15 a**/**15 b** ^[d]^
1	B(neo)	(*S*,*S*)‐L*	95	72	6:94
2	B(neo)	(*R*,*R*)‐L*	72	52	12:88
3	B(neo)	TMEDA	77	61	2:98
4	B(pin)	TMEDA	32	29	53:47

[a] See the Supporting Information for reaction conditions. [b] Conversion of **10**/**17** into **15** determined by ^1^H NMR. [c] Yield after purification. [d] Determined by ^1^H NMR. TMEDA=*N*,*N*,*N*′,*N*′‐tetramethylethylenediamine; pin=pinacolato.

We initially attempted to use the “undesired” diastereoisomer **15 b** in an invertive Suzuki cross‐coupling, recently described by Biscoe, but this was unsuccessful.^20^, ^21^ We then considered the possibility of exploiting the high level of substrate control to selectively generate the other diastereomeric boronic ester **15 a**. We believe that the high diastereoselectivity in the reaction of **9** with **10** arises from a kinetically controlled stereoselective boronate complex formation in which (*S*)‐**9** reacts at a faster rate than (*R*)‐**9** leading to the selective formation of boronate complex (*S*)‐**18** (Scheme 3 A). Subsequent 1,2‐migration then provides benzylic boronic ester **19 b**, the neopentyl glycol analogue of **15 b**. In an attempt to reverse this selectivity, we proposed to react lithiated chloromethyl benzoate **20** in place of **9** (Scheme 3 B). Here, a diastereoselective Matteson homologation with boronic ester **10** (or **17**) would lead to boronate complex (*S*)‐**21**.^22^ Selective expulsion of chloride in the 1,2‐migration would give α‐oxy boronic ester **22**, which could then be reacted with an aryl metal species to generate benzylic boronic ester **3** possessing the desired stereochemistry. The use of lithium species, such as **20**, containing two different leaving groups has not been previously reported, nor has this strategy for reversing diastereoselectivity.

**Scheme 3:**
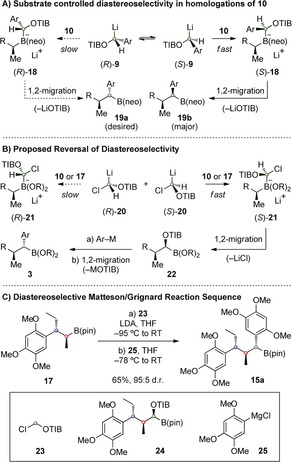
Alternative strategy for the third homologation. A) Plausible mechanism for high diastereoselectivity in homologations of **10** with **9**. B) Proposed diastereoselective Matteson homologation. C) Optimized conditions for reversal of diastereoselectivity. Ar=2,4,5‐trimethoxyphenyl; LDA=lithium diisopropylamide.

After some optimization, we found that addition of LDA to a mixture of pinacol boronic ester **17** and chloromethyl 2,4,6‐triisopropylbenzoate (**23**) (in situ lithiation) gave α‐oxy boronic ester **24** in good yield and 77:23 d.r. (Scheme 3 C).^20^ The use of non‐symmetrical lithiated chloromethyl ester **23** proved essential as the use of symmetrical (dichloromethyl)lithium resulted in an unselective reaction. While the reaction of neopentyl glycol boronic ester **10** with lithiated **23** gave higher diastereoselectivity (90:10 d.r.), a significantly lower yield was obtained compared to pinacol derivative **17**. Subsequent treatment of **24** with aryl Grignard **25** provided the desired diastereoisomer **15 a** in 65 % yield and higher d.r. (95:5), presumably as a result of a degree of kinetic resolution.^23^

The complete synthesis of the natural isomer of tatanan A and its C3 epimer are shown in Scheme 4. Aryl pinacol boronic ester **26** was prepared in high yield by iodination and borylation of 1,2,4‐trimethoxybenzene (**11**). Our assembly‐line synthesis then began with sequential reaction of **26** with building blocks **7** and **8** in a one‐pot procedure to provide **17** in 74 % yield, >99 % *ee* and 94:6 d.r. Subsequent diastereoselective Matteson reaction completed the assembly line to yield **15 a** in good yield and high diastereoselectivity. Completion of the synthesis in a single step from **15 a** proved challenging and all attempts using vinyl iodide **4** under either Suzuki^14^ or Zweifel^15^ conditions failed to give the desired product.^20^ We therefore turned to incorporation of the vinyl moiety via alkyne **5** using the stereospecific alkynylation methodology recently reported by our group.^16^ This two‐step protocol proceeds via a Zweifel olefination with lithiated vinyl carbamate **27** followed by base‐mediated elimination of the resulting vinyl carbamate to furnish **5** in excellent yield and with complete diastereospecificity. Zirconium‐catalyzed carboalumination with trimethylaluminum and subsequent trapping of the intermediate vinyl aluminum species with iodine generated vinyl iodide **28** in moderate yield.^17^ This reaction was complicated by slow reaction rates and competitive protonation of the intermediate aluminum species. Finally, Suzuki cross‐coupling with aryl boronic acid **29** gave tatanan A (**1**) in 93 % yield, as a single diastereomer and in >99 % *ee*. The spectroscopic data were found to be identical to those reported previously.^7^, ^9^ The same sequence was also applied to boronic ester **15 b** giving 3‐*epi*‐tatanan A in 54 % yield over four steps.

**Scheme 4:**
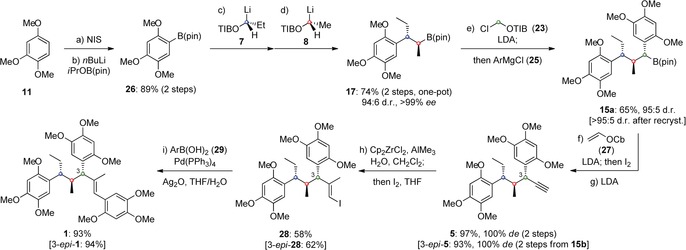
Total synthesis of tatanan A and 3‐*epi*‐tatanan A. Ar=2,4,5‐trimethoxyphenyl. Cb=*N*,*N*‐diisopropylcarbamoyl.

In summary, we have developed a highly enantio‐ and diastereoselective eight‐step total synthesis of tatanan A and its C3 epimer using an assembly‐line synthesis approach. More importantly, for substrates which show high levels of substrate control, we have identified conditions under which either stereoisomer of benzylic boronic esters can be incorporated into an assembly‐line synthesis. For substrates which show little substrate control, the bisoxazoline ligands can be used to control the configuration of the benzylic center. This new strategy further expands the range of targets that are now accessible with this methodology.

## Supporting information

As a service to our authors and readers, this journal provides supporting information supplied by the authors. Such materials are peer reviewed and may be re‐organized for online delivery, but are not copy‐edited or typeset. Technical support issues arising from supporting information (other than missing files) should be addressed to the authors.

Supplementary
