# Supplementary material for: Short Enantioselective Total Synthesis of Tatanan A and 3‐epi‐Tatanan A Using Assembly‐Line Synthesis
Source: Angew Chem Int Ed Engl. 2016 Nov 16;55(51):15920–4. doi: 10.1002/anie.201609598 (PMC5215435; doi:10.1002/anie.201609598)

Angewandte Chemie
Eine Zeitschrift der Gesellschaft Deutscher Chemiker

Supporting Information

Short Enantioselective Total Synthesis of Tatanan A and 3-*epi*-Tatanan A Using Assembly-Line Synthesis

*Adam Noble, Stefan Roesner, and Varinder K. Aggarwal**

anie_201609598_sm_miscellaneous_information.pdf

Supplementary Information for

Short, Enantioselective Total Synthesis of Tatatan A, and 3-*epi*-Tatanan A, Using Assembly-Line Synthesis

Adam Noble, Stefan Roesner, and Varinder K. Aggarwal*

School of Chemistry, University of Bristol

Cantock's Close, Bristol, BS8 1TS (UK)

Contents

1. General Information 2
2. Materials and Reagents 3
3. Synthesis of 2,4,5-Trimethoxyphenyl Boronic Acid Derivatives 4
4. Synthesis of Benzoate Esters and Carbamates 7
5. Synthesis of Neopentyl Glycol Boronic Ester 10 12
6. Ligand Studies in Lithiation-Borylations with Primary Benzylic TIB-ester 16 14
7. End Game Studies: Stereospecific Olefinations Using Suzuki and Zweifel Reactions 19
8. Synthesis of 3-*epi*-Tatanan A 27
9. Synthesis of Racemic Pinacol Boronic Ester 17 30
10. Optimization of the Diastereoselective Matteson Homologation 32
11. Synthesis of Tatanan A 35
12. Comparison of NMR Spectra of Natural and Synthetic Tatanan A 41
13. ^{1}H and ^{13}C NMR spectra 43

1. General Information

All air and water-sensitive reactions were carried out in flame-dried glassware under a nitrogen atmosphere using standard Schlenk manifold technique. Cryogenic temperatures were achieved used the following cold baths: acetone/CO_2 (–78 °C); MeOH/N_2 (–95 °C).

Analytical thin-layer chromatography (TLC) was performed using aluminium backed plates pre-coated with Merck Silica Gel 60 F254. Compounds were visualized under UV light or by staining with aqueous basic potassium permanganate or an ethanolic solution of phosphomolybdic acid. Flash column chromatography (FCC) was carried out using silica gel LC60A-40 (63 µm).

Chiral HPLC was performed on a HP Agilent 1100 with a Chiralpak IB column (4.6 mm × 250 mm, 5 µm) fitted with a guard (4 mm × 10 mm) and monitored by DAD (Diode Array Detector). Chiral supercritical fluid chromatography (SFC) was performed on a Thar SFC investigator using a Daicel Chiralpak IB column (4.6 mm × 250 mm, 5 µm).

^{1}H, ^{13}C and ^{11}B NMR spectra were acquired at various field strengths, as indicated, using Jeol ECS 300 MHz, Jeol ECS 400 MHz, Varian VNMR 400 MHz, Varian VNMR 500 MHz, and Bruker Cryo 500 MHz Fourier transform spectrometers. Chemical shifts (δ) are given in parts per million (ppm) and referenced to $CDCl_3$ (7.27 ppm), methanol-d_4 (3.31 ppm) or DMSO-d_6 (2.50 ppm). Coupling constants (J) are given in Hertz (Hz) and refer to apparent multiplicities (s = singlet, br s = broad singlet, d = doublet, t = triplet, q = quartet, quin = quintet, sex = sextet, hep = heptet, m = multiplet, dd = doublet of doublets, etc.). The ^{1}H NMR spectra are reported as follows: chemical shift (multiplicity, coupling constants, number of protons).

High resolution mass spectra (HRMS) were recorded on a VG Analytical Autospec by Electron Ionization (EI) or Chemical Ionization (CI) or on a Bruker Daltonics Apex IV by Electrospray Ionization (ESI).

Infra-red (IR) spectra were recorded on a Perkin Elmer Spectrum One FT-IR as a thin film. Selected absorption maxima (ν_{max}) are reported in wavenumbers (cm^{-1}).

Melting points were recorded in degrees Celsius (°C), using a Kofler hot-stage microscope apparatus and are reported uncorrected.

Microwave reactions were carried out in a Biotage Initiator EXP EU microwave synthesizer

Optical rotation ($[\alpha]_D^T$) was measured on a Bellingham and Stanley Ltd. ADP220 polarimeter and is quoted in (° ml)(g dm)$^{-1}$.

2. Materials and Reagents

All reagents were used as received unless otherwise stated. Bulk solutions were evaporated under reduced pressure using a Büchi rotary evaporator. All anhydrous solvents were commercially supplied or dried by passing through a modified Grubbs system of alumina columns, manufactured by Anhydrous Engineering. Petroleum ether refers to the fraction collected between 40 ºC and 60 °C. *n*BuLi (1.6 M in hexanes) and *s*BuLi (1.3 M in 92:8 cyclohexane/hexane) were purchased from Acros. The molarity of organolithium solutions was regularly determined by titration using *N*-benzyl benzamide as an indicator.[1] TMEDA was dried over CaH_2 before distillation and stored in a Young's tube under N_2. Diisopropylamine and 2,2,6,6-tetramethylpiperidine were dried over NaOH before distillation and stored in a Young's tube under N_2. (+)-Sparteine was obtained as the free base (BOC sciences), distilled over CaH_2 or NaOH and stored in a Young's tube under N_2. The free base of sparteine readily absorbs atmospheric carbon dioxide (CO_2) and should be stored in a Young's tube under inert atmosphere at −20 °C. Lithium diisopropylamide (LDA) and lithium 2,2,6,6-tetramethylpiperide (LiTMP) solutions were freshly prepared from the corresponding amine and *n*-BuLi (1.6 M in hexanes) immediately before use.

The following compounds were prepared according to literature procedure and all spectroscopic data matched those previously reported: Ethyl 2,4,6-triisopropylbenzoate (EtOTIB, **S1**),[2] (*R*)-1-(trimethylstannyl)ethyl 2,4,6-triisopropylbenzoate (**S2**),[2] vinyl diisopropylcarbamate (**27**),[3] 2-ethyl-4,4,5,5-tetramethyl-1,3,2-dioxaborolane [EtB(pin)],[4] 2-isopropyl-5,5-dimethyl-1,3,2-dioxaborinane [*i*PrB(neo)],[4] (4*S*,4′*S*)-2,2′-(3-pentylidene)bis(4-*tert*-butyloxazoline) [(*S*,*S*)-*t*Bu-BOX],[5] (4*S*,4′*S*)-2,2′-(3-pentylidene)bis(4-isopropyloxazoline) [(*S*,*S*)-*i*Pr-BOX, **L***],[5] Buchwald precatalyst (*t*Bu_3P-Pd-G3),[6] and 1,1-bis[(pinacolato)boryl]ethane (**S3**).[7]

[1] A. F. Burchat, J. M. Chong, N. Nielsen, *J. Organomet. Chem.* **1997**, *542*, 281 – 283.
[2] M. Burns, S. Essafi, J. R. Bame, S. P. Bull, M. P. Webster, S. Balieu, J. W. Dale, C. P. Butts, J. N. Harvey, V. K. Aggarwal, *Nature* **2014**, *513*, 183 − 188.
[3] N. J. Webb, S. P. Marsden, S. A. Raw, *Org. Lett.* **2014**, *16*, 4718 – 4721.
[4] A. P. Pulis, D. J. Blair, E. Torres, V. K. Aggarwal, *J. Am. Chem. Soc.* **2013**, *135*, 16054 – 16057.
[5] M. Li, A. Hawkins, D. M. Barber, P. Bultinck, W. Herrebout, D. J. Dixon, *Chem. Commun.* **2013**, *49*, 5265 – 5267.
[6] N. C. Bruno, M. T. Tudge, S. L. Buchwald, *Chem. Sci.* **2013**, *4*, 916 – 920.
[7] Z.-Q. Zhang, C.-T. Yang, L.-J. Liang, B. Xiao, X. Lu,; J.-H. Liu, Y.-Y. Sun, T. B. Marder, Y. Fu, *Org. Lett.*, **2014**, *16*, 6342 – 6345.

3. Synthesis of 2,4,5-Trimethoxyphenyl Boronic Acid Derivatives

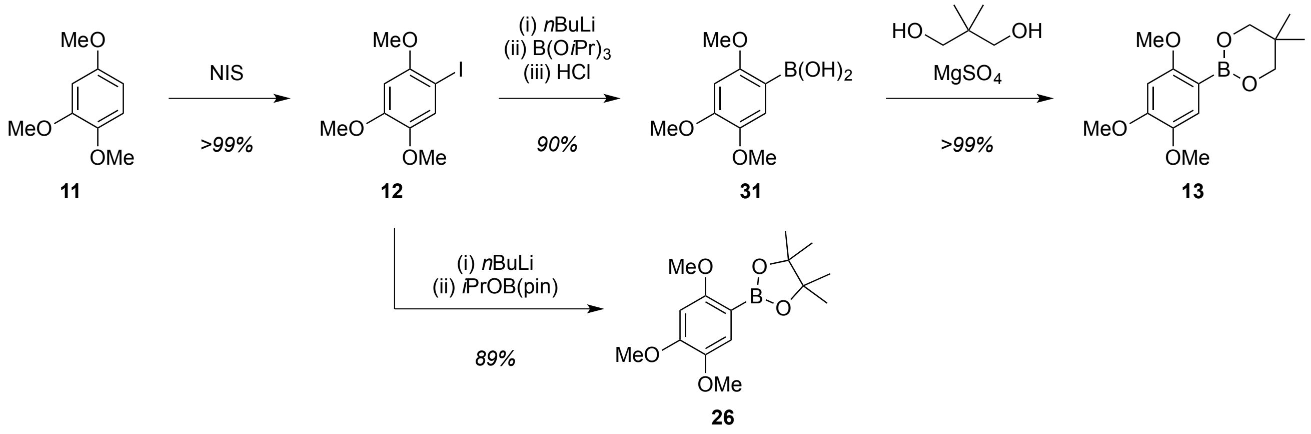

1-Iodo-2,4,5-trimethoxybenzene (12)

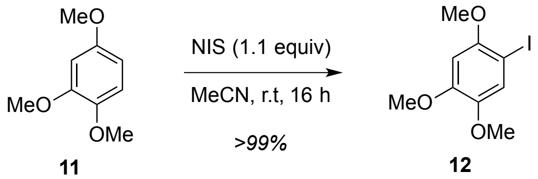

Prepared following a modified literature procedure.[8] *N*-Iodosuccinimide (12.4 g, 55.0 mmol, 1.1 equiv) was added to a solution of 1,2,4-trimethoxybenzene (**11**) (8.41 g, 50.0mmol, 1.0 equiv) in anhydrous MeCN (150 mL) and stirred 16 h at ambient temperature. The solvent was removed *in vacuo* and the residue was dissolved in Et_2O (100 mL). The ethereal solution was washed with saturated aqueous $Na_2S_2O_3$ solution (100 mL) and water (100 mL), dried over $MgSO_4$, filtered and concentrated *in vacuo* to give the title compound (14.7 g, 49.9 mmol, >99%) as an off-white solid, which required no further purification.

^{1}H NMR (400 MHz, $CDCl_3$) δ_H 7.22 (s, 1H), 6.52 (s, 1H), 3.90 (s, 3H), 3.86 (s, 3H), 3.84 (s, 3H) ppm.

^{13}C NMR (126 MHz, $CDCl_3$) δ_C 153.0, 150.2, 144.2, 121.9, 97.8, 73.0, 57.3, 56.7, 56.1 ppm.

All spectroscopic data are consistent with those previously reported in the literature.[9]

(2,4,5-Trimethoxyphenyl)boronic acid (29)

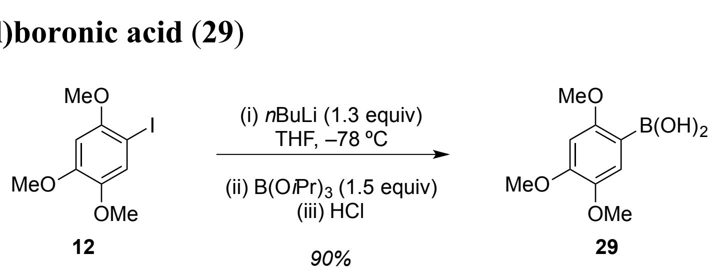

n-Butyllithium (8.13 mL, 1.60 M solution in hexanes, 13.0 mmol, 1.3 equiv) was added dropwise to a solution of 1-iodo-2,4,5-trimethoxybenzene (**12**) (2.94 g, 10.0 mmol, 1.0 equiv) in anhydrous THF (25 mL) at −78 °C. After stirring for 30 min triisopropyl borate (2.05 mL, 15.0 mmol, 1.5 equiv) was added slowly and the mixture was stirred for 4 h at −78 °C before allowing to warm to room temperature and stirring for a

[8] M. C. Carreño, J. L. Ruano, G. Sanz, M. A. Toledo, A. Urbano, *Tetrahedron Lett.* **1996**, *37*, 4081 − 4084.
[9] S. Huenig, R. Bau, M. Kemmer, H. Meixner, T. Metzenthin, K. Peters, K. Singer, J. Gulbis, *Eur. J. Org. Chem.* **1998**, 335 − 348.

further 16 h. Saturated aqueous NH_4Cl solution (20 mL) was added and the mixture was acidified with 3 M HCl to pH 5. The organic solvent was removed under reduced pressure. The crude solids were collected by vacuum filtration, washed with water and dried *in vacuo* to afford the title compound (1.90 g, 8.98 mmol, 90%) as a pale green solid, which required no further purification.

^{1}H NMR (300 MHz, $CDCl_3$) δ_H 7.32 (s, 1H), 6.53 (s, 1H), 5.82 (s, 2H), 3.94 (s, 3H), 3.92 (s, 3H), 3.89 (s, 3H) ppm.

^{11}B NMR (96 MHz, $CDCl_3$) δ_B 28.1 (br s) ppm.

All spectroscopic data are consistent with those previously reported in the literature.[10]

5,5-Dimethyl-2-(2,4,5-trimethoxyphenyl)-1,3,2-dioxaborinane (13)

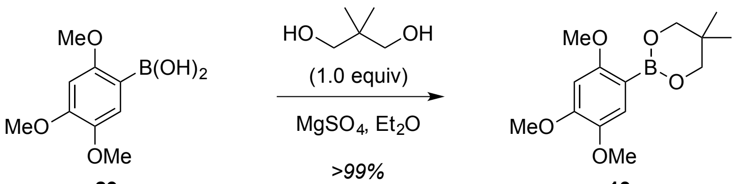

29 **13**

Boronic acid **29** (1.74 g, 8.20 mmol, 1.0 equiv) was dissolved in Et_2O (25 mL), neopentyl glycol (855 mg, 8.20 mmol, 1.0 equiv) was added and the mixture was stirred at room temperature for 16 h. Flame-dried $MgSO_4$ (2.96 g, 24.6 mmol, 3.0 equiv) was added and the reaction mixture was stirred for an additional 2 h at room temperature. The ethereal solution was filtered through a plug of anhydrous $MgSO_4$ and the solids were washed with Et_2O. The filtrate was concentrated *in vacuo* and dried under high vacuum to the title compound (2.30 g, 8.20 mmol, >99%) as a pale green solid, which required no further purification.

Mpt: 61–63 °C (Et_2O).

^{1}H NMR (400 MHz, $CDCl_3$) δ_H 7.21 (s, 1H), 6.49 (s, 1H), 3.90 (s, 3H), 3.87 (s, 3H), 3.82 (s, 3H), 3.78 (s, 4H), 1.03 (s, 6H) ppm.

^{13}C NMR (101 MHz, $CDCl_3$) δ_C 159.8, 151.9, 143.0, 118.6, 97.7, 72.6, 57.4, 56.5, 56.0, 31.9, 22.1 ppm.

^{11}B NMR (96 MHz, $CDCl_3$) δ_B 25.8 (br s) ppm.

IR (ν_{max}/cm^{-1}, neat): 2961, 1603, 1264, 1199, 1160, 1030.

HRMS (CI^+) calcd. for $C_{19}H_{31}O_5BNa$ $[M+Na]^+$ 373.2160, found 373.2164.

[10] a) M. J. Burns, I. J. S. Fairlamb, A. R. Kapdi, P. Sehnal, R. J. K. Taylor, *Org. Lett.* **2007**, *9*, 5397 – 5400; b) G. Wang, F. Wang, D. Cao, Y. Liu, R. Zhang, H. Ye, X. Li, L. He, Z. Yang, L. Ma, A. Peng, M. Xiang, Y. Wei, L. Chen, *Bioorg. Med. Chem. Lett.* **2014**, *24*, 3158 – 3163.

4,4,5,5-Tetramethyl-2-(2,4,5-trimethoxyphenyl)-1,3,2-dioxaborolane (26)

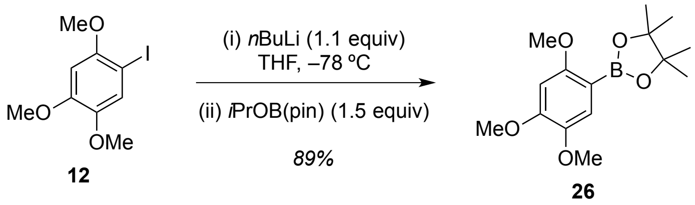

n-Butyllithium (6.74 mL, 1.60 M solution in hexanes, 10.8 mmol, 1.1 equiv) was added dropwise to a solution of 1-iodo-2,4,5-trimethoxybenzene (**12**) (2.88 g, 9.80 mmol, 1.0 equiv) in anhydrous THF (20 mL) at −78 °C. After stirring for 30 min 2-isopropoxy-4,4,5,5-tetramethyl-1,3,2-dioxaborolane (2.74 g, 14.7 mmol, 1.5 equiv) was added slowly and the mixture was stirred for 1 h at −78 °C. Water (10 mL) was added carefully and the mixture was allowed to warm to room temperature. The reaction mixture was extracted with CH_2Cl_2 (3 × 15 mL). The combined organic phases were dried over $MgSO_4$, filtered, and concentrated *in vacuo* to afford the title compound (2.57 g, 8.73 mmol, 89%) as an off-white solid, which required no further purification. Recrystallization from pentane/EtOAc gave a white solid.

Mpt: 106–107 °C (pentane/EtOAc).

^{1}H NMR (400 MHz, $CDCl_3$) δ_H 7.20 (s, 1H), 6.50 (s, 1H), 3.91 (s, 3H), 3.89 (s, 3H), 3.83 (s, 3H), 1.35 (s, 12H) ppm.

^{13}C NMR (101 MHz, $CDCl_3$) δ_C 160.2, 152.4, 142.8, 119.0, 108.4 (br), 97.5, 83.1, 57.3, 56.4, 55.7, 24.8 ppm.

^{11}B NMR (96 MHz, $CDCl_3$) δ_B 28.9 (br s) ppm.

IR (ν_{max}/cm^{-1}, neat): 2965, 1605, 1453, 1344, 1206, 1143, 1032.

HRMS (CI^+) calcd. for $C_{15}H_{24}O_5B$ $[M+H]^+$ 295.1717, found 295.1718.

4. Synthesis of Benzoate Esters and Carbamates

Propyl 2,4,6-triisopropylbenzoate (S4)

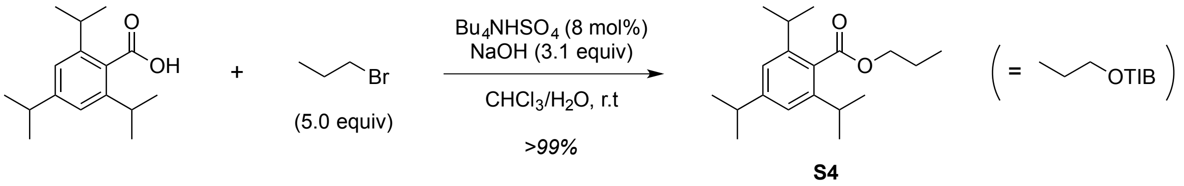

Prepared following a modified literature procedure.[2] A mixture of 2,4,6-triisopropylbenzoic acid (12.4 g, 50.0 mmol, 1.00 equiv), 1-bromopropane (22.7 mL, 250 mmol, 5.00 equiv), tetrabutylammonium bisulfate (1.36 g, 4.00 mmol, 8.00 mol%), sodium hydroxide (6.20 g, 155 mmol, 3.10 equiv), $CHCl_3$ (250 mL) and H_2O (100 mL) were stirred vigorously for 67 h. The organic phase was separated and the aqueous extracted with CH_2Cl_2 (3 × 80 mL). The combined organic phases were washed with brine (300 mL), dried ($MgSO_4$), filtered, and concentrated *in vacuo*. To the residue was added pentane (100 mL), the insoluble salts were removed by filtration and washed with pentane (50 mL). The filtrate was concentrated *in vacuo* to give the title compound (14.5 g, 49.9 mmol, >99%) as a colourless oil that required no further purification.

^{1}H NMR (400 MHz, $CDCl_3$): δ_H 7.02 (s, 2H), 4.28 (t, *J* = 6.7 Hz, 2H), 2.90 (hept, *J* = 6.7, 1H), 2.87 (hept, *J* = 6.7, 2H), 1.77 (sex, *J* = 7.2 Hz, 2H), 1.27 (d, *J* = 6.8 Hz, 12H), 1.26 (d, *J* = 7.0 Hz, 6H), 1.01 (t, *J* = 7.4 Hz, 3H) ppm.

^{13}C NMR (101 MHz, $CDCl_3$): δ_C 171.2, 150.2, 144.9, 130.9, 121.0, 66.8, 34.6, 31.6, 24.3, 24.1, 22.2, 10.7 ppm.

All spectroscopic data are consistent with those previously reported in the literature.[11]

(*R*)-1-(Trimethylstannyl)propyl 2,4,6-triisopropylbenzoate (S5)

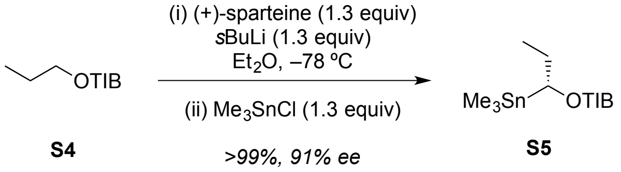

Prepared using a modified literature procedure.[2] An oven-dried Schlenk tube was evacuated and refilled with nitrogen (× 3) before the addition of propyl 2,4,6-triisopropylbenzoate (**S4**) (2.11 g, 7.26 mmol, 1.00 equiv) and (+)-sparteine (2.17 mL, 9.44 mmol, 1.30 equiv) and anhydrous Et_2O (36 mL, 0.20 M). The solution was cooled to –78 °C before the addition of *s*BuLi (1.3 M in hexane/cyclohexane, 7.26 mL, 9.44 mmol, 1.30 equiv) dropwise over 10 min (colour change: colourless to dark brown). The reaction mixture was stirred at –78 °C for 4 h. Me_3SnCl (1.0 M in hexanes, 9.44 mL, 9.44 mmol, 1.30 equiv) was added dropwise to the reaction mixture over 5 min (colour change: dark brown to yellow). The reaction was stirred at –78 °C for 60 min before being warmed to room temperature and stirred for a further 60 min. The reaction was quenched by the addition of 5% aqueous H_3PO_4 (40 mL) and stirred for 20 min before separation of the layers. The organic layer was washed with 5% aqueous H_3PO_4 (3 × 20 mL). The combined aqueous layers

[11] P. Beak, L. G. Carter, *J. Org. Chem.* **1981**, *46*, 2363 – 2373.

were then extracted with Et_2O (3 × 20 mL) and the combined organic extracts were washed with brine (40 mL), dried ($MgSO_4$), filtered and concentrated *in vacuo* to give the title compound as a colourless oil (3.29 g, 7.26 mmol, >99%, 91% ee), which required no further purification. The combined acidic aqueous washings were retained to recover (+)-sparteine.[2]

R_f: 0.12 (100% pet. ether).

^{1}H NMR (400 MHz, $CDCl_3$): δ_H 7.01 (s, 2H), 4.96 (t, J = 6.7 Hz and dt, J = 9.6, 7.1 Hz, 1H), 2.89 (hept, J = 6.9 Hz, 1H), 2.86 (hept, J = 6.8 Hz, 2H), 1.98 (quin, J = 7.2 Hz, and dquin, J = 48.3, 7.1 Hz, 2H), 1.25 (d, J = 6.9 Hz, 12H), 1.25 (d, J = 6.8 Hz, 6H), 1.02 (t, J = 7.3 Hz, 3H), 0.21 (s and d, J = 53.9 Hz and d, J = 51.6 Hz, 9H) ppm.

^{13}C NMR (101 MHz, $CDCl_3$): δ_C 171.6, 150.0, 145.0, 131.0, 121.0, 74.2, 34.5, 31.6, 27.0, 24.6, 24.3, 24.1, 12.4, –8.9 ppm.

IR (ν_{max}/cm^{-1}, neat): 2962–2871, 1705, 1461, 1283, 1251, 1138, 1075.

HRMS (ESI$^+$) calcd. for $C_{22}H_{38}O_2SnNa$ $[M+Na]^+$ 477.1790, found 477.1784.

$[\alpha]_D^{21}$ –35.7 (c 1.00, $CHCl_3$).

Chiral HPLC: The e.r. was determined by HPLC [Daicel Chiralpak-IB (25 cm) with guard, 100% hexane, 0.9 mL/min, r.t., 210 nm, t_R (major) = 4.6 min, t_R (minor) = 6.1 min] to be 95.4:4.6.

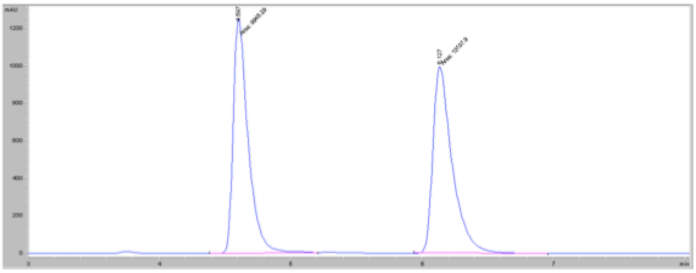

#	Time	Area	Height	Width	Area%	Symmetry
1	4.597	9946.3	1256.4	0.1319	49.474	0.543
2	6.127	10157.9	997.6	0.1697	50.526	0.529

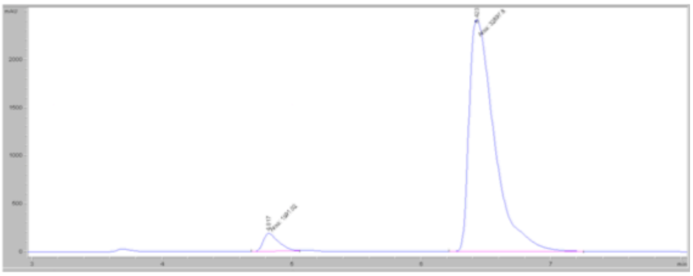

#	Time	Area	Height	Width	Area%	Symmetry
1	4.817	1591	190.7	0.139	4.613	0.541
2	6.423	32897.8	2418.7	0.2267	95.387	0.423

(2,4,5-Trimethoxyphenyl)methanol (S6)

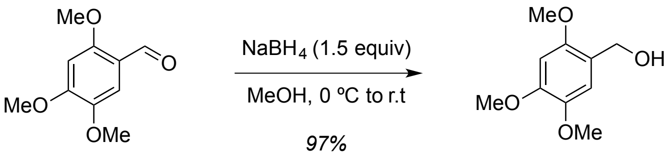

S6

Sodium borohydride (2.84 g, 75.0 mmol, 1.50 equiv) was added slowly to a solution of 2,4,5-trimethoxybenzaldehyde (9.81 g, 50.0 mmol, 1.00 equiv) in anhydrous methanol (200 mL) at 0 °C. After stirring for 18 h at room temperature the mixture was cooled to 0 °C, H_2O (50 mL) was added slowly and the solution was concentrated under reduced pressure. The residue was dissolved in Et_2O (50 mL) and water (50 mL) was added. The phases were separated and the aqueous layer was extracted with Et_2O (3 × 30 mL). The combined organic layers were washed with brine (15 mL), dried ($MgSO_4$), filtered and concentrated *in vacuo* to give the title compound (9.60 g, 48.4 mmol, 97%) as a white solid, which was used without further purification.

Mpt: 70–72 °C (Et_2O).

^{1}H NMR (500 MHz, $CDCl_3$) δ_H ppm 6.86 (s, 1H), 6.54 (s, 1H), 4.62 (s, 2H), 3.89 (s, 3H), 3.84 (s, 3H), 3.84 (s, 3H), 2.23 (br s, 1H) ppm.

^{13}C NMR (126 MHz, $CDCl_3$) δ_C 151.7, 149.2, 142.8, 120.6, 113.2, 97.3, 61.5, 56.6, 56.3, 56.1 ppm.

IR (ν_{max}/cm^{-1}, neat): 3481, 3360, 2936, 1609, 1509, 1201, 1122, 1030, 1001.

HRMS (CI^+) calcd. for $C_{10}H_{15}O_4$ $[M+H]^+$ 199.0970, found 199.0961.

2,4,5-Trimethoxybenzyl 2,4,6-triisopropylbenzoate (16)

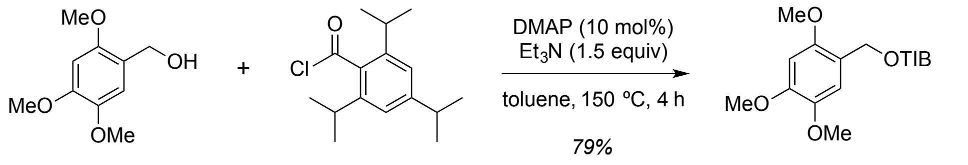

S6 (1.2 equiv) (1.0 equiv) **16**

(2,4,5-Trimethoxyphenyl)methanol (**S6**) (2.38 g, 12.0 mmol, 1.20 equiv), 2,4,6-triisopropylbenzoyl chloride (2.67 g, 10.0 mmol, 1.00 equiv) and 4-(dimethylamino)pyridine (122 mg, 1.00 mmol, 10.0 mol%) were dissolved in anhydrous toluene (10 mL) under an inert atmosphere in a microwave vial. Et_3N (2.08 mL, 15.0 mmol, 1.50 equiv) was added before the vial was sealed and heated for 4 h at 150 °C in a microwave reactor. After cooling to room temperature, the salts were removed by filtration through a plug of silica and the solids were thoroughly washed with Et_2O. The filtrate was concentrated *in vacuo* and the residue purified by FCC (SiO_2, 20% EtOAc/pentane) to afford the title compound (3.38 g, 7.89 mmol, 79%) as a white solid.

Mpt: 65–66 °C (pentane/EtOAc).

R_f: 0.36 (20% EtOAc/pentane).

^{1}H NMR (400 MHz, $CDCl_3$) δ_H 6.98 (s, 1H), 6.98 (s, 2H), 6.53 (s, 1H), 5.35 (s, 2H), 3.91 (s, 3H), 3.84 (s, 3H), 3.83 (s, 3H), 2.87 (hept, J = 6.8 Hz, 3H), 1.23 (d, J = 6.9 Hz, 6H), 1.20 (d, J = 6.8 Hz, 12H) ppm.

^{13}C NMR (101 MHz, $CDCl_3$) δ_C 171.7, 152.7, 150.3, 150.2, 144.9, 142.9, 130.7, 120.9, 115.3, 115.2, 97.2, 62.1, 56.8, 56.4, 56.3, 34.6, 31.5, 24.2, 24.1 ppm.

IR (ν_{max}/cm^{-1}, neat): 2962, 1715, 1606, 1515, 1464, 1212, 1131, 1032.

HRMS (EI^+) calcd. for $C_{26}H_{36}O_5$ $[M]^+$ 428.2563, found 428.2561.

2,4,5-Trimethoxybenzyl diisopropylcarbamate (S7)

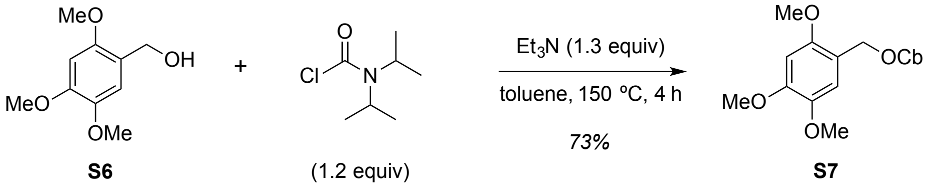

(2,4,5-Trimethoxyphenyl)methanol (**S6**) (1.98 g, 10.0 mmol, 1.00 equiv) and *N,N*-diisopropylcarbamoyl chloride (1.96 g, 12.0 mmol, 1.20 equiv) were dissolved in anhydrous toluene (10 mL) under an inert atmosphere in a microwave vial. Et_3N (1.80 mL, 13.0 mmol, 1.30 equiv) was added before the vial was sealed and heated for 4 h at 150 °C in a microwave reactor. After cooling to room temperature, the salts were removed by filtration through a plug of silica and the solids were thoroughly washed with Et_2O. The solvent was removed under reduced pressure and the residue was purified by FCC (SiO_2, 30% EtOAc/ pentane) to afford the title compound (2.39 g, 7.34 mmol, 73%) as a white solid.

Mpt: 79–81 °C (EtOAc/pentane).

R_f: 0.39 (30% EtOAc/pentane).

^{1}H NMR (400 MHz, $CDCl_3$) δ_H 6.91 (s, 1H), 6.52 (s, 1H), 5.11 (s, 2H), 4.06 (br m, 1H), 3.88 (s, 3H), 3.83 (br m, 1H), 3.82 (s, 3H), 3.80 (s, 3H), 1.19 (d, J = 6.8 Hz, 12H) ppm.

^{13}C NMR (101 MHz, $CDCl_3$) δ_C 155.8, 152.0, 149.3, 142.7, 116.9, 113.7, 97.5, 61.5, 56.5, 56.4, 56.1, 45.7 (br), 20.9 (br) ppm.

IR (ν_{max}/cm^{-1}, neat): 2969, 1671, 1519, 1438, 1287, 1208, 1129, 1029.

HRMS (ESI$^+$) calcd. for $C_{17}H_{21}NNaO_5$ $[M+Na]^+$ 348.1781, found 348.1770.

Chloromethyl 2,4,6-trimethylbenzoate (S8)

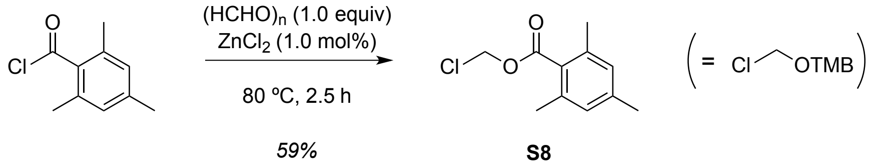

Prepared following a modified literature procedure.[12] To an oven-dried Schlenk tube under N_2 was added $ZnCl_2$ (41 mg, 0.30 mmol, 1.0 mol%). The flask was placed under vacuum and heated with a heat gun for 2 min to fuse the $ZnCl_2$. After allowing to cool to room temperature, the flask was backfilled with N_2 before the addition of paraformaldehyde (901 mg, 30.0 mmol, 1.00 equiv) and 2,4,6-trimethylbenzoyl chloride (5.00 mL, 30.0 mmol, 1.00 equiv). The resulting suspension was stirred and heated to 80 ºC for 2.5 h to give a pale yellow solution. The reaction was allowed to cool to room temperature, resulting in a white precipitate, before purification by FCC (SiO_2, 25% toluene/pet. ether) to give the title compound (3.75 g, 17.6 mmol, 59%) as a colourless oil.

R_f: 0.31 (25% toluene/pet. ether)

[12] D. Rennison, O. Laita, S. Bova, M. Cavalli, B. Hopkins, D. S. Linthicum, M. B. Brimble, *Bioorg. Med. Chem.* **2012**, *20*, 3997 – 4011.

^{1}**H NMR** (500 MHz, $CDCl_3$): δ_H 6.88 (s, 2H), 5.93 (s, 2H), 2.33 (s, 6H), 2.30 (s, 3H) ppm.

^{13}**C NMR** (126 MHz, $CDCl_3$): δ_C 167.9, 140.3, 135.9, 128.8, 128.7, 68.9, 21.2, 19.9 ppm.

IR (ν_{max}/cm^{-1}, neat): 2981–2864, 1612, 1440, 1748, 1242, 1161, 1056.

HRMS (EI^+) calcd. for $C_{11}H_{13}ClO_2$ $[M]^+$ 212.0610, found 212.0604.

Chloromethyl 2,4,6-triisopropylbenzoate (23)

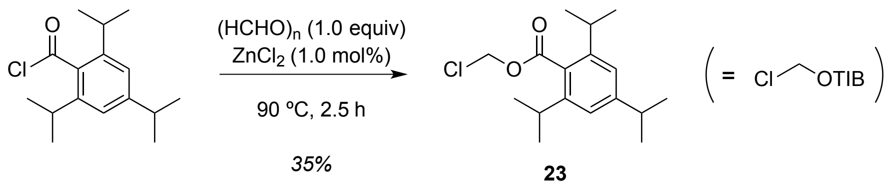

Prepared following a modified literature procedure.[12] To an oven-dried vial was added anhydrous $ZnCl_2$ (5.7 mg, 0.042 mmol, 1.0 mol%), followed by paraformaldehyde (126 mg, 4.18 mmol, 1.00 equiv) and 2,4,6-triisopropylbenzoyl chloride (1.12 g, 4.18 mmol, 1.00 equiv). The vial was purged with N_2 before sealing with a screw cap. The solid mixture was heated to 90 ºC to fuse the acid chloride and the resulting heterogeneous mixture was stirred for 2.5 h. The mixture was allowed to cool to room temperature before being purified by FCC (SiO_2, 1% Et_2O/pet. ether) to give the title compound (440 mg, 1.48 mmol, 35%) as a white solid.

Mpt: 53–55 ºC (Et_2O).

R$_f$: 0.34 (100% hexanes).

^{1}**H NMR** (400 MHz, $CDCl_3$): δ_H 7.03 (s, 2H), 5.92 (s, 2H), 2.91 (hept, J = 6.9 Hz, 1H), 2.89 (hept, J = 6.9 Hz, 2H), 1.26 (d, J = 6.9 Hz, 6H), 1.26 (d, J = 6.8 Hz, 12H) ppm.

^{13}**C NMR** (126 MHz, $CDCl_3$): δ_C 168.7, 151.0, 145.3, 128.3, 121.0, 68.8, 34.5, 31.3, 24.1, 23.9 ppm.

IR (ν_{max}/cm^{-1}, neat): 2963–2874, 1751, 1607, 1462, 1232, 1129, 1100, 1047.

HRMS (EI^+) calcd. for $C_{17}H_{25}ClO_2$ $[M]^+$ 296.1543, found 296.1556.

5. Synthesis of Neopentyl Glycol Boronic Ester 10

5,5-Dimethyl-2-((2*S*,3*R*)-3-(2,4,5-trimethoxyphenyl)pentan-2-yl)-1,3,2-dioxaborinane (10)

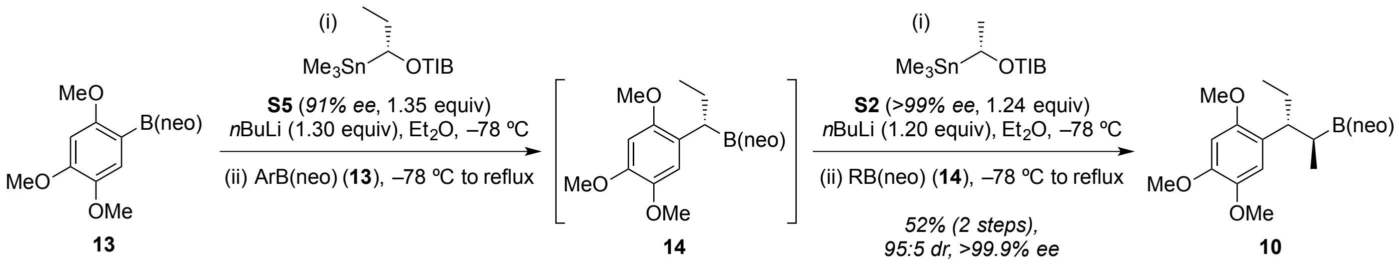

n-Butyllithium (1.60 M in hexanes, 2.44 mL, 3.90 mmol, 1.30 equiv) was added dropwise over 10 min to a solution of (*R*)-1-(trimethylstannyl)propyl 2,4,6-triisopropylbenzoate (**S5**) (1.84 g, 4.05 mmol, 1.35 equiv) in anhydrous Et_2O (15 mL) at −78 °C under an atmosphere of nitrogen and the reaction mixture was stirred for 1 h. A solution of 5,5-dimethyl-2-(2,4,5-trimethoxyphenyl)-1,3,2-dioxaborinane (**13**) (840 mg, 3.00 mmol, 1.00 equiv) in anhydrous Et_2O (10 mL) was added dropwise at −78 °C and the mixture was stirred at –78 °C for 1 h. The reaction mixture was then removed from the dry ice bath and heated under reflux for 16 h. After cooling to room temperature the mixture was filtered through a small plug of silica and the solids were thoroughly washed with Et_2O. The filtrate was concentrated under reduced pressure to give the crude boronic ester **14**, which was dried under high vacuum for 2 h before being re-dissolved in anhydrous Et_2O (10 mL).

In a second reaction vessel, *n*-butyllithium (1.60 M in hexanes, 2.25 mL, 3.60 mmol, 1.20 equiv) was added dropwise to a solution of (*R*)-1-(trimethylstannyl)ethyl 2,4,6-triisopropylbenzoate (**S2**) (1.64 g, 3.73 mmol, 1.24 equiv) in anhydrous Et_2O (15 mL) at −78 °C under an atmosphere of nitrogen and the mixture was stirred for 1 h. The solution of the crude boronic ester **14** was added dropwise at –78 °C and the mixture was stirred at –78 °C for 1 h. The reaction mixture was allowed to warm to room temperature and then heated under reflux for 4 h. After cooling to room temperature the mixture was filtered through a small plug of silica and the solids were thoroughly washed with Et_2O. The filtrate was concentrated *in vacuo*. The residue was purified by column chromatography (SiO_2, 25% EtOAc/pentane + 1% Et_3N) to give the title compound (572 mg, 1.63 mmol, 54%) as a white solid. The diastereomeric ratio was determined by ^{1}H NMR spectroscopy and accounted to 95:5. The enantiomeric excess was determined to be >99.9% by chiral HPLC analysis after oxidation to the corresponding alcohol (see below).

Mpt: 66–67 °C (pentane/EtOAc).

R_f: 0.15 (25% EtOAc/pentane + 1% Et_3N).

^{1}H NMR (500 MHz, $CDCl_3$) δ_H 6.65 (s, 1H), 6.51 (s, 1H), 3.87 (s, 3H), 3.81 (s, 3H), 3.76 (s, 3H), 3.64 (s, 4H), 2.94 (td, J = 10.8, 4.1 Hz, 1H), 1.67 – 1.62 (m, 1H), 1.55 – 1.51 (m, 1H), 1.13 (dq, J = 10.9, 7.3 Hz, 1H), 0.98 (s, 6H), 0.70 (d, J = 7.3 Hz, 3H), 0.68 (t, J = 7.3 Hz, 3H) ppm.

^{13}C NMR (126 MHz, $CDCl_3$) δ_C 152.8, 147.0, 143.4, 125.6, 111.5, 98.2, 72.1, 57.2, 56.6, 56.1, 42.0 (br), 31.8, 29.4, 27.2 (br), 22.1, 14.7, 12.4 ppm.

^{11}B NMR (96 MHz, $CDCl_3$) δ_B ppm 29.5 (br s).

IR (ν_{max}/cm^{-1}, neat): 2958, 1519, 1463, 1437, 1417, 1316, 1250, 1203, 1169, 1034.

HRMS (ESI$^+$) calcd. for $C_{12}H_{16}O_3I$ $[M+H]^+$ 335.0144, found 335.0151.

$[\alpha]_D^{21}$ –15.2 (*c* 1.05, $CHCl_3$).

(2*S*,3*S*)-3-(2,4,5-Trimethoxyphenyl)pentan-2-ol (S9)

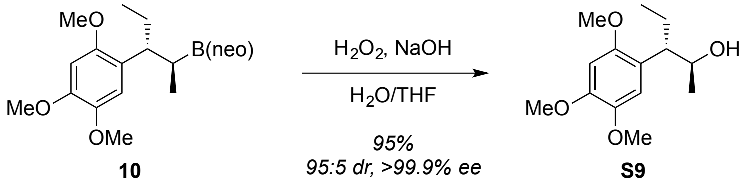

To a solution of neopentyl boronic ester **10** (48 mg, 0.14 mmol) in THF (3.0 mL) at 0 ºC was added 2.0 M aqueous NaOH (3.0 mL) followed by 30% aqueous H_2O_2 (1.5 mL). The mixture was stirred vigorously at room temperature 2 h before diluting with water (10 mL) and extracting with Et_2O (3 × 10 mL). The combined organic extracts were washed with brine (10 mL), dried ($MgSO_4$), filtered, and concentrated *in vacuo*. The residue was purified by FCC (SiO_2, 30% EtOAc/pet. ether) to give the title compound (33 mg, 0.13 mmol, 95%) as a colourless oil.

Mpt (racemate): 73–75 ºC (Et_2O).

R$_f$: 0.23 (30% EtOAc/pet. ether).

^{1}H NMR (500 MHz, $CDCl_3$) δ_H 6.65 (s, 1H), 6.55 (s, 1H), 3.96 (dq, *J* = 6.2, 6.2 Hz, 1H, 3.89 (s, 3H), 3.84 (s, 3H), 3.80 (s, 3H), 3.00 (ddd, *J* = 10.7, 6.3, 4.4 Hz, 1H), 2.00 (br s, 1H), 1.89 (dqd, *J* = 13.5, 7.5, 4.4 Hz, 1H), 1.63 (ddq, *J* = 13.5, 10.8, 7.3 Hz, 1H), 1.05 (d, *J* = 6.2 Hz, 3H), 0.79 (t, *J* = 7.4 Hz, 3H) ppm.

^{13}C NMR (126 MHz, $CDCl_3$) δ_C 152.2, 147.8, 143.2, 121.8, 112.9, 98.1, 71.5, 56.8, 56.7, 56.0, 47.3, 22.8, 20.5, 12.3 ppm.

IR (ν_{max}/cm^{-1}, neat): 3421, 2962–2833, 1509, 1465, 1397, 1315, 1205, 1036.

HRMS (ESI$^+$) calcd. for $C_{14}H_{22}O_4Na$ $[M+Na]^+$ 277.1410, found 277.1408.

$[\alpha]_D^{24}$ +19.3 (*c* 1.50, $CHCl_3$).

Chiral HPLC: The e.r. was determined by HPLC [Daicel Chiralpak-IB (25 cm) with guard, 7:93 *i*-PrOH/hexanes, 1.0 mL/min, r.t., 210 nm, t_R (major) = 9.1 min, t_R (minor) = 10.2 min] to be >99.9:0.1.

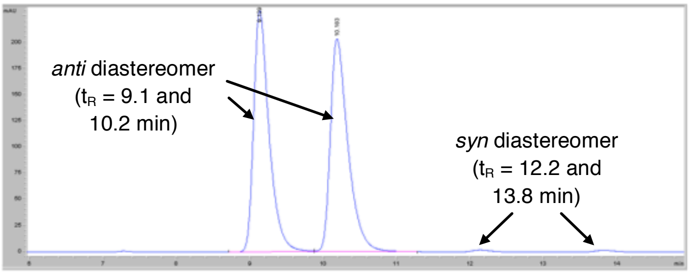

#	Time	Area	Height	Width	Area%	Symmetry
1	9.129	3390.1	230.5	0.2196	49.840	0.601
2	10.183	3411.9	203.6	0.2508	50.160	0.56

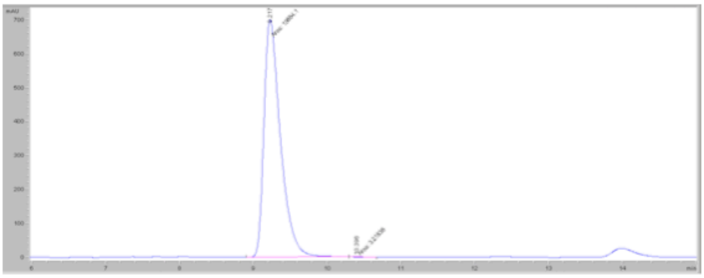

#	Time	Area	Height	Width	Area%	Symmetry
1	9.217	10864.1	700.6	0.2585	99.970	0.562
2	10.396	3.2	2.4E-1	0.2192	0.030	0

For the preparation of racemic **S9**, see Section 9.

6. Ligand Studies in Lithiation-Borylations with Primary Benzylic TIB-ester 16

Optimization of Enantioselective Lithiation-Borylations with Achiral Boronic Esters

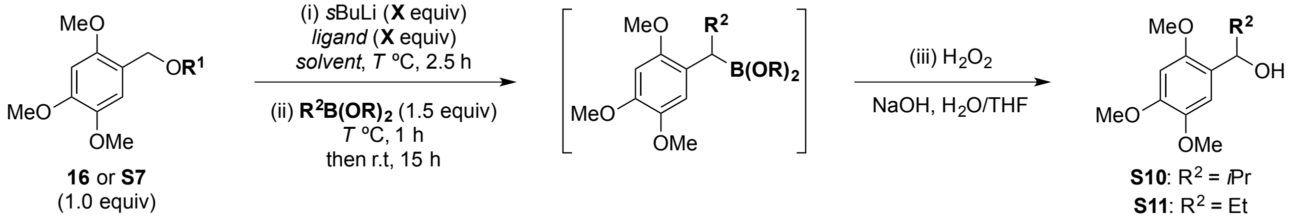

Table S1:

Entry[a]	R^1	$R^2B(OR)_2$	*ligand*	*solvent*	*T* (ºC)	% yield[c]	% ee[d]
1	TIB	*i*PrB(neo)	(+)-sparteine	Et_2O	–78	94	–34
2	TIB	*i*PrB(neo)	(+)-sparteine	Et_2O	–95	90	–40
3	TIB	*i*PrB(neo)	(+)-sparteine	toluene	–78	48	–37
4	Cb	*i*PrB(neo)	(+)-sparteine	toluene	–78	77	18
5	TIB	*i*PrB(neo)	(*S*,*S*)-*t*Bu-BOX	toluene	–78	69	58
**6**	**TIB**	***i*PrB(neo)**	**(*S*,*S*)-*t*Bu-BOX**	**toluene**	**–95**	**67**	**86**
7	TIB	*i*PrB(neo)	(*S*,*S*)-*t*Bu-BOX	Et_2O	–95	58	2
8	TIB	*i*PrB(neo)	(*S*,*S*)-*t*Bu-BOX	*t*BuOMe/toluene[b]	–95	42	12
9	Cb	*i*PrB(neo)	(*S*,*S*)-*t*Bu-BOX	toluene	–95	33	23
10	TIB	EtB(pin)	(*S*,*S*)-*t*Bu-BOX	toluene	–78	62	5
11	TIB	EtB(pin)	(*S*,*S*)-*t*Bu-BOX	toluene	–95	40	9
12	Cb	EtB(pin)	(*S*,*S*)-*t*Bu-BOX	toluene	–78	25	7

[a] Reactions performed using general procedure outlined below. [b] A 1:1 ratio of *t*BuOMe/toluene was used. [c] Yield of isolated product after purification by FCC. [d] Determined by chiral SFC analysis. The absolute configurations of **S10** and **S11** were not determined.

General Procedure for Table S1: A solution of benzylic TIB-ester **16** (0.10 mmol, 1.0 equiv) or benzylic carbamate **S7** (0.10 mmol, 1.0 equiv) and the diamine ligand (0.13 mmol, 1.3 equiv) in anhydrous solvent (1.0 mL) was cooled to temperature *T* °C before the addition of *s*BuLi (1.3 M, 0.10 mL, 0.13 mmol, 1.3 equiv) dropwise over 2 min. The reaction was stirred at *T* ºC for 1 h (when using sparteine) or 2.5 h (when using *t*Bu-BOX) before the addition of a solution of *i*PrB(neo) (0.15 mmol, 1.5 equiv) or EtB(pin) (0.15 mmol, 1.5 equiv) in anhydrous solvent (0.20 mL) dropwise over 1 min. The mixture was stirred for 2 h at *T* °C before the cooling bath was removed and stirring was continued at room temperature for 15 h. After this time, the solvent was removed under reduced pressure and the residue was re-dissolved in THF (2.0 mL), cooled to 0 ºC and treated with 2.0 M aqueous NaOH (2.0 mL) and 30% aqueous H_2O_2 (1.0 mL). The mixture was stirred vigorously at room temperature 2 h before diluting with water (10 mL) and extracting with Et_2O (3 × 10 mL). The combined organic extracts were washed with brine (10 mL), dried ($MgSO_4$), filtered, and concentrated *in vacuo*. The residue was purified by FCC (SiO_2, 30% EtOAc/pentane) to give the benzylic alcohol product.

2-Methyl-1-(2,4,5-trimethoxyphenyl)propan-1-ol (S10)

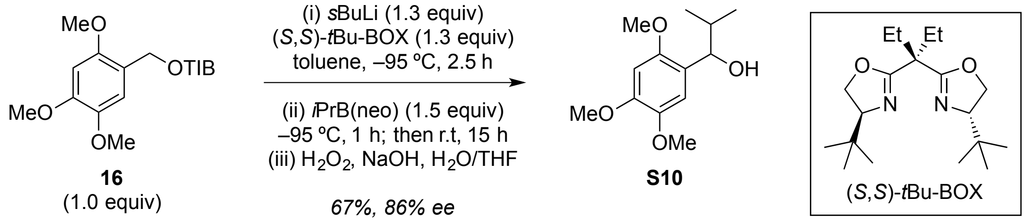

Prepared following the general procedure for Table S1, using benzylic TIB-ester **16** (43 mg, 0.10 mmol), (*S*,*S*)-*t*Bu-BOX (42 mg, 0.13 mmol), *s*BuLi (1.3 M, 0.10 mL, 1.3 mmol) and *i*PrB(neo) (23 mg, 0.15 mmol) in anhydrous toluene. Purification by FCC (SiO_2, 30% EtOAc/pentane) provided the title compound as a colourless oil (16 mg, 0.067 mmol, 67%, 86% ee).

The racemic product was prepared in an analogous fashion using TMEDA in place of (*S*,*S*)-*t*Bu-BOX.

R_f: 0.23 (30% EtOAc/pentane).

^{1}H NMR (400 MHz, $CDCl_3$) δ_H 6.85 (s, 1H), 6.52 (s, 1H), 4.51 (d, *J* = 5.7 Hz, 1H), 3.89 (s, 3H), 3.85 (s, 3H), 3.82 (s, 3H), 2.34 (br s, 1H), 2.00 (dhep, *J* = 6.7, 0.9 Hz, 1H), 1.04 (d, *J* = 6.7 Hz, 3H), 0.80 (d, *J* = 6.8 Hz, 3H) ppm.

^{13}C NMR (126 MHz, $CDCl_3$) δ_C 150.9, 148.5, 143.0, 123.4, 111.9, 97.4, 75.9, 56.6, 56.2, 56.2, 34.5, 19.4, 18.6 ppm.

IR (ν_{max}/cm^{-1}, neat): 3472, 2957–2833, 1611, 1508, 1463, 1439, 1398, 1201, 1176, 1132, 1030.

HRMS (CI^+) calcd. for $C_{13}H_{21}O_3$ $[M+H]^+$ 241.1440, found 241.1445.

Chiral SFC: The e.r. was determined by SFC [Daicel Chiralpak-IB (25 cm), 5% MeOH, 4.0 mL/min, 40 ºC, 125 bar, t_R (major) = 3.3 min, t_R (minor) = 4.5 min] to be 92.8:7.2.

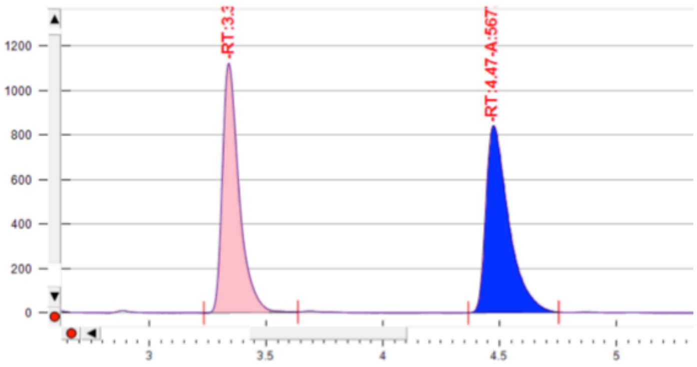

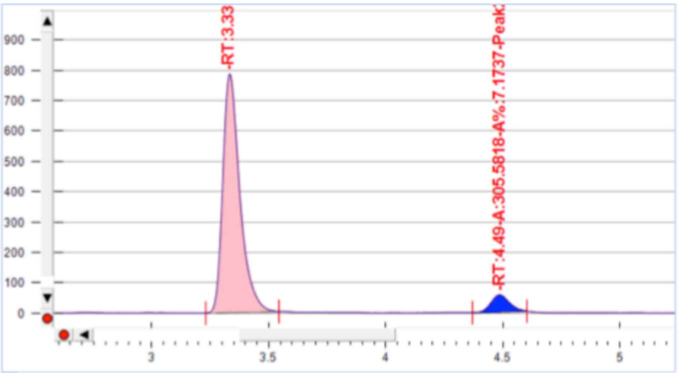

Peak No	% Area	Area	Ret. Time	Height	Cap. Factor
1	49.385	5539.5435	3.34 min	1121.0013	3339
2	50.615	5677.5041	4.47 min	840.868	4472.2833

Peak No	% Area	Area	Ret. Time	Height	Cap. Factor
1	92.8263	3954.1801	3.33 min	786.7422	3332.3333
2	7.1737	305.5818	4.49 min	56.1769	4485.6167

1-(2,4,5-Trimethoxyphenyl)propan-1-ol (S11)

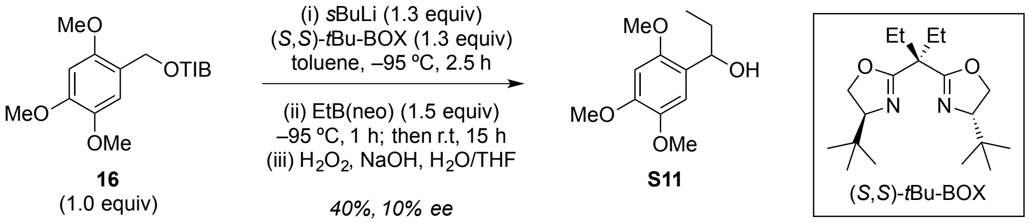

Prepared following the general procedure for Table S1, using benzylic TIB-ester **16** (43 mg, 0.10 mmol), (*S*,*S*)-*t*Bu-BOX (42 mg, 0.13 mmol), *s*BuLi (1.3 M, 0.10 mL, 1.3 mmol) and EtB(pin) (23 mg, 0.15 mmol) in anhydrous toluene. Purification by FCC (SiO_2, 30% EtOAc/pentane) provided the title compound as a colourless oil (9.0 mg, 0.040 mmol, 40%, 9% ee).

Racemic **S11** was prepared as follows: To a solution of 2,4,5-trimethoxybenzaldehyde (1.96 g, 10.0 mmol, 1.0 equiv) in anhydrous toluene (30 mL) at room temperature was slowly added ethylmagnesium bromide solution (3.0 M in Et_2O, 5.0 mL, 15 mmol, 1.5 equiv). The mixture was then stirred at room temperature for 20 h before cooling to 0 ºC and quenching by the slow addition of 2% aqueous NH_4Cl (10 mL). The mixture was stirred for 10 min at room temperature before the layers were separated and the aqueous phase extracted with Et_2O (3 × 10 mL). The combined organic extracts were washed with brine (15 mL), dried ($MgSO_4$), filtered and concentrated *in vacuo*. Purification by FCC (SiO_2, 30% EtOAc/pentane) provided the title compound as a white solid (2.11 g, 9.33 mmol, 93%).

R_f: 0.18 (30% EtOAc/pentane).

Mpt (racemic): 68–69 ºC (EtOAc/pentane)

^{1}H NMR (500 MHz, $CDCl_3$) δ_H 6.89 (s, 1H), 6.52 (s, 1H), 4.78 (t, J = 6.7 Hz, 1H), 3.89 (s, 3H), 3.85 (s, 3H), 3.83 (s, 3H), 2.40 (br s, 1H), 1.85–1.72 (m, 2H), 0.95 (t, J = 7.4 Hz, 3H) ppm.

^{13}C NMR (101 MHz, $CDCl_3$) δ_C 150.8, 148.6, 143.1, 124.1, 111.1, 97.5, 71.6, 56.6, 56.21, 56.19, 30.5, 10.4 ppm.

IR (ν_{max}/cm^{-1}, neat): 3417, 2954, 1659, 1609, 1512, 1450, 1402, 1208, 1029.

HRMS (CI^+) calcd. for $C_{12}H_{19}O_4$ $[M+H]^+$ 227.1283, found 227.1289.

Chiral SFC: The e.r. was determined by SFC [Daicel Chiralpak-IB (25 cm), 5% MeOH, 4.0 mL/min, 40 ºC, 125 bar, t_R (major) = 3.8 min, t_R (minor) = 4.8 min] to be 54.5:45.5.

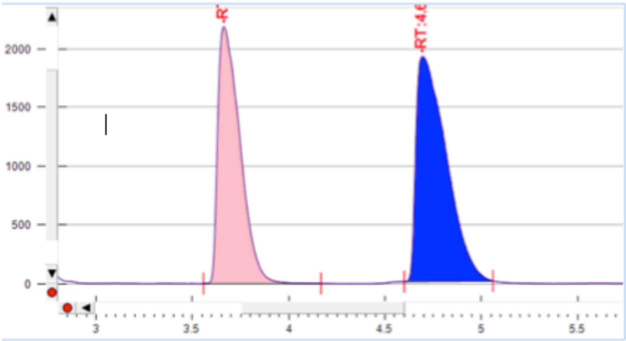

Peak No	% Area	Area	Ret. Time	Height	Cap. Factor
1	48.4977	17393.9045	3.66 min	2190.0384	3662.3333
2	51.5023	18471.5044	4.69 min	1867.2665	4692.2667

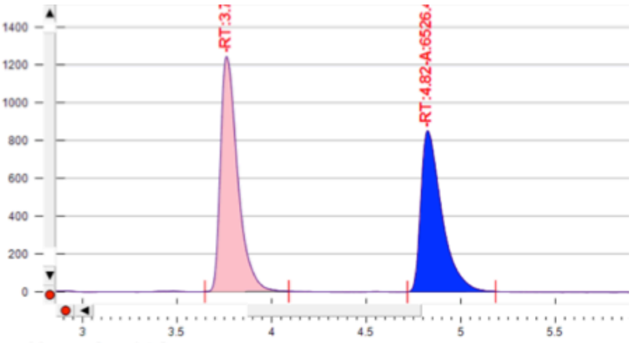

Peak No	% Area	Area	Ret. Time	Height	Cap. Factor
1	54.4506	7801.8832	3.76 min	1240.2997	3759
2	45.5494	6526.4904	4.82 min	851.1514	4822.2667

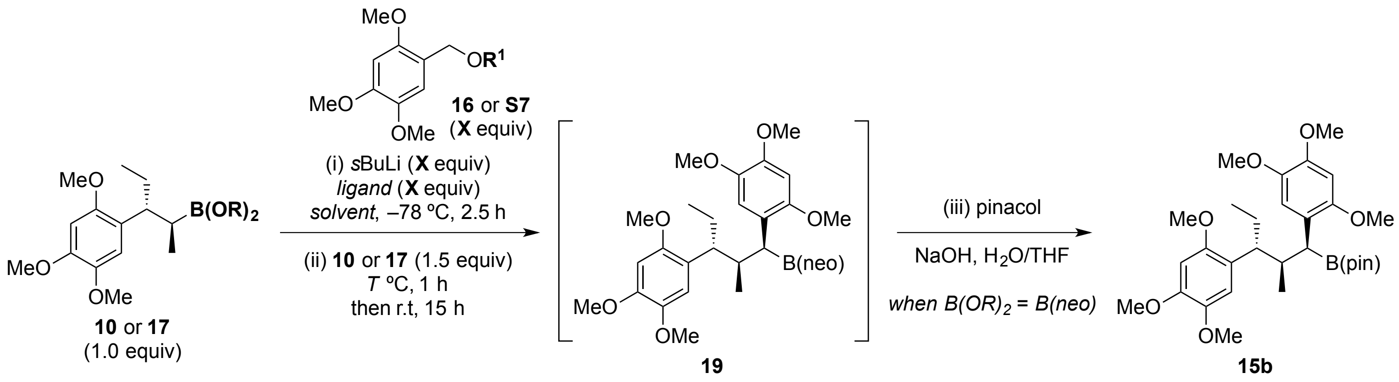

Table S2:

Entry[a]	(OR)₂	R[1]	*ligand*	**X** equiv	*solvent*	% conv.[d]	crude dr (*syn*:*anti*)[e]	% yield[f]	purified dr (*syn*:*anti*)[e]
1[b]	neo	TIB	(*S*,*S*)-*t*Bu-BOX	1.2	toluene	<5	-	-	-
2[b]	neo	TIB	(*S*,*S*)-*i*Pr-BOX	1.5	toluene	95	92:8	72	94:6
3[b]	neo	TIB	(*R*,*R*)-*i*Pr-BOX	1.5	toluene	72	88:12	52	88:12
4[b]	neo	TIB	TMEDA	1.5	toluene	77	98:2	61	98:2
5[b]	pin	TIB	TMEDA	1.5	toluene	32	47:53	29	47:53
6[c]	neo	TIB	(*S*,*S*)-*i*Pr-BOX	2.0	Et_2O	59	86:14	ND	ND
7[c]	neo	TIB	(*R*,*R*)-*i*Pr-BOX	2.0	Et_2O	63	58:42	ND	ND
8[c]	neo	TIB	TMEDA	2.0	Et_2O	95	91:9	ND	ND
9[c]	neo	Cb	TMEDA	2.0	Et_2O	58	88:12	ND	ND

[a] Reactions performed using general procedure outlined below. [b] Reactions performed using 0.2 mmol of boronic ester **10** or **17**. [c] Reaction performed using 0.05 mmol of boronic ester **10**. [d] Conversion of boronic ester **10** or **17** into **15** (entries 1–5) or **19** (entries 6–9). Determined by ^{1}H NMR analysis of the crude reaction mixture. [e] Determined by ^{1}H NMR analysis. [f] Isolated yield after purification by FCC.

We believe the difference in the levels of selectivity between the neopentyl glycol and pinacol boronic esters is related to a change in the selectivity-determining step of the reaction. In the case of pinacol esters, boronate complex formation is reversible making 1,2-migration the stereochemistry-determining step.[13] For the less sterically hindered neopentyl glycol boronic ester **10**, boronate complex formation is non-reversible,[14] thus making it the stereochemistry- determining step and resulting in high diastereoselectivity.

General Procedure for Table S2: To a solution of benzylic TIB-ester **16** (0.24–0.40 mmol, 1.2–2.0 equiv), or benzylic carbamate **S7** (0.24–0.40 mmol, 1.2–2.0 equiv), and ligand (0.24–0.40 mmol, 1.2–2.0 equiv) in anhydrous solvent (3.0 mL) at –78 ºC was added *s*BuLi (1.3 M, 0.24–0.40 mmol, 1.2–2.0 equiv) dropwise over 2 min. The reaction mixture was stirred at –78 ºC for 2.5 h before adding a solution of neopentyl glycol boronic ester **10** (0.20 mmol, 1.0 equiv), or pinacol boronic ester **17** (0.20 mmol, 1.0 equiv), in anhydrous solvent (1.0 mL) dropwise over 5 min. The resulting mixture was stirred at –78 ºC for a further 1 h before

[13] a) S. C. Matthew, B. W. Glasspoole, O. Eisenberger, C. M. Crudden, *J. Am. Chem. Soc.* **2014**, *136*, 5828 – 5831; b) V. Bagutski, R. M. French, V. K. Aggarwal, *Angew. Chem. Int. Ed.* **2010**, *49*, 5142 – 5145; c) B. M. Partridge, L. Chausser-Boissarie, M. Burns, A. P. Pulis, V. K. Aggarwal, *Angew. Chem. Int. Ed.* **2012**, *51*, 11795 – 11799.
[14] a) S. Roesner, D. J. Blair, V. K. Aggarwal, *Chem. Sci.* **2015**, *6*, 3718 – 3723; b) V. Bagutski, R. M. French, V. K. Aggarwal, *Angew. Chem. Int. Ed.* **2010**, *49*, 5142 – 5145.

removing from the cold bath and stirring at room temperature for 15 h (*For entries 6–9, Table S2, work-up was performed at this stage and the crude neopentyl glycol boronic ester **19** analyzed by ^{1}H NMR*). The solvent was removed under reduced pressure and the residue was re-dissolved in THF (2.5 mL). Pinacol (71 mg, 0.60 mmol, 3.0 equiv) and NaOH (0.25 mL, 0.2 M in water) were added subsequently and the reaction mixture was stirred vigorously for 2 h at room temperature. Water (10 mL) and Et_2O (10 mL) were added, the phases were separated, and the aqueous phase was re-extracted with Et_2O (2 × 10 mL). The combined organic phases were washed with brine (20 mL), dried (Na_2SO_4), filtered and the solvent removed *in vacuo*. The crude product was analyzed by ^{1}H NMR before being purified by FCC (2 × SiO_2, 1st = 30% EtOAc/pet. ether, 2nd = 2% acetone/CH_2Cl_2) to give secondary pinacol boronic ester **15** as a colourless viscous oil.

4,4,5,5-Tetramethyl-2-((1*S*,2*R*,3*R*)-2-methyl-1,3-bis(2,4,5-trimethoxyphenyl)pentyl)-1,3,2-dioxaborolane (15b)

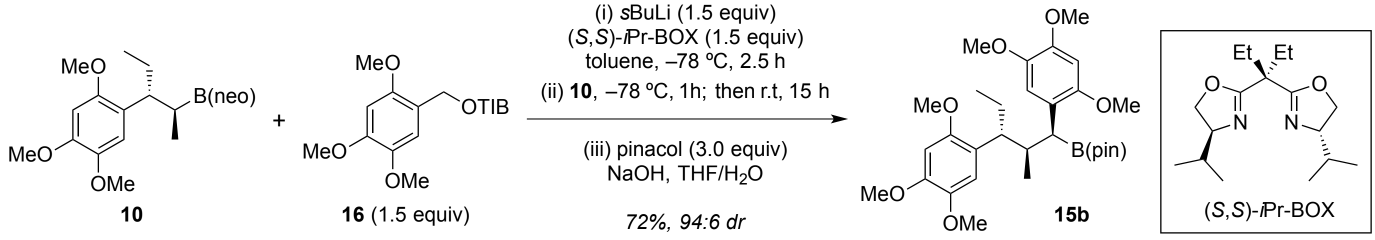

Prepared following the general procedure for Table S2, using 2,4,5-trimethoxybenzyl 2,4,6-triisopropylbenzoate (**16**) (128 mg, 0.300 mmol, 1.50 equiv), (*S*,*S*)-*i*Pr-BOX (**L***) (88 mg, 0.30 mmol, 1.5 equiv), *s*BuLi (1.3 M, 0.23 mL, 0.30 mmol, 1.5 equiv), neopentyl glycol boronic ester **10** (95:5 *dr*, 70 mg, 0.20 mmol, 1.0 equiv) and anhydrous toluene (3.0 mL + 1.0 mL). Transesterification was performed using pinacol (71 mg, 0.60 mmol, 3.0 equiv) and NaOH (0.25 mL, 0.2 M in water) in THF (2.5 mL). Purification by FCC (2 × SiO_2, 1st = 30% EtOAc/pet. ether, 2nd = 2% acetone/CH_2Cl_2) gave the title compound (78 mg, 0.14 mmol, 72%) as a colourless viscous oil. The diastereoselectivity for the reaction was 94:6, as determined by ^{1}H NMR. This ratio could be improved to >95:5 by careful separation of the diastereomers by FCC (SiO_2, 2% acetone/CH_2Cl_2).

R_f: 0.29 (30% EtOAc/pet. ether), 0.30 (2% Acetone/CH_2Cl_2).

^{1}H NMR (500 MHz, $CDCl_3$) δ_H 6.75 (s, 1H), 6.68 (s, 1H), 6.52 (s, 1H), 6.46 (s, 1H), 3.89 (s, 3H), 3.85 (s, 3H), 3.83 (s, 3H), 3.81 (s, 3H), 3.75 (s, 3H), 3.67 (s, 3H), 3.06 (br s, 1H), 2.56 (d, *J* = 9.6 Hz, 1H), 2.34–2.24 (m, 1H), 1.88 (dqd, *J* = 13.2, 7.4, 5.1 Hz, 1H), 1.92–1.83 (m, 1H), 1.73–1.58 (m, 1H), 1.26 (s, 6H), 1.20 (s, 6H), 0.77 (t, *J* = 7.3 Hz, 3H), 0.45 (d, *J* = 6.9 Hz, 3H) ppm.

^{13}C NMR (126 MHz, $CDCl_3$) δ_C 152.8, 152.2, 147.3, 147.0, 142.87, 142.87, 124.0, 122.5, 114.6, 113.4 (br), 98.4, 97.9, 82.9, 57.0, 56.9, 56.7, 56.6, 56.14, 56.11, 43.3 (br), 39.5, 29.0 (br), 27.2 (br), 25.1, 24.7, 15.6, 12.9 ppm.

^{11}B NMR (96 MHz, $CDCl_3$) δ_B 32.2 (br s) ppm.

IR (ν_{max}/cm^{-1}, neat): 2961–2830, 1508, 1462, 1309, 1202, 1142, 1034.

HRMS (ESI^{+}) calcd. for $C_{30}H_{45}O_8BNa$ [M+Na]$^{+}$ 567.3105, found 567.3100.

$[\alpha]_D^{21}$ +26.0 (*c* 0.73, $CHCl_3$).

7. End Game Studies: Stereospecific Olefinations Using Suzuki and Zweifel Reactions

*Synthesis of vinyl iodide **4** and vinyl chloride **S14***

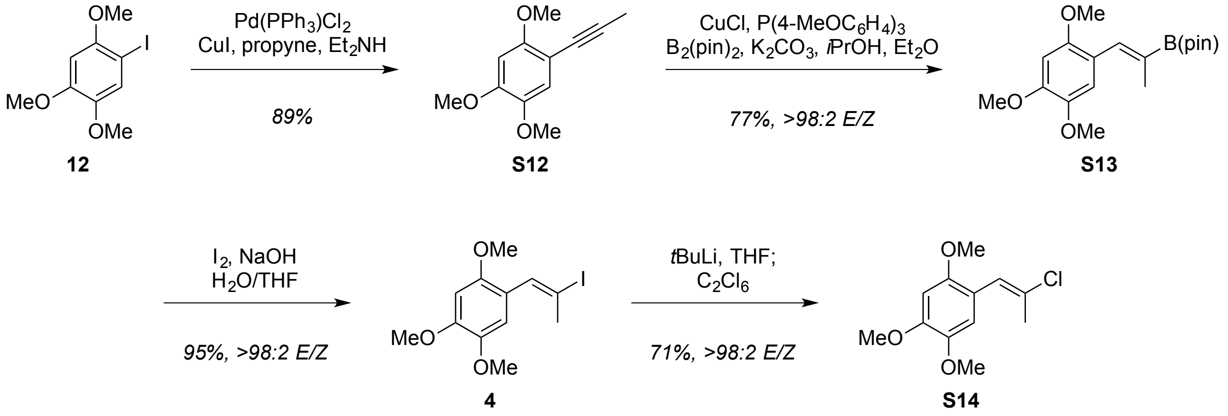

1,2,4-Trimethoxy-5-(prop-1-yn-1-yl)benzene (S12)

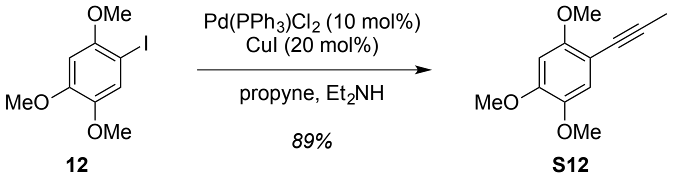

1-Iodo-2,4,5-trimethoxybenzene (**12**) (294 mg, 1.00 mmol, 1.0 equiv), copper (I) iodide (38 mg, 0.20 mmol, 0.2 equiv) and bis(triphenylphosphine)palladium (II) chloride (70 mg, 0.10 mmol, 0.1 equiv) were dissolved in diethylamine (3.5 mL). The solution was sparged with an excess of propyne (balloon) for 5 min before replacing the propyne balloon with a nitrogen balloon. The reaction mixture was stirred at room temperature under N_2 for 12 h. The solvent was removed *in vacuo* and the residue was dissolved in CH_2Cl_2 (20 mL) and water (20 mL) was added. The phases were separated and the aqueous layer was extracted with CH_2Cl_2 (2 × 15 mL). The combined organic layers were washed with brine (25 mL), dried ($MgSO_4$), filtered through charcoal and concentrated *in vacuo*. The crude product was purified by FCC (SiO_2, 10%–20% EtOAc/pentane) and recrystallized from Et_2O to give the title compound (184 mg, 0.892 mmol, 89%) as colourless needles.

Mpt: 89–91 °C (Et_2O).

R_f: 0.25 (20% EtOAc/pentane).

^{1}H NMR (400 MHz, $CDCl_3$) δ_H 6.90 (s, 1H), 6.49 (s, 1H), 3.90 (s, 3H), 3.88 (s, 3H), 3.83 (s, 3H), 2.12 (s, 3H) ppm.

^{13}C NMR (126 MHz, $CDCl_3$) δ_C 155.0, 149.6, 142.8, 116.2, 104.1, 97.2, 88.5, 75.7, 56.7, 56.4, 56.0, 4.8 ppm.

IR (ν_{max}/cm^{-1}, neat): 2914, 2837, 1517, 1461, 1395, 1345, 1232, 1203, 1150, 1026.

HRMS (CI^+) calcd. for $C_{12}H_{15}O_3$ $[M+H]^+$ 207.1021, found 207.1022.

(*Z*)-4,4,5,5-Tetramethyl-2-(1-(2,4,5-trimethoxyphenyl)prop-1-en-2-yl)-1,3,2-dioxaborolane (S13)

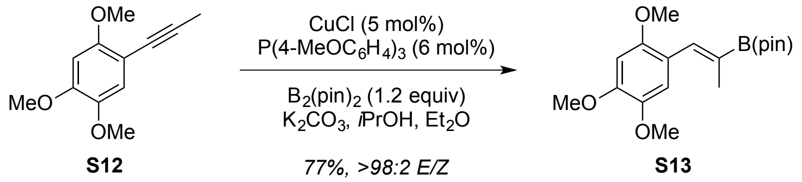

Following a modified literature procedure.[15] 1,2,4-trimethoxy-5-(prop-1-yn-1-yl)benzene (**S12**) (89 mg, 0.43 mmol, 1.0 equiv), bis(pinacolato)diboron (132 mg, 0.520 mmol, 1.2 equiv), tri(4-methoxyphenyl)phosphine (9.0 mg, 26 µmol, 6.0 mol%), copper (I) chloride (2.2 mg, 22 µmol, 5.0 mol%) and K_2CO_3 (12 mg, 86 µmol, 20 mol%) were dissolved in anhydrous Et_2O (2.0 mL). *i*-PrOH (67 µL, 0.86 mmol, 2.0 equiv) was added and the mixture was stirred for 15 h at ambient temperature. Et_2O (15 mL) and water (15 mL) were added, the phases were separated and the aqueous layer was extracted with Et_2O (2 × 15 mL). The combined organic layers were washed with brine (25 mL), dried ($MgSO_4$), filtered and concentrated *in vacuo*. The crude product was purified by FCC (SiO_2, 10%–20% EtOAc/pentane) to give the title compound (110 mg, 0.329 mmol, 77%) as a white solid.

Mpt: 86–88 °C ($CHCl_3$).

R$_f$: 0.28 (20% EtOAc/pentane).

^{1}H NMR (400 MHz, $CDCl_3$) δ_H 7.34 (q, *J* = 1.8 Hz, 1H), 6.92 (s, 1H), 6.53 (s, 1H), 3.91 (s, 3H), 3.83 (s, 3H), 3.83 (s, 3H), 1.94 (d, *J* = 1.8 Hz, 3H), 1.31 (s, 12H) ppm.

^{13}C NMR (126 MHz, $CDCl_3$) δ_C 152.0, 149.1, 142.2, 137.5, 118.5, 114.2, 97.1, 83.3, 56.6, 56.3, 56.0, 24.9, 16.2 ppm.

^{11}B NMR (96 MHz, $CDCl_3$) δ_B 29.6 (br s) ppm.

IR (ν_{max}/cm^{-1}, neat): 2978, 1592, 1517, 1312, 1300, 1269, 1206, 1145, 1092, 1030.

HRMS (CI$^+$) calcd. for $C_{18}H_{28}BO_5$ [M+H]$^+$ 335.2030, found 335.2019.

(*E*)-1-(2-Iodoprop-1-en-1-yl)-2,4,5-trimethoxybenzene (4)

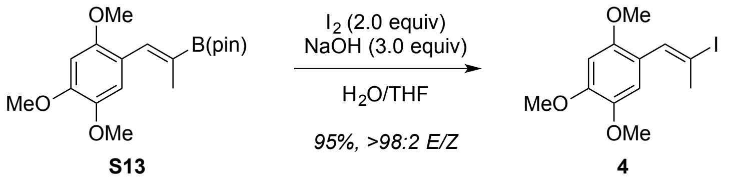

Following a modified literature procedure.[16] NaOH (3.0 M in water 0.620 mL, 1.86 mmol, 3.00 equiv) was added to a solution of vinyl boronic ester **S13** (207 mg, 0.619 mmol, 1.00 equiv) in THF (1.2 mL). After stirring for 10 min at room temperature a solution of iodine (314 mg, 1.24 mmol, 2.00 equiv) in THF 6.2 mL) was added over 5 min and stirring was continued for 1 h. The mixture was then quenched with saturated aqueous $Na_2S_2O_4$ (10 mL). The mixture was extracted with Et_2O (3 × 15 mL), the combined organic layers were washed with saturated aqueous $NaHCO_3$ solution (20 mL) and brine (20 mL), dried

[15] W. Yuan, S. Ma, *Org. Biomol. Chem.* **2012**, *10*, 7266 – 7268.
[16] C. Wang, T. Tobrman, Z. Xu, E. Negishi, *Org. Lett.* **2009**, *11*, 4092 – 4095.

($MgSO_4$), filtered and concentrated *in vacuo*. The residue was purified by FCC (SiO_2, 10%–20% EtOAc/pentane) to afford the title compound (197 mg, 0.590 mmol, 95%, >98:2 *E*:*Z*) as a pale yellow oil [N.B. The compound should be stored at –20 ºC as significant isomerization from >98:2 to 40:60 *E*:*Z* was observed over a period of 2 days when stored at r.t.].

R$_f$: 0.31 (20% EtOAc/pentane).

^{1}H NMR (500 MHz, $CDCl_3$) δ_H 7.22 (q, J = 1.8 Hz, 1H), 6.73 (s, 1H), 6.50 (s, 1H), 3.90 (s, 3H), 3.83 (s, 3H), 3.81 (s, 3H), 2.58 (d, J = 1.6 Hz, 3H) ppm.

^{13}C NMR (126 MHz, $CDCl_3$) δ_C 151.2, 149.3, 142.5, 136.2, 117.9, 113.5, 97.3, 97.0, 56.7, 56.4, 56.1, 29.5 ppm.

IR (ν_{max}/cm^{-1}, neat): 2942, 1508, 1454, 1318, 1202, 1030.

HRMS (CI$^+$) calcd. for $C_{12}H_{16}O_3I$ $[M+H]^+$ 335.0144, found 335.0151.

(*E*)-1-(2-Chloroprop-1-en-1-yl)-2,4,5-trimethoxybenzene (S14)

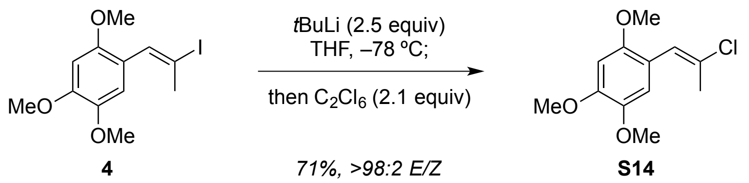

Following a modified literature procedure.[17] To a solution of vinyl iodide **4** in anhydrous THF (10.6 mL) at –78 ºC was added *t*BuLi (1.7 M in pentane, 0.784 mL, 1.33 mmol, 2.5 equiv) dropwise over 2 min. The resulting bright yellow solution was stirred at –78 ºC for 15 min before the dropwise addition of a solution of hexachloroethane (265 mg, 1.12 mmol, 2.1 equiv) in THF (2.1 mL) dropwise over 5 min. The mixture was stirred for 45 min at –78 ºC before saturated aqueous NH_4Cl (10 mL) was added and the mixture allowed to warm to room temperature. The phases were separated and the aqueous extracted with Et_2O (2 × 15 mL). The combined organic layers were washed with brine (20 mL), dried ($MgSO_4$), filtered and concentrated *in vacuo*. The residue was purified by FCC (SiO_2, 20% Et_2O/pet. ether) to afford the title compound (92 mg, 0.37 mmol, 71%, >98:2 *E*:*Z*) as a colourless oil.

R$_f$: 0.23 (20% Et_2O/pet. ether).

^{1}H NMR (500 MHz, $CDCl_3$) δ_H 6.72 (s, 1H), 6.69 (s, 1H), 6.52 (s, 1H), 3.91 (s, 3H), 3.84 (s, 3H), 3.82 (s, 3H), 2.23 (s, 3H) ppm.

^{13}C NMR (126 MHz, $CDCl_3$) δ_C 151.5, 149.3, 142.6, 131.6, 123.5, 116.3, 113.7, 97.4, 56.7, 56.4, 56.1, 22.6 ppm.

IR (ν_{max}/cm^{-1}, neat): 2998–2834, 1513, 1464, 1439, 1397, 1319, 1215, 1206, 1127, 1080, 1035 cm^{-1}.

HRMS (ESI$^+$) calcd. for $C_{12}H_{15}O_3ClNa$ $[M+Na]^+$ 265.0602, found 265.0605.

---

[17] S. Nerdinger, C. Kendall, X. Cai, R. Marchart, P. Riebel, M. R. Johnson, C.-F. Yin, N. Hénaff, L. D. Eltis, V. Snieckus, *J. Org. Chem.* **2007**, *72*, 5960 – 5967.

*Synthesis of model boronic esters **S15** and **S16***

5,5-Dimethyl-2-(2-methyl-1-(2,4,5-trimethoxyphenyl)propyl)-1,3,2-dioxaborinane (S15)

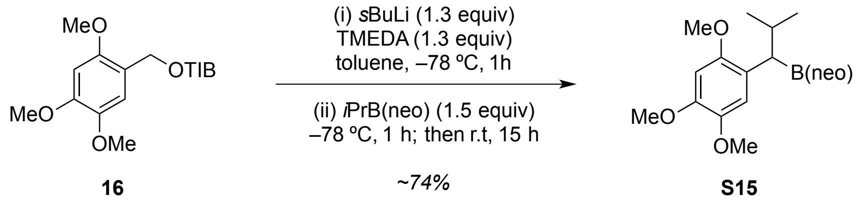

To a solution of benzylic TIB-ester **16** (484 mg, 1.13 mmol, 1.00 equiv) and TMEDA (219 µL, 1.47 mmol, 1.30 equiv) in anhydrous toluene (5.7 mL) at –78 ºC was added *s*BuLi (1.3 M, 1.13 mL, 1.47 mmol, 1.30 equiv) dropwise over 2 min. The reaction was stirred at –78 ºC for 1 h before the addition of a solution of *i*PrB(neo) (265 mg, 1.70 mmol, 1.50 equiv) dropwise over 1 min. The mixture was stirred for 2 h at –78 ºC before the cooling bath was removed and stirring was continued at room temperature for 15 h. After this time, the solvent was removed under reduced pressure and the residue was re-dissolved in anhydrous Et_2O (3.0 mL) and filtered through a plug of silica, eluting with Et_2O, before concentrating *in vacuo* to give crude boronic ester **S15** (365 mg) as a colourless oil. ^{1}H NMR analysis showed a 1.00:0.40 ratio of **S15**:TIBOH, which equates to a yield of approximately 282 mg, 74%.

Neopentyl glycol boronic ester **S15** was unstable to FCC and was found to decompose upon standing at ambient temperature. The product was, therefore, used without further purification.

^{1}H NMR (400 MHz, $CDCl_3$) δ_H 6.83 (s, 1H), 6.52 (s, 1H), 3.87 (s, 3H), 3.83 (s, 3H), 3.78 (s, 3H), 3.59 (s, 4H), 2.26 (d, J = 10.0 Hz, 1H), 2.07–1.98 (m, 1H), 1.04 (d, J = 6.6 Hz, 3H), 0.91 (s, 6H), 0.70 (d, J = 6.6 Hz, 3H) ppm.

4,4,5,5-Tetramethyl-2-(2-methyl-1-(2,4,5-trimethoxyphenyl)propyl)-1,3,2-dioxaborolane (S16)

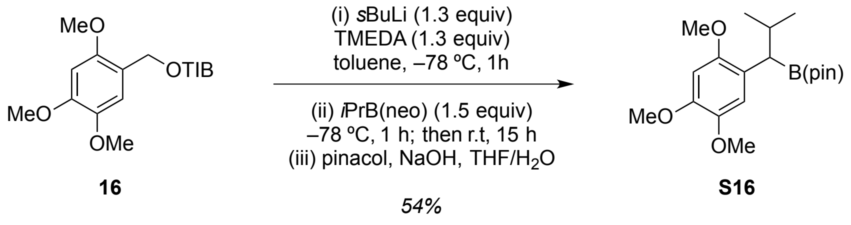

To a solution of benzylic TIB-ester **16** (1.29 g, 3.00 mmol, 1.00 equiv) and TMEDA (581 µL, 3.90 mmol, 1.30 equiv) in anhydrous toluene (15 mL) at –78 ºC was added *s*BuLi (1.3 M, 3.00 mL, 3.90 mmol, 1.30 equiv) dropwise over 2 min. The reaction was stirred at –78 ºC for 1 h before the addition of a solution of *i*PrB(neo) (702 mg, 4.50 mmol, 1.50 equiv) in anhydrous toluene (2.0 mL) dropwise over 1 min. The mixture was stirred for 2 h at –78 ºC before the cooling bath was removed and stirring was continued at room temperature for 15 h. After this time, the solvent was removed under reduced pressure and the residue was re-dissolved in THF (10 mL). Pinacol (1.06 g, 9.00 mmol, 3.00 equiv) and NaOH (0.2 M in water, 3.0 mL) were added subsequently and the reaction mixture was stirred for 1 h at room temperature. Water (15 mL) and Et_2O (15 mL) were added, the phases were separated, and the aqueous phase was extracted with Et_2O (2 × 15 mL). The combined organic phases were dried ($MgSO_4$), filtered and the solvent was removed *in vacuo*. Purification by FCC (SiO_2, 10%–20% EtOAc/pentane) gave the title compound (569 mg, 1.62 mmol, 54%) as a colourless oil.

R$_f$: 0.33 (20% EtOAc/pentane).

^{1}H NMR (400 MHz, $CDCl_3$) δ_H 6.83 (s, 1H), 6.50 (s, 1H), 3.87 (s, 3H), 3.83 (s, 3H), 3.77 (s, 3H), 2.40 (d, *J* = 9.8 Hz, 1H), 2.04 (dhept, *J* = 9.8, 6.5 Hz, 1H), 1.23 (s, 6H), 1.20 (s, 6H), 1.03 (d, *J* = 6.6 Hz, 3H), 0.73 (d, *J* = 6.6 Hz, 3H) ppm.

^{13}C NMR (126 MHz, $CDCl_3$) δ_C 151.7, 146.9, 142.8, 122.7, 114.0, 98.1, 82.9, 56.8, 56.4, 56.0, 32.5, 30.6, 24.8, 24.6, 23.0, 21.8 ppm.

^{11}B NMR (96 MHz, $CDCl_3$) δ_B 31.6 (br s) ppm.

IR (ν_{max}/cm^{-1}, neat): 2951, 1515, 1316, 1205, 1140, 1035.

HRMS (ESI$^+$) calcd. for $C_{19}H_{31}O_4BNa$ $[M+Na]^+$ 373.2160, found 373.2161.

*Attempted Zweifel reaction between vinyl iodide **4** and model boronic ester **S16***

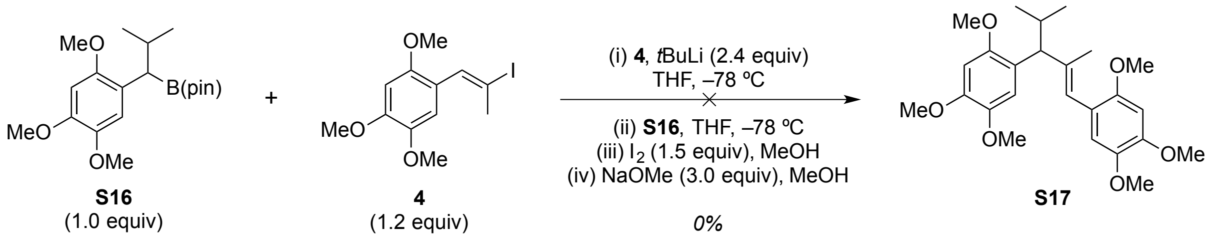

To a solution of vinyl iodide **4** (126 mg, 0.376 mmol, 1.20 equiv) in anhydrous THF (2.0 mL) at –78 ºC was added *t*BuLi (1.7 M in pentane, 0.44 mL, 0.75 mmol, 2.4 equiv) dropwise. The mixture was stirred at –78 ºC for 30 min before the dropwise addition of a solution of boronic ester **S16** (114 mg, 0.313 mmol, 1.00 equiv) in anhydrous THF (1.0 mL). The mixture was stirred at the same temperature for 45 min before the addition of a solution of iodine (119 mg, 0.470 mmol, 1.5 equiv) in MeOH (2.0 mL). The mixture was stirred for 15 min at –78 ºC before the addition of a solution of sodium methoxide (51 mg, 0.94 mmol, 3.0 equiv) in MeOH (1.0 mL). The resulting mixture was allowed to room temperature and stirred for 60 min before removal of the solvents *in vacuo*. The residue was dissolved in Et_2O (10 mL) and washed with saturated aqueous $Na_2S_2O_3$. The aqueous layer was extracted with Et_2O (2 × 5 mL) and the combined organic phases washed with brine (10 mL), dried ($MgSO_4$), filtered and concentrated *in vacuo*. ^{1}H NMR and GCMS analysis of the crude product showed only unreacted starting materials **4** and **S16**.

The fact that vinyl iodide **4** was returned, despite complete consumption (TLC) after treatment with *t*BuLi and complete formation of boronate complex **S18** (^{11}B NMR), suggests that the presence of the electron-rich trimethoxyphenyl group promotes regioselective opening of the intermediate iodonium **S19** to form stabilized carbocation **S20**, which undergoes elimination to return the starting materials (see Scheme below). This pathway dominates over the desired 1,2-metallate rearrangement to give β-iodoboronic ester **S21**.

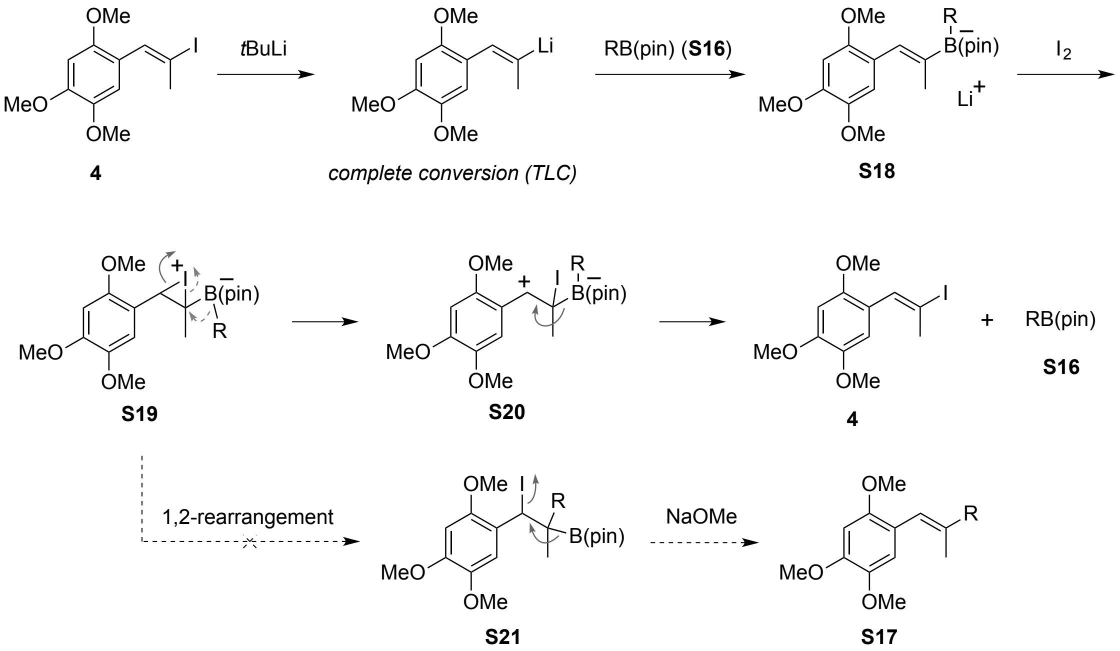

<u>*Attempted Suzuki reaction between vinyl iodide **4** and model boronic ester **S15***</u>

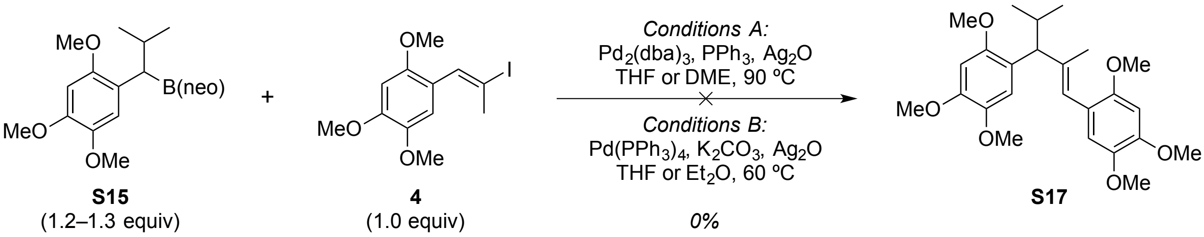

Table S3:

Entry[a]	conditions	solvent	% yield[b]
1	A	THF	0
2	A	DME	0
3	B	Et_2O	0
4	B	THF	0

[a] Reactions were performed using general procedures outlined below. [b] Determined by ^{1}H NMR analysis of the crude product. DME = 1,2-dimethoxyethane.

Conditions A for Table S3, entries 1 and 2: Following a modified literature procedure.[18] A vial was charged with $Pd(dba)_2$ (2.9 mg, 5.0 µmol, 5.0 mol%), PPh_3 (2.6 mg, 10 µmol, 10 mol%) and Ag_2O (35 mg, 0.15 mmol, 1.5 equiv) before evacuating and backfilling with N_2 three times. A solution of vinyl iodide **4** (33 mg, 0.10 mmol, 1.0 equiv) and boronic ester **S15** (40 mg, 0.12 mmol, 1.2 equiv) in anhydrous THF (1.0 mL) or DME (1.0 mL) was added and the vial was sealed. The reaction was stirred and heated at 90 ºC for 16 h before allowing to cool to room temperature and filtering through a plug of silica, eluting with EtOAc, and concentrating *in vacuo*. The crude mixture was analyzed by ^{1}H NMR. No desired product **S17** was observed.

[18] D. Imao, B. W. Glasspoole, V. S. Laberge, C. M. Crudden, *J. Am. Chem. Soc.* **2009**, *131*, 5024 – 5025.

Conditions B for Table S3, entries 3 and 4: Following a modified literature procedure.[13a] A vial was charged with $Pd(PPh_3)_4$ (9.2 mg, 8.0 µmol, 8.0 mol%), K_2CO_3 (22 mg, 0.16 mmol, 1.6 equiv) and Ag_2O (37 mg, 0.16 mmol, 1.6 equiv) before evacuating and backfilling with N_2 three times. A solution of vinyl iodide **4** (33 mg, 0.10 mmol, 1.0 equiv) and boronic ester **S15** (44 mg, 0.13 mmol, 1.3 equiv) in anhydrous Et_2O (2.0 mL) or THF (2.0 mL) was added and the vial was sealed. The reaction was stirred and heated at 60 ºC for 60 h before allowing to cool to room temperature and filtering through celite, eluting with Et_2O, and concentrating *in vacuo*. The crude mixture was analyzed by ^{1}H NMR. No desired product **S17** was observed.

*Attempted stereospecific Suzuki reaction with potassium trifluoroborate **S22***

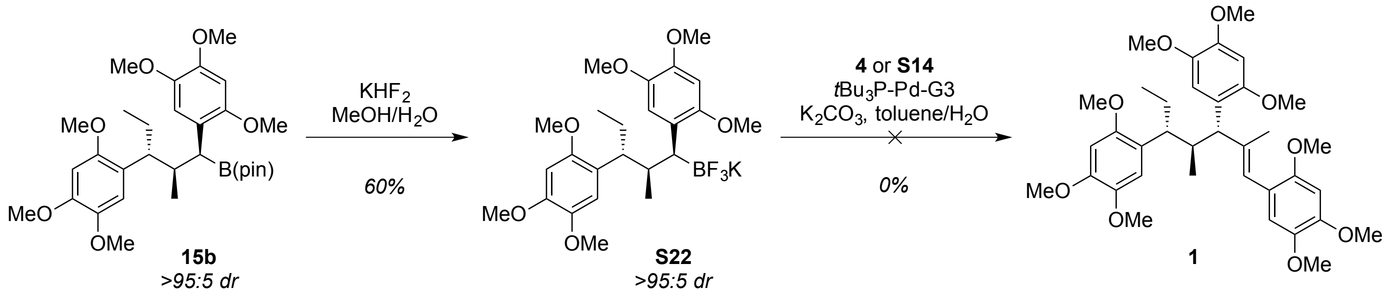

Potassium ((1*S*,2*R*,3*R*)-2-methyl-1,3-bis(2,4,5-trimethoxyphenyl)pentyl)trifluoroborate (S22)

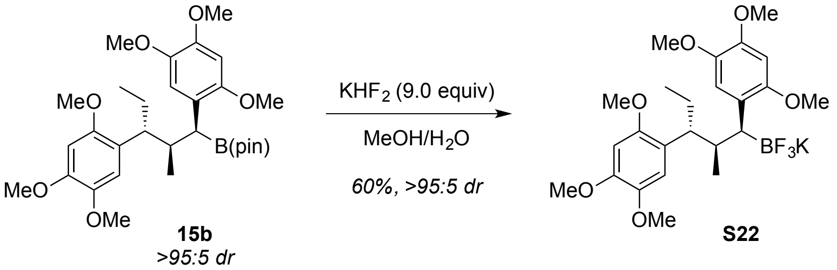

To a solution of pinacol boronic ester **15b** (79 mg, 0.15 mmol, 1.0 equiv) in MeOH (0.57 mL) at room temperature was added a solution of potassium hydrogen fluoride (102 mg, 1.31 mmol, 9.0 equiv) in H_2O (0.29 mL) dropwise over 30 seconds. The resulting white suspension was stirred at room temperature for 45 min before removal of the solvents under reduced pressure. The solid was extracted with refluxing acetone (4 × 5 mL) and filtered through cotton wool before concentrating *in vacuo*. The resulting white solid was triturated with Et_2O from hot acetone to give the title compound (47 mg, 0.087 mmol, 60%) as a white solid.

Mpt (racemate): 217–219 ºC (acetone/Et_2O)

^{1}**H NMR** (500 MHz, DMSO-*d*$_6$) δ_H 7.01 (s, 1H), 6.52 (br s, 1H), 6.51 (s, 1H), 6.49 (s, 1H), 3.73 (s, 3H), 3.71 (s, 3H), 3.68 (s, 3H), 3.64 (s, 3H), 3.59 (s, 3H), 3.49 (s, 3H), 2.49–2.44 (m, 1H), 2.37–2.29 (m, 1H), 2.20 (dqd, *J* = 13.1, 7.4, 3.1 Hz, 1H), 2.02–1.96 (m, 1H), 1.32–1.23 (m, 1H), 0.50 (t, *J* = 7.2 Hz, 2H), 0.49 (d, *J* = 6.7 Hz, 3H) ppm.

^{13}**C NMR** (126 MHz, DMSO-*d*$_6$) δ_C 152.8, 152.3, 146.4, 145.3, 142.9, 141.5, 128.2, 127.1, 118.6, 112.7 (br), 99.1, 98.6, 56.7, 56.6, 56.4, 56.4, 55.8, 55.6, 38.3, 28.6 (br), 25.9, 17.2, 12.4 ppm.

^{11}**B NMR** (96 MHz, DMSO-*d*$_6$) δ_B 3.02 ppm.

^{19}**F NMR** (283 MHz, DMSO-d_6) δ_F −136.8 (3F, s) ppm.

IR (ν_{max}/cm^{-1}, neat): 2982–2833, 1509, 1466, 1455, 1449, 1441, 1399, 1221, 1202, 1183, 1080, 1031, 993, 980, 932.

m/z (ESI$^+$) 529.2 ([M–F$_3$+(OMe)$_2$]$^+$, 100%), 513.3 ([M–F$_3$K+Na(OMe)$_2$]$^+$, 26%).

Procedure for attempted stereoinvertive Suzuki cross-coupling:

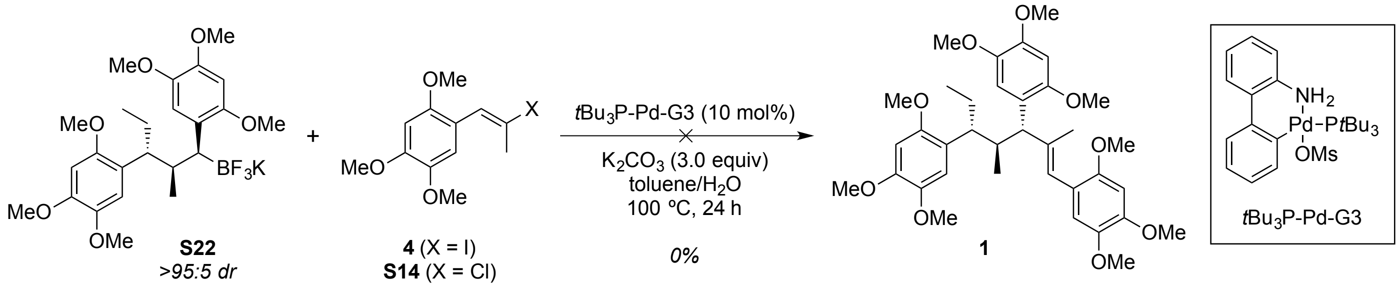

Following a modified literature procedure.[19] A vial was charged with a stirrer bar, potassium trifluoroborate **S22** (22 mg, 42 µmol, 1.0 equiv), *t*Bu-Pd-G3 (2.4 mg, 4.5 µmol, 10 mol%) and K_2CO_3 (17 mg, 0.13 mmol, 3.0 equiv). The vial was placed under vacuum and backfilled with N_2 four times before the addition of a solution of vinyl chloride **S14** (10 mg, 42 µmol, 1.0 equiv) [or vinyl iodide **4** (14 mg, 42 µmol, 1.0 equiv)] in degassed toluene (168 µL), followed by degassed H_2O (84 µL). The vial was sealed with a screw-top cap secured with Teflon tape before being heating to 100 ºC and stirred for 24 h. After allowing to cool to ambient temperature the mixture was diluted with EtOAc (2 mL) and filtered through a plug of silica, eluting with further EtOAc (3 × 2 mL), and concentrating *in vacuo*.

Analysis of the crude product by ^{1}H NMR showed that no tatanan A (**1**) was formed. The main side-product was the boronic acid formed by hydrolysis of the potassium trifluoroborate group of **S22** (data not shown).

[19] L. Li, S. Zhao, A. Joshi-Pangu, M. Diane, M. R. Biscoe, *J. Am. Chem. Soc.* **2014**, *136*, 14027 – 14030.

8. Synthesis of 3-*epi*-Tatanan A

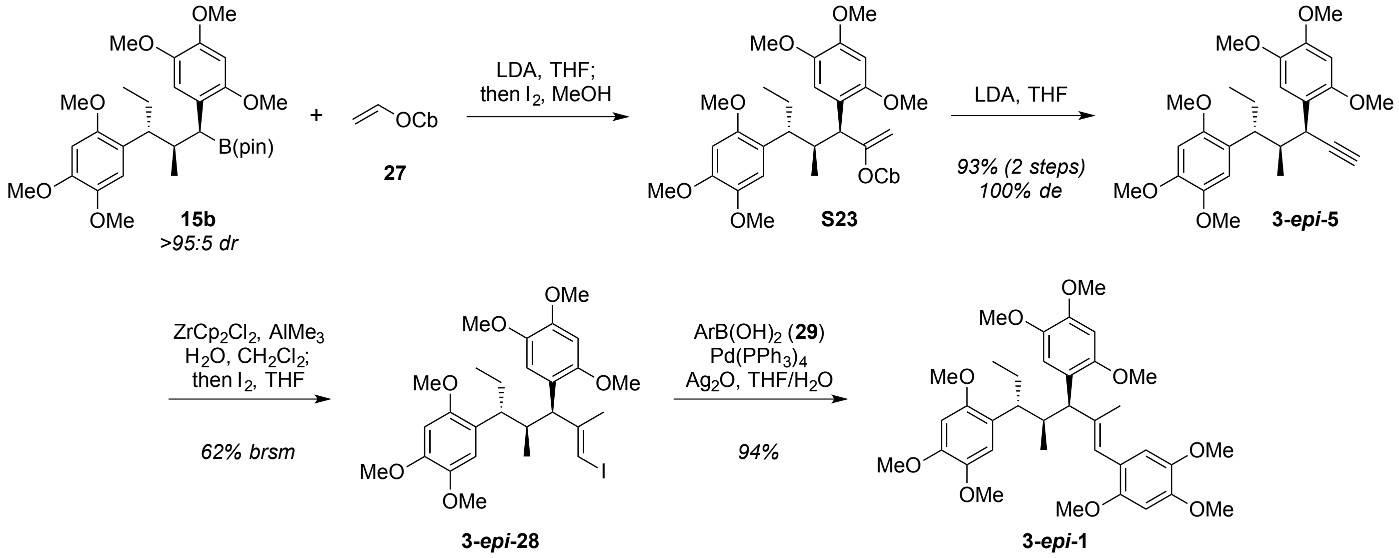

5,5'-((3*R*,4*S*,5*R*)-4-Methylhept-1-yne-3,5-diyl)bis(1,2,4-trimethoxybenzene) (3-*epi*-5)

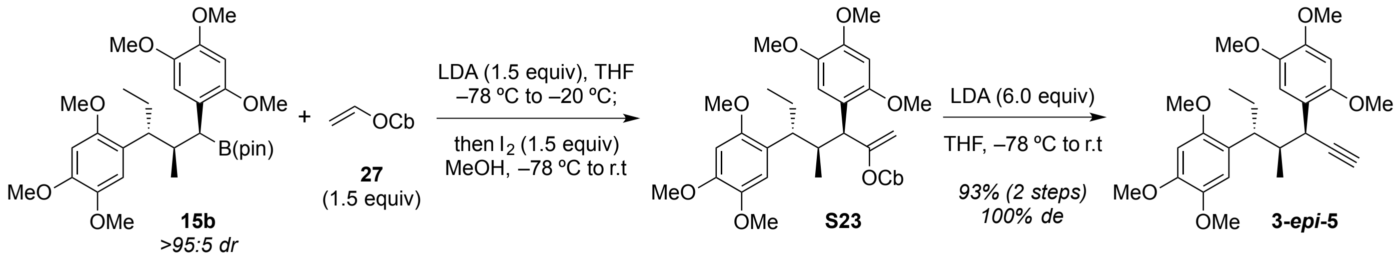

Following a modified literature procedure.[20] To a solution of boronic ester **15b** (61 mg, 0.11 mmol, 1.0 equiv) and vinyl carbamate **27** (29 mg, 0.17 mmol, 1.5 equiv) in anhydrous THF (1.1 mL) at –78 ºC was added LDA (0.86 M in THF, 0.20 mL, 0.17 mmol, 1.5 equiv) dropwise over 3 min. The reaction was stirred at –78 ºC for 60 min before the reaction vessel was transferred to a –40 ºC bath and stirred for 30 min. The reaction was cooled to –78 ºC and a solution of I_2 (43 mg, 0.17 mmol, 1.5 equiv) in MeOH (1.1 mL) was added dropwise over 5 min. The mixture was stirred at –78 ºC for 10 min before removing from the cold bath and stirring at room temperature for 60 min. Saturated aqueous $Na_2S_2O_3$ (5 mL) was added and the product extracted into Et_2O (3 × 5 mL). The combined organic extracts were washed with water (5 mL), brine (5 mL), dried ($MgSO_4$), filtered and concentrated *in vacuo*. The resulting crude vinyl carbamate **S23** was further dried under high-vacuum for 30 min before placing under an atmosphere of N_2, dissolving in anhydrous THF (1.1 mL) and cooling to –78 ºC. LDA (0.86 M in THF, 0.78 mL, 0.67 mmol, 6.0 equiv) was added dropwise over 1 min and the resulting solution was removed from the cold bath and stirred at room temperature for 15 min. The reaction was quenched by the addition of saturated aqueous NH_4Cl (5 mL) and the product extracted into Et_2O (3 × 5 mL). The combined organic extracts were washed with brine (5 mL), dried ($MgSO_4$), filtered and concentrated *in vacuo*. The residue was purified by FCC (SiO_2, 25% EtOAc/pet. ether) to give the title compound (46 mg, 0.10 mmol, 93%) as a colourless oil. The reaction was determined by ^1H NMR analysis to proceed with 100% diastereomeric excess (de).

R$_f$: 0.29 (25% EtOAc/pet. ether).

[20] Y. Wang, A. Noble, E. L. Myers, V. K. Aggarwal, *Angew. Chem. Int. Ed.* **2016**, *55*, 4270 – 4274.

^{1}H NMR (500 MHz, $CDCl_3$) δ_H 7.00 (s, 1H), 6.65 (s, 1H), 6.51 (s, 1H), 6.48 (s, 1H), 4.04 (dd, J = 7.3, 2.5 Hz, 1H), 3.89 (s, 3H), 3.85 (s, 3H), 3.82 (s, 3H), 3.72 (s, 3H), 3.72 (s, 3H), 3.19 (br s, 1H), 2.25 – 2.15 (m, 1H), 2.20 (d, J = 2.5 Hz, 1H), 1.89 (dqd, J = 13.2, 7.4, 4.9 Hz, 1H), 1.71 – 1.62 (m, 1H), 0.76 (t, J = 7.3 Hz, 3H), 0.57 (d, J = 7.0 Hz, 3H) ppm.

^{13}C NMR (126 MHz, $CDCl_3$) δ_C 152.8, 151.1, 148.3, 147.4, 143.0, 142.8, 123.2, 120.1, 113.7, 113.5, 98.0, 97.3, 88.1, 69.1, 56.7, 56.7, 56.6, 56.4, 56.1, 56.0, 44.0, 42.0 (br), 33.7, 26.7, 14.3, 12.6 ppm.

IR (ν_{max}/cm^{-1}, neat): 3286, 2960–2832, 1508, 1463, 1439, 1397, 1315, 1202, 1179, 1034.

HRMS (ESI$^+$) calcd. for $C_{26}H_{34}O_6Na$ $[M+Na]^+$ 465.2248, found 465.2244.

$[\alpha]_D^{24}$ –28.3 (c 1.00, $CHCl_3$).

5,5'-((3*S*,4*S*,5*R*,*E*)-1-Iodo-2,4-dimethylhept-1-ene-3,5-diyl)bis(1,2,4-trimethoxybenzene) (3-*epi*-28)

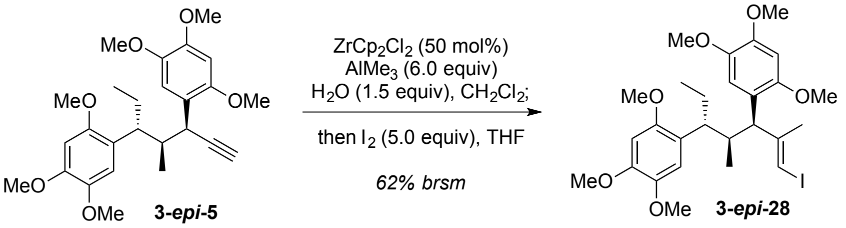

Following a modified literature procedure.[21] To a solution of zirconocene dichloride (19 mg, 0.067 mmol, 0.50 equiv) in anhydrous CH_2Cl_2 (1.3 mL) at 0 ºC was added trimethylaluminium (2 M in toluene, 0.40 mL, 0.80 mmol, 6.0 equiv) followed by H_2O (3.6 µL, 0.20 mmol, 1.5 equiv). The mixture was allowed to warm to room temperature and stirred for 30 min to give a yellow solution, which was subsequently cooled to 0 ºC. A solution of alkyne **3-*epi*-5** (59 mg, 0.13 mmol, 1.0 equiv) in anhydrous CH_2Cl_2 (1.4 mL) was added dropwise over 1 min. The ice bath was removed and the reaction was stirred at room temperature for 16 h before cooling back to 0 ºC. A solution of I_2 (170 mg, 0.67 mmol, 5.0 equiv) in THF (0.7 mL) was added via cannula and the resulting mixture stirred at 0 ºC for 20 min before allowing to warm to room temperature and stirring for a further 60 min. Saturated aqueous $Na_2S_2O_3$ (5 mL) was added and the phases separated. The aqueous phase was extracted with CH_2Cl_2 (3 × 5 mL) and the combined organic extracts were dried ($MgSO_4$), filtered, and concentrated *in vacuo*. The residue was purified by FCC (SiO_2, 2% EtOAc/CH_2Cl_2) to give the title compound (37 mg, 0.063 mmol, 48%, 62% brsm) as a colourless oil, as well as recovered alkyne **3-*epi*-5** (14 mg, 0.032 mmol, 24%).

R_f: 0.45 (2% EtOAc/CH_2Cl_2).

^{1}H NMR (500 MHz, $CDCl_3$) δ_H 6.68 (s, 1H), 6.62 (s, 1H), 6.52 (s, 1H), 6.45 (s, 1H), 6.09 (s, 1H), 3.91 (s, 3H), 3.86 (s, 3H), 3.83 (s, 3H), 3.81 (s, 3H), 3.75 (s, 3H), 3.73 (d, J = 10.1 Hz, 1H), 3.65 (s, 3H), 3.19 (br s, 1H), 2.38 (dqd, J = 10.1, 7.0, 4.5 Hz, 1H), 1.74 – 1.68 (m, 2H), 1.72 (s, 3H), 0.79 (t, J = 7.3 Hz, 3H), 0.60 (d, J = 6.9 Hz, 3H) ppm.

^{13}C NMR (126 MHz, $CDCl_3$) δ_C 152.9, 152.0, 149.3, 147.9, 147.4, 143.0, 142.5, 122.7, 121.7, 113.8, 112.9, 98.0, 97.5, 76.1, 56.84, 56.77, 56.67, 56.67, 56.00, 55.99, 49.2, 40.1 (br), 38.6, 26.8, 24.5, 14.3, 12.7 ppm.

IR (ν_{max}/cm^{-1}, neat): 2994–2831, 1609, 1508, 1463, 1396, 1315, 1204, 1179, 1036.

[21] G. Jürjens, A. Kirschning, *Org. Lett.* **2014**, *16*, 3000 – 3003.

HRMS (ESI^+) calcd. for $C_{27}H_{37}IO_4Na$ $[M+Na]^+$ 607.1527, found 607.1516.

$[\alpha]_D^{24}$ +32.0 (*c* 0.77, $CHCl_3$).

5,5',5''-((3*R*,4*S*,5*R*,*E*)-2,4-Dimethylhept-1-ene-1,3,5-triyl)tris(1,2,4-trimethoxybenzene) (3-*epi*-1)

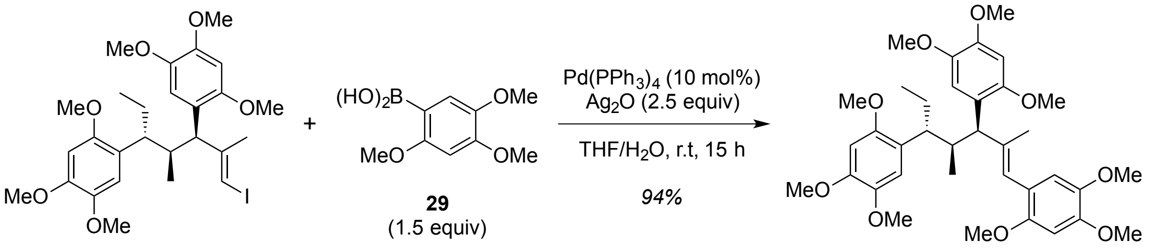

A vial was charged with vinyl iodide **3-*epi*-28** (17 mg, 0.029 mmol, 1.0 equiv), 2,4,5-trimethoxyphenylboronic acid (**29**) (9.2 mg, 0.044 mmol, 1.5 equiv), $Pd(PPh_3)_4$ (3.4 mg, 2.9 µmol, 10 mol%), and Ag_2O (17 mg, 0.073 mmol, 2.5 equiv). The vial was sealed with a septum and triple evacuated/N_2 filled before the addition of degassed (sparged with N_2 for 10 min) THF/H_2O (5:1, 0.58 mL). The mixture was stirred at room temperature for 15 h before filtering through a plug of silica, eluting with EtOAc, and concentrating *in vacuo*. The residue was purified by preparative TLC (SiO_2, 40% EtOAc/pet. ether) to give the title compound (17 mg, 0.027 mmol, 94%) as a colourless oil.

R_f: 0.40 (40% EtOAc/pet. ether).

^{1}H NMR (500 MHz, $CDCl_3$) δ_H 6.87 (s, 1H), 6.78 (s, 1H), 6.75 (s, 1H), 6.59 (s, 1H), 6.55 (s, 1H), 6.54 (s, 1H), 6.46 (s, 1H), 3.91 (s, 6H), 3.87 (s, 3H), 3.83 (s, 3H), 3.82 (s, 3H), 3.81 (s, 3H), 3.80 (s, 3H), 3.72 (s, 3H), 3.66 (s, 3H), 3.68 – 3.62 (m, 1H), 3.54 – 3.39 (m, 1H), 2.52 – 2.43 (m, 1H), 1.85 – 1.76 (m, 2H), 1.64 (s, 3H), 0.82 (t, J = 7.3 Hz, 3H), 0.59 (d, J = 6.9 Hz, 3H) ppm.

^{1}H NMR (500 MHz, CD_3OD) δ_H 6.88 (s, 1H), 6.79 (s, 1H), 6.77 (s, 1H), 6.67 (s, 1H), 6.65 (s, 1H), 6.57 (s, 1H), 6.55 (s, 1H), 3.86 (s, 6H), 3.81 (s, 3H), 3.81 (s, 3H), 3.76 (s, 3H), 3.75 (s, 3H), 3.74 (s, 3H), 3.73 (s, 3H), 3.65 (s, 3H), 3.65 – 3.61 (m, 1H), 3.55 – 3.46 (m, 1H), 2.51 – 2.42 (m, 1H), 1.92 – 1.73 (m, 2H), 1.57 (s, 3H), 0.80 (t, J = 7.3 Hz, 3H), 0.59 (d, J = 6.9 Hz, 3H) ppm.

^{13}C NMR (126 MHz, $CDCl_3$) δ_C 153.1, 152.2, 151.7, 148.0, 147.30, 147.28, 143.1, 142.6, 142.5, 140.2, 123.9, 123.5, 120.3, 119.0, 114.7, 114.1, 112.8, 98.02, 97.97, 97.9, 57.0, 56.76, 56.76, 56.70, 56.68, 56.4, 56.2, 56.00, 55.96, 48.2, 41.2 (br), 39.1, 26.8, 18.9, 14.3, 12.9 ppm.

^{13}C NMR (126 MHz, CD_3OD) δ_C 153.6, 152.6, 152.2, 148.5, 147.9, 147.8, 143.0, 142.5, 142.4, 139.7, 123.9, 123.1, 120.0, 119.1, 115.34, 115.34, 113.5, 98.18, 98.16, 98.1, 56.34, 56.26, 56.0, 55.71, 55.66, 55.6, 55.4, 55.31, 55.30, 48.0, 40.8 (br), 39.1, 26.2, 17.8, 13.5, 11.9 ppm

IR (ν_{max}/cm^{-1}, neat): 2996–2832, 1508, 1464, 1455, 1439, 1396, 1315, 1203, 1178, 1036.

HRMS (ESI^+) calcd. for $C_{36}H_{48}O_9Na$ $[M+Na]^+$ 647.3191, found 647.3201.

$[\alpha]_D^{23}$ +47.6 (*c* 0.77, $CHCl_3$).

9. Synthesis of Racemic Pinacol Boronic Ester 17

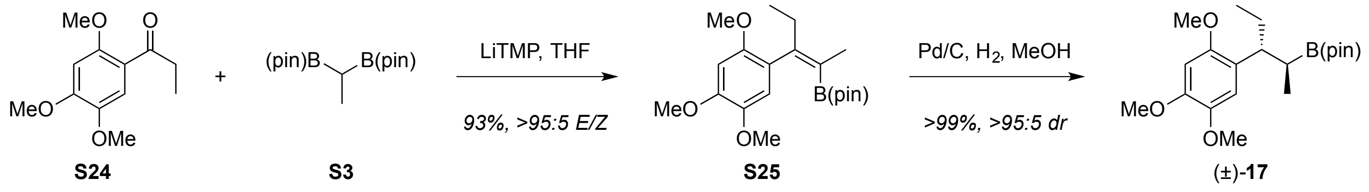

2,4,5-Trimethoxypropiophenone (Isoacoramone) (S24)

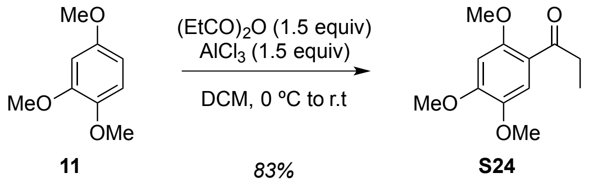

To a stirred solution of 1,4,5-trimethoxybenzene (**11**) (1.49 mL, 10.0 mmol, 1.00 equiv) and propionic anhydride (1.92 mL, 15.0 mmol, 1.50 equiv) in anhydrous CH_2Cl_2 (27 mL) at 0 ºC was added aluminium trichloride (2.20 g, 15.0 mmol, 1.50 equiv) in one portion. The reaction was removed from the ice bath and stirred at room temperature for 2 h. The reaction was quenched with 0.5 M aqueous HCl (50 mL) and the layers separated. The aqueous layer was further extracted with CH_2Cl_2 (2 × 20 mL) and the combined organic extracts were dried ($MgSO_4$), filtered, and concentrated *in vacuo.* The resulting solid was recrystallized from Et_2O/pet. ether to yield the title compound (1.86 g, 8.29 mmol, 83%) as a white solid.

Mpt: 106–108 ºC (Et_2O/pet. ether) [Lit. 108–109 ºC (MeOH)].[22]

^{1}H NMR (400 MHz, $CDCl_3$) δ_H 7.44 (s, 1H), 6.51 (s, 1H), 3.96 (s, 3H), 3.92 (s, 3H), 3.88 (s, 3H), 3.00 (q, *J* = 7.2 Hz, 2H), 1.17 (t, *J* = 7.2 Hz, 3H) ppm.

All spectroscopic data match those reported in the literature.[23]

(*E*)-4,4,5,5-Tetramethyl-2-(3-(2,4,5-trimethoxyphenyl)pent-2-en-2-yl)-1,3,2-dioxaborolane (S25)

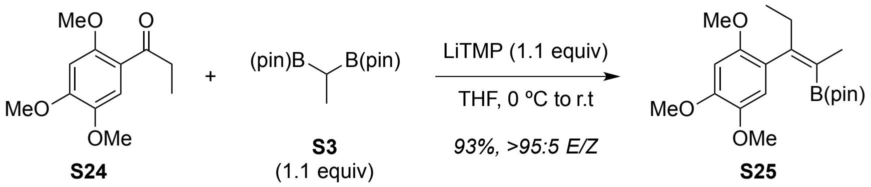

Prepared following a modified literature procedure.[24] To a solution of 1,1-bis[(pinacolato)boryl]ethane (**S3**) (274 mg, 0.972 mmol 1.10 equiv) in anhydrous THF (2.2 mL) at 0 ºC was added freshly prepared LiTMP (0.4 M in THF, 2.43 mL, 0.972 mmol, 1.10 equiv) dropwise over 1 min. The mixture was stirred at 0 ºC for 5 min before the addition of solid 2,4,5-trimethoxypropiophenone (**S24**) (198 mg, 0.883 mmol, 1.0 equiv) in one portion. The reaction was removed from the ice bath and stirred at room temperature for 2 h before filtering through a plug of silica, eluting with Et_2O, and concentrating *in vacuo*. The residue was purified by

[22] T. Högberg, S. Bengtsson, T. de Paulis, L. Johansson, P. Ström, H. Hall, S. O. Ögren, *J. Med. Chem.* **1990**, *33*, 1155 – 1163.
[23] C. H. Park, K. H. Kim, I. K. Lee, S. Y. Lee, S. U. Choi, J. H. Lee, K. R. Lee, *Arch. Pharm. Res.* **2011**, *34*, 1289 – 1296.
[24] K. Endo, M. Hirokami, T. Shibata, *Org. Lett.* **2010**, *75*, 3469 – 3472.

FCC (SiO_2, 10–15% EtOAc/pet. ether) to yield the title compound (297 mg, 0.820 mmol, 93%) as a colourless oil that crystallized upon standing. The *E*:*Z* ratio of the product was determined by ^{1}H NMR to be >95:5.

Mpt: 79–80 ºC (EtOAc).

$\mathbf{R_f}$**:** 0.19 (10% EtOAc/pet. ether).

^{1}**H NMR** (500 MHz, $CDCl_3$) δ_H 6.58 (s, 1H), 6.50 (s, 1H), 3.89 (s, 3H), 3.82 (s, 3H), 3.77 (s, 3H), 2.42 (br s, 2H), 1.85 (s, 3H), 1.02 (br s, 12H), 0.88 (t, *J* = 7.6 Hz, 3H) ppm.

^{13}**C NMR** (126 MHz, $CDCl_3$) δ_C 151.4, 151.0, 148.1, 142.2, 126.0, 115.4, 98.2, 82.7, 56.9, 56.4, 56.3, 26.1, 24.5, 15.8, 12.2 ppm.

^{11}**B NMR** (96 MHz, $CDCl_3$) δ_B 29.4 (br s) ppm.

IR (ν_{max}/cm^{-1}, neat): 2975–2844, 1506, 1465, 1390, 1371, 1361, 1293, 1296, 1146, 1109, 1038.

HRMS (ESI$^+$) calcd. for $C_{20}H_{31}O_5BNa$ $[M+Na]^+$ 385.2160, found 385.2172.

4,4,5,5-Tetramethyl-2-((2*S**,3*R**)-3-(2,4,5-trimethoxyphenyl)pentan-2-yl)-1,3,2-dioxaborolane [(±)-17]

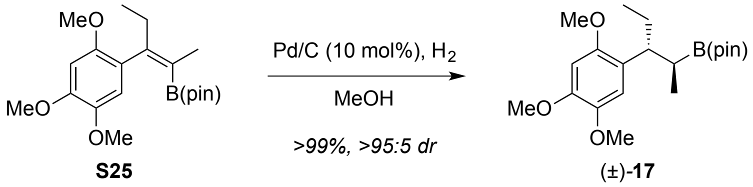

Prepared following a modified literature procedure.[25] To a 5 mL flask was added vinyl boronic ester **S25** (1.18 g, 1.25 mmol, 1.0 equiv) and MeOH (13.0 mL). The flask was purged with N_2 for 5 min before adding 10% palladium on carbon (345 mg, 0.325 mmol, 10 mol%). The flask was then purged with H_2 for 2 min before placing under a static atmosphere of H_2. The reaction was stirred vigorously for 2 h before filtering through a plug of silica, eluting with EtOAc, and concentrating *in vacuo*. Purification by FCC (SiO_2, 15% EtOAc/pet. ether) gave the title compound (1.18 g, 3.25 mmol, >99%) as a colourless oil. The diastereomeric ratio was determined by ^{1}H NMR to be >95:5.

For spectroscopic data, see the synthesis of enantioenriched **17** in Section 11.

(2*S**,3*S**)-3-(2,4,5-Trimethoxyphenyl)pentan-2-ol [(±)-S9]

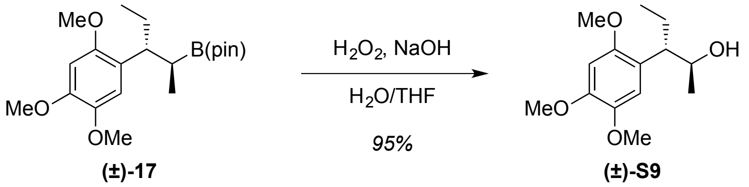

Racemic pinacol boronic ester (±)-**17** (12 mg, 0.033 mmol) in THF (2.0 mL) at 0 ºC was treated with 2.0 M aqueous NaOH (2.0 mL) and 30% aqueous H_2O_2 (1.0 mL) using the same procedure as described in Section 5 (for the oxidation of **10**) to give the title compound (8.0 mg, 0.031 mmol, 95%) as a colourless solid.

[25] E. Hupe, I. Marek, P. Knochel, *Org. Lett.* **2002**, *4*, 2861 – 2863.

10. Optimization of the Diastereoselective Matteson Homologation

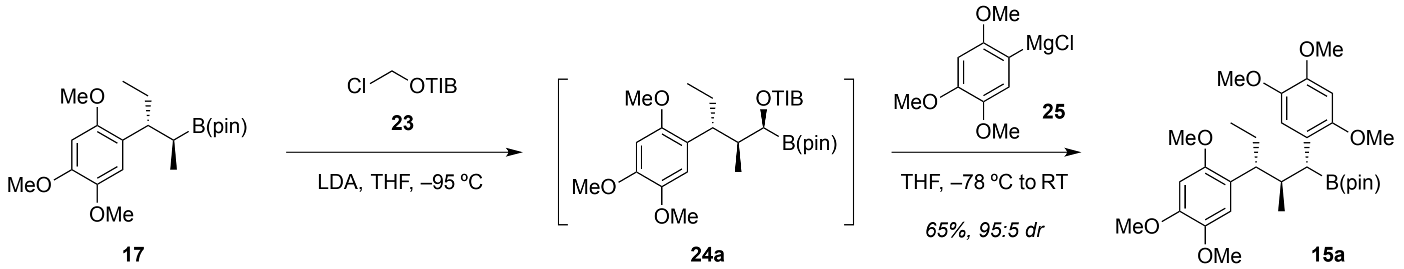

*Reaction of Boronic Esters **10** or **17** with Lithiated Chloromethyl Esters*

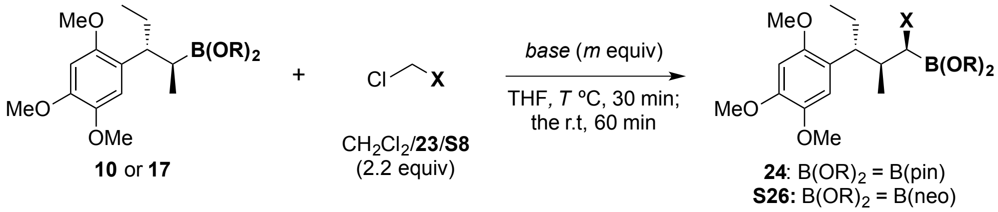

Table S4:

Entry[a]	**B(OR)$_2$**	**X**	base addition time /min	*base* (*m* equiv)	*T* ºC	% SM (**10** or **17**)[b]	% yield[b]	dr (*syn*:*anti*)[b]
1	B(pin)	OTMB (**S8**)	2	LDA (1.2)	–78	34	45	69:31
2	B(pin)	OTMB (**S8**)	2	LDA (1.5)	–78	29	52	69:31
3	B(pin)	OTMB (**S8**)	2	LDA (2.0)	–78	<5	55	77:23
4	B(pin)	OTMB (**S8**)	2	LiTMP (2.0)	–78	<5	53	75:25
5	B(pin)	OTMB (**S8**)	2	*n*BuLi (2.0)	–78	8	55	71:29
6	B(pin)	OTMB (**S8**)	30	LDA (2.0)	–78	50	17	53:47
7	B(pin)	OTMB (**S8**)	2	LDA (2.0)	–95	<5	66	70:30
8	B(pin)	OTMB (**S8**)	0.5	LDA (2.0)	–95	<5	72	69:31
9	B(pin)	Cl (CH_2Cl_2)	0.5	LDA (2.0)	–95	0	52	48:52
10	B(pin)	OTIB (**23**)	0.5	LDA (2.0)	–95	8	78	77:23
11	B(neo)	OTMB (**S8**)	0.5	LDA (2.0)	–95	5	55	89:11
12	B(neo)	TIB (**23**)	0.5	LDA (2.0)	–95	10	50	90:10

[a] Reactions performed using general procedure outlined below. [b] Determined by ^{1}H NMR using 1,3-dinitrobenzene as an internal standard. [c] Determined by ^{1}H NMR. TMB = 2,4,6-trimethylbenzoyl; TIB = 2,4,6-triisopropylbenzoyl.

General Procedure for Table S4: A solution of boronic ester **10** or **17** (0.1 mmol, 1.0 equiv) and chloromethyl ester **S8** or **23** or CH_2Cl_2 (0.22 mmol, 2.2 equiv) in anhydrous THF (1.0 mL) was cooled to temperature *T* ºC (–78 ºC or –95 ºC). LDA (0.86 M in THF, 0.12–0.20 mmol, 1.2–2.0 equiv) was added dropwise over 0.5–30 min and the resulting mixture stirred at *T* ºC for 60 min before removing from the cold bath and stirring at room temperature for 30 min. The reaction was quenched by the addition of saturated aqueous NH_4Cl (5 mL) and the phases separated. The aqueous phase was extracted with Et_2O (3 × 5 mL)

and the combined organics washed with brine (10 mL), dried ($MgSO_4$), filtered and concentrated *in vacuo*. The crude product was analyzed by ^{1}H NMR and the yield of **24** or **S26** determined using 1,3-dinitrobenzene as an internal standard. The diagnostic ^{1}H NMR signals for boronic esters **24a**, **24b**, **24c**, **S26a**, and **S26b** are as follows:

Pinacol boronic ester **24a** (X = OTIB): ^{1}H NMR (400 MHz, $CDCl_3$) δ_H 4.84 (d, *J* = 2.2 Hz, 1H, XC*H*B(pin), *syn* diastereomer), 4.58 (d, *J* = 5.1 Hz, 1H, XC*H*B(pin), *anti* diastereomer) ppm.

Pinacol boronic ester **24b** (X = OTMB): ^{1}H NMR (400 MHz, $CDCl_3$) δ_H 4.87 (d, *J* = 2.4 Hz, 1H, XC*H*B(pin), *syn* diastereomer), 4.50 (d, *J* = 6.0 Hz, 1H, XC*H*B(pin), *anti* diastereomer) ppm.

Pinacol boronic ester **24c** (X = Cl): ^{1}H NMR (400 MHz, $CDCl_3$) δ_H 3.73 (d, *J* = 4.8 Hz, 1H, XC*H*B(pin), *syn* diastereomer), 3.51 (d, *J* = 6.4 Hz, 1H, XC*H*B(pin), *anti* diastereomer) ppm.

Neopentyl glycol boronic ester **S26a** (X = OTIB): ^{1}H NMR (400 MHz, $CDCl_3$) δ_H 4.76 (d, *J* = 2.2 Hz, 1H, XC*H*B(pin), *syn* diastereomer), 4.38 (d, *J* = 5.7 Hz, 1H, XC*H*B(pin), *anti* diastereomer) ppm.

Neopentyl glycol boronic ester **S26b** (X = OTMB): ^{1}H NMR (400 MHz, $CDCl_3$) δ_H 4.80 (d, *J* = 2.4 Hz, 1H, XC*H*B(pin), *syn* diastereomer), 4.30 (d, *J* = 6.2 Hz, 1H, XC*H*B(pin), *anti* diastereomer) ppm.

*Sequential Matteson Homologation/Grignard Addition of Boronic Ester **17***

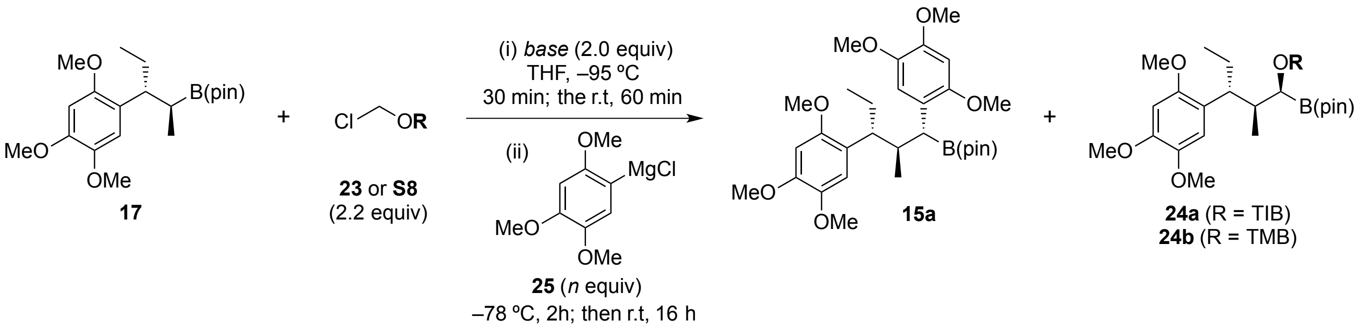

Table S5:

Entry[a]	R	*base*	*n* equiv	% yield of **24**[b]	dr (*syn*:*anti*) of **24**[c]	% yield of **15a**[b]	dr (*anti*:*syn*) of **15a**[c]
1	TMB (**S8**)	LDA	1.8	8	>98:2	65	63:37
2[d]	TMB (**S8**)	LDA	1.8	10	>98:2	68	63:37
3	TMB (**S8**)	LiTMP	2.0	31	>98:2	53	57:43
4	TMB (**S8**)	LDA	2.0	5	>98:2	63	73:27
5	TIB (**25**)	LDA	2.0	16	>98:2	49	88:12
6	TIB (**25**)	LDA	2.2	23	>98:2	54	80:20
7[e]	TIB (**25**)	LDA	2.0	19	21:79	64 (65)	95:5

[a] Reactions performed using general procedure outlined below. [b] Determined by ^{1}H NMR using 1,3-dinitrobenzene as an internal standard. Number in parenthesis is the yield of isolated product after purification by FCC. [c] Determined by ^{1}H NMR. [d] The solution of crude boronic ester **24** was added to a solution of Grignard **25** at –78 ºC. [e] An aqueous work-up was performed before the reaction of crude **24** with Grignard **25**. TMB = 2,4,6-trimethylbenzoyl; TIB = 2,4,6-triisopropylbenzoyl.

General Procedure for Table S5: A solution of boronic ester **17** (38 mg, 0.10 mmol, 1.0 equiv) and chloromethyl TMB-ester **S8** (47 mg, 0.22 mmol, 2.2 equiv), or chloromethyl TIB-ester **23** (65 mg, 0.22 mmol, 2.2 equiv), in anhydrous THF (1.0 mL) was cooled to –95 ºC before the addition of LDA (0.86 M in THF, 0.23 mL, 0.20 mmol, 2.0 equiv) dropwise over 30 s. The resulting mixture was stirred at –95 ºC for 60 min before removing from the cold bath and stirring at room temperature for 30 min. The mixture was then cooled to –78 ºC before the addition of aryl Grignard **25** (0.4 M in THF, 0.45–0.55 mL, 0.18–0.22 mmol, 1.8–2.2 equiv) dropwise over 90 s. The reaction was stirred at –78 ºC for 2 h before removing from the cold bath and stirring at room temperature for 16 h. The reaction was quenched by the addition of saturated aqueous NH_4Cl (5 mL) and the phases separated. The aqueous phase was extracted with Et_2O (3 × 5 mL) and the combined organics washed with brine (10 mL), dried ($MgSO_4$), filtered and concentrated *in vacuo*. The crude product was analyzed by ^{1}H NMR and the yields of **15a** and **24** determined using 1,3-dinitrobenzene as an internal standard.

Procedure for the synthesis of aryl Grignard ***25*** *(0.4 M in THF)*

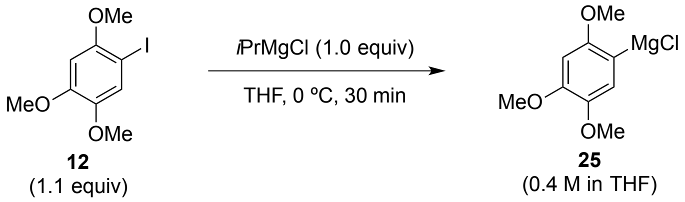

To a solution of 1-iodo-2,4,5-trimethoxylbenzene (**12**) (159 mg, 0.541 mmol, 1.10 equiv) in anhydrous THF (0.82 mL) at 0 ºC was added isopropylmagnesium chloride (2.0 M in THF, 0.25 mL, 0.49 mmol, 1.0 equiv) dropwise over 1 min. The resulting mixture was stirred at 0 ºC for 30 min to give an approximately 0.40 M solution of (2,4,5-trimethoxyphenyl)magnesium chloride (**25**), which was used immediately.

11. Synthesis of Tatanan A

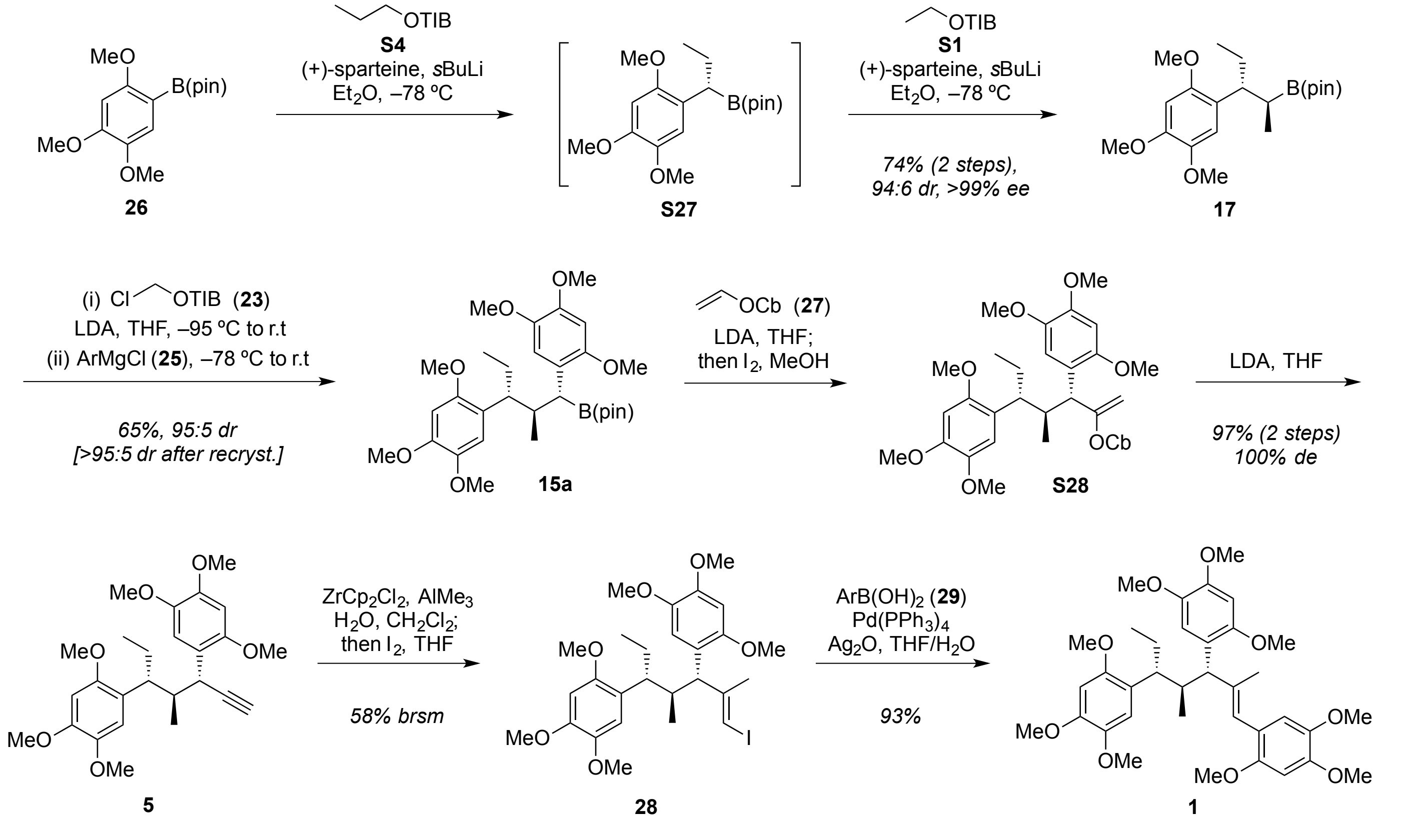

4,4,5,5-Tetramethyl-2-((2*S*,3*R*)-3-(2,4,5-trimethoxyphenyl)pentan-2-yl)-1,3,2-dioxaborolane (17)

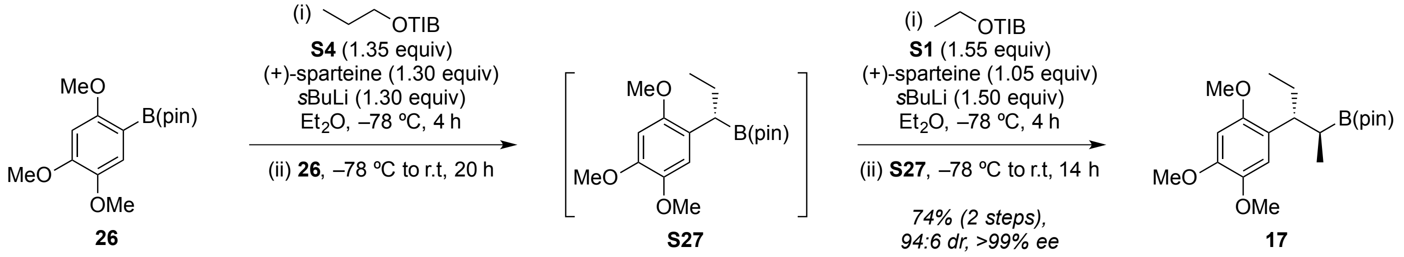

An oven-dried Schlenk tube was charged with propyl 2,4,6-triisopropylbenzoate (**S4**) (784 mg, 2.70 mmol, 1.35 equiv) before being evacuated and refilled with nitrogen three times. (+)-Sparteine (0.597 mL, 2.60 mmol, 1.30 equiv) and anhydrous Et_2O (10 mL) were then added to the Schlenk tube. The resulting solution was cooled to –78 °C before the addition of *s*BuLi (1.3 M in cyclohexane/hexanes, 2.00 mL, 2.60 mmol, 1.30 equiv) dropwise over 10 min (colour change: colourless to dark brown). The reaction mixture was stirred at –78 °C for 4 h before the addition of a solution of aryl pinacol boronic ester **26** (588 mg, 2.00 mmol, 1.00 equiv) in anhydrous Et_2O (6.6 mL) dropwise over 30 min. The resulting mixture was stirred at –78 ºC for 2 h before removing from the cold bath and stirring at room temperature for 20 h.

A separate oven-dried Schlenk tube was charged with ethyl 2,4,6-triisopropylbenzoate (**S1**) (857 mg, 3.10 mmol, 1.55 equiv) before being evacuated and refilled with nitrogen three times. (+)-Sparteine (0.689 mL, 3.00 mmol, 1.50 equiv) and anhydrous Et_2O (10 mL) were then added to the Schlenk tube. The resulting solution was cooled to –78 °C before the addition of *s*BuLi (1.3 M in cyclohexane/hexanes, 2.31 mL, 3.00 mmol, 1.50 equiv) dropwise over 10 min (colour change: colourless to dark brown). The reaction mixture

was stirred at –78 °C for 4 h before the addition of the contents of the first Schlenk tube (containing **S27**) dropwise via cannula over 40 min. The resulting mixture was stirred at –78 ºC for 2 h before removing from the cold bath and stirring at room temperature for 14 h. To the reaction was added 2 M aqueous HCl (20 mL) and the mixture stirred vigorously for 10 min before separation of the layers. The organic layer was washed with 2 M aqueous HCl (2 × 20 mL) then the combined aqueous washings were further extracted with Et_2O (2 × 20 mL) [the acidic aqueous washings were retained for the recovery of (+)-sparteine].[2] The combined organic layers were washed with brine (40 mL), dried ($MgSO_4$), filtered and concentrated *in vacuo* to give crude boronic ester **17**, which was determined to have a diastereomeric ratio of 91:9 by ^{1}H NMR. Purification by FCC (SiO_2, 5% Et_2O/toluene + 1% Et_3N) gave title compound (540 mg, 0.697 mmol, 74%) as a colourless oil, which crystallized upon standing. The diastereomeric ratio of the purified product was determined to be 94:6 by ^{1}H NMR. Further enhancement to >95:5 dr was possible by FCC.

Mpt: 43–46 ºC (Et_2O).

R_f: 0.18 (10% Et_2O/toluene) [minor diastereomer = 0.14 (10% Et_2O/toluene)].

^{1}H NMR (500 MHz, $CDCl_3$) δ_H 6.67 (s, 1H), 6.51 (s, 1H), 3.88 (s, 3H), 3.81 (s, 3H), 3.76 (s, 3H), 2.97 (td, *J* = 11.0, 3.8 Hz, 1H), 1.65 (dqd, *J* = 13.3, 7.4, 3.9 Hz, 1H), 1.54 (ddq, *J* = 13.3, 10.9, 7.3 Hz, 1H), 1.33 (dq, *J* = 11.2, 7.4 Hz, 1H), 1.29 (s, 6H), 1.28 (s, 6H), 0.74 (d, *J* = 7.4 Hz, 3H), 0.69 (t, *J* = 7.3 Hz, 3H) ppm.

^{13}C NMR (126 MHz, $CDCl_3$) δ_C 152.7, 147.0, 143.3, 125.1, 111.5, 98.0, 82.9, 56.9, 56.4, 56.0, 42.3, 29.1, 24.8, 24.7, 23.5, 14.3, 12.2 ppm.

^{11}B NMR (96 MHz, $CDCl_3$) δ_B 34.0 (br s) ppm.

IR (ν_{max}/cm^{-1}, neat): 2981–2835, 1508, 1456, 1397, 1378, 1317, 1204, 1175, 1144, 1037.

HRMS (ESI$^+$) calcd. for $C_{20}H_{33}BO_5Na$ $[M+Na]^+$ 387.2317, found 387.2327.

$[\alpha]_D^{22}$ –9.0 (*c* 0.50, $CHCl_3$).

Chiral HPLC: The e.r. was determined by HPLC [Daicel Chiralpak-IB (25 cm) with guard, 7:93 *i*-PrOH/hexanes, 1.0 mL/min, r.t., 210 nm, t_R (major) = 9.1 min, t_R (minor) = 10.2 min] to be 99.7:0.3.

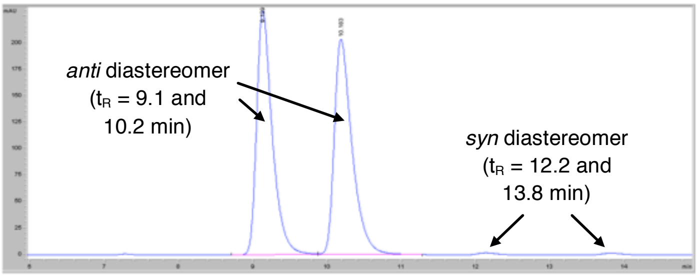

#	Time	Area	Height	Width	Area%	Symmetry
1	9.129	3390.1	230.5	0.2196	49.840	0.601
2	10.183	3411.9	203.6	0.2508	50.160	0.56

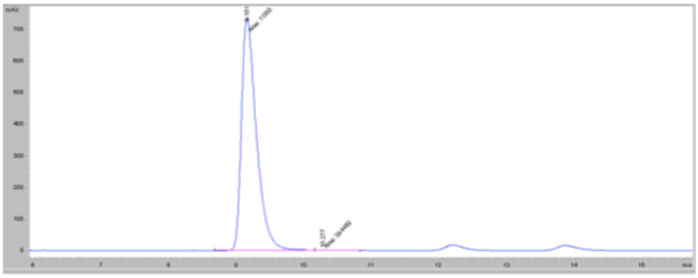

#	Time	Area	Height	Width	Area%	Symmetry
1	9.151	11353	736.3	0.257	99.689	0
2	10.277	35.4	1.8	0.3219	0.311	0.475

4,4,5,5-Tetramethyl-2-((1*R*,2*R*,3*R*)-2-methyl-1,3-bis(2,4,5-trimethoxyphenyl)pentyl)-1,3,2-dioxaborolane (15a)

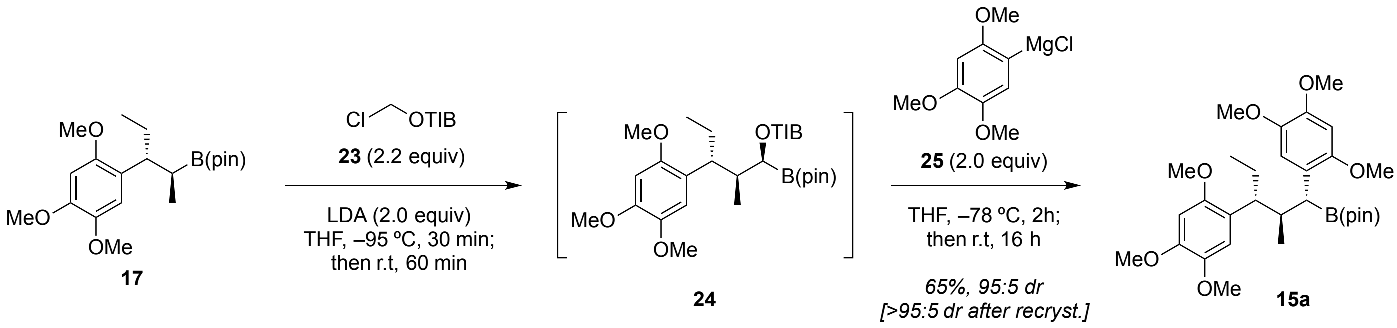

A solution of boronic ester **17** (109 mg, 0.300 mmol, 1.00 equiv) and chloromethyl TIB-ester **23** (196 mg, 0.660 mmol, 2.20 equiv) in anhydrous THF (3.0 mL) was cooled to temperature –95 ºC before the addition of LDA (0.86 M in THF, 0.70 mL, 0.60 mmol, 2.0 equiv) dropwise over 45 s. The resulting mixture was stirred at –95 ºC for 60 min before removing from the cold bath and stirring at room temperature for 30 min. The reaction was quenched by the addition of saturated aqueous NH_4Cl (15 mL) and the phases separated. The aqueous phase was extracted with Et_2O (3 × 10 mL) and the combined organics washed with water (15 mL), brine (15 mL), dried ($MgSO_4$), filtered and concentrated *in vacuo*. The residue was further dried under high vacuum for 30 min before dissolving in anhydrous THF (3.0 mL) and cooling to –78 ºC. Aryl Grignard **25** (0.4 M in THF, 1.50 mL, 0.600 mmol, 2.0 equiv) was added dropwise over 90 s and the reaction stirred at –78 ºC for 2 h before removing from the cold bath and stirring at room temperature for 16 h. The reaction was quenched by the addition of saturated aqueous NH_4Cl (15 mL) and the phases separated. The aqueous phase was extracted with Et_2O (3 × 10 mL) and the combined organics washed with water (15 mL), brine (15 mL), dried ($MgSO_4$), filtered and concentrated *in vacuo*. The residue was purified by FCC (2 × SiO_2, 30% EtOAc/pet. ether followed by 1% acetone/CH_2Cl_2) to give the title compound (105 mg, 0.193 mmol, 65%) as a colourless oil that crystallized upon standing. The diastereomeric ratio was determined by ^{1}H NMR to be 95:5. This ratio could be improved to >95:5 by recrystallization from Et_2O/pet. ether.

Boronic ester **15a** could also be prepared without aqueous work-up of intermediate **24**. However, this results in diminished yield and diastereoselectivity (54%, 80:20 dr, see Table S5).

Mpt: 153–155 ºC (Et_2O/pet. ether). **Mpt** (racemate): 132–134 ºC (Et_2O/pet. ether).

R_f: 0.35 (2% acetone/DCM).

^{1}H NMR (500 MHz, $CDCl_3$) δ_H 6.95 (s, 1H), 6.59 (s, 1H), 6.50 (s, 1H), 6.48 (s, 1H), 3.87 (s, 3H), 3.87 (s, 3H), 3.83 (s, 3H), 3.80 (s, 3H), 3.78 (s, 3H), 3.70 (s, 3H), 3.05 (br s, 1H), 3.02 (d, *J* = 6.0 Hz, 1H), 2.13–2.06 (m, 1H), 1.96 (dqd, *J* = 14.5, 7.2, 4.2 Hz, 1H), 1.63–1.53 (m, 1H), 1.26 (s, 6H), 1.24 (s, 6H), 0.76 (d, *J* = 6.9 Hz, 3H), 0.71 (t, *J* = 7.3 Hz, 3H) ppm.

^{13}C NMR (126 MHz, $CDCl_3$) δ_C 152.6, 151.7, 147.1, 147.0, 143.0, 142.5, 125.2, 123.1, 114.9, 112.7 (br), 98.1, 97.8, 82.8, 56.8, 56.7, 56.6, 56.4, 56.1, 56.0, 43.2 (br), 39.5, 27.7 (br), 25.8, 24.9, 24.8, 16.4, 12.1 ppm.

^{11}B NMR (96 MHz, $CDCl_3$) δ_B 32.1 (br s) ppm.

IR (ν_{max}/cm^{-1}, neat): 2975–2832, 1509, 1464, 1371, 1313, 1204, 1144, 1038.

HRMS (ESI$^+$) calcd. for $C_{30}H_{45}O_8BNa$ $[M+Na]^+$ 567.3105, found 567.3081.

$[\alpha]_D^{24}$ +26.2 (*c* 0.89, $CHCl_3$).

5,5'-((3*S*,4*S*,5*R*)-4-Methylhept-1-yne-3,5-diyl)bis(1,2,4-trimethoxybenzene) (5)

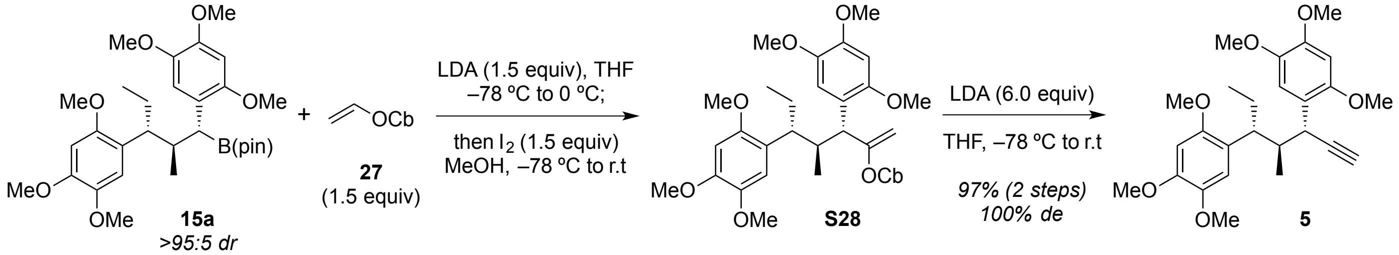

Following a modified literature procedure.[20] To a solution of boronic ester **15a** (132 mg, 0.242 mol, 1.00 equiv) and vinyl carbamate **27** (62 mg, 0.36 mmol, 1.5 equiv) in anhydrous THF (2.40 mL) at –78 ºC was added LDA (0.86 M in THF, 0.42 mL, 0.36 mmol, 1.5 equiv) dropwise over 3 min. The reaction was stirred at –78 ºC for 60 min before the reaction vessel was transferred to a 0 ºC bath and stirred for 30 min. The reaction was cooled to –78 ºC and a solution of I_2 (92 mg, 0.36 mmol, 1.5 equiv) in MeOH (2.40 mL) was added dropwise over 5 min. The mixture was stirred at –78 ºC for 10 min before removing from the cold bath and stirring at room temperature for 60 min. Saturated aqueous $Na_2S_2O_3$ (10 mL) was added and the product extracted into Et_2O (3 × 10 mL). The combined organic extracts were washed with water (10 mL), brine (10 mL), dried ($MgSO_4$), filtered and concentrated *in vacuo*. The residue containing **S28** was further dried under high vacuum for 30 min before placing under an atmosphere of N_2, dissolving in anhydrous THF (2.40 mL) and cooling to –78 ºC. LDA (0.86 M in THF, 2.11 mL, 1.82 mmol, 6.00 equiv) was added dropwise over 1 min and the resulting solution was removed from the cold bath and stirred at room temperature for 15 min. The reaction was quenched by the addition of saturated aqueous NH_4Cl (10 mL) and the product extracted into Et_2O (3 × 10 mL). The combined organic extracts were washed with brine (10 mL), dried ($MgSO_4$), filtered and concentrated *in vacuo*. The residue was purified by FCC (SiO_2, 25% EtOAc/pet. ether) to give the title compound (104 mg, 0.235 mmol, 97%) as a colourless oil. The reaction was determined by ^{1}H NMR analysis to proceed with 100% diastereomeric excess (de)

R_f: 0.29 (25% EtOAc/pet. ether).

^{1}H NMR (500 MHz, $CDCl_3$) δ_H 7.17 (s, 1H), 6.63 (s, 1H), 6.52 (s, 1H), 6.51 (s, 1H), 4.55 (t, *J* = 2.9 Hz, 1H), 3.89 (s, 3H), 3.88 (s, 3H), 3.86 (s, 3H), 3.84 (s, 3H), 3.82 (s, 3H), 3.79 (s, 3H), 3.20 (br s, 1H), 2.28 (d, *J* = 2.6 Hz, 1H), 2.07 (dqd, *J* = 13.4, 7.4, 3.9 Hz, 1H), 2.01 (br s, 1H), 1.68 (br s, 1H), 0.76 (t, *J* = 7.3 Hz, 3H), 0.58 (d, *J* = 6.7 Hz, 3H) ppm.

^{13}C NMR (126 MHz, $CDCl_3$) δ_C 152.6 (br), 150.2, 148.2, 147.4, 143.1 (br), 142.5, 124.8 (br), 120.8, 114.3, 111.9 (br), 98.2, 97.4, 83.6, 72.2, 57.0 (br), 56.8, 56.7, 56.23, 56.22, 56.0, 41.4 (br), 40.6 (br), 33.9, 24.9 (br), 13.4, 11.8 (br) ppm.

IR (ν_{max}/cm^{-1}, neat): 3287, 2993–2835, 1509, 1464, 1458, 1439, 1398, 1316, 1238, 1204, 1183, 1036.

HRMS (ESI$^+$) calcd. for $C_{26}H_{34}O_6Na$ [M+Na]$^+$ 465.2248, found 465.2252.

$[\alpha]_D^{22}$ +67.6 (*c* 0.88, $CHCl_3$).

5,5'-((3*R*,4*S*,5*R*,*E*)-1-Iodo-2,4-dimethylhept-1-ene-3,5-diyl)bis(1,2,4-trimethoxybenzene) (28)

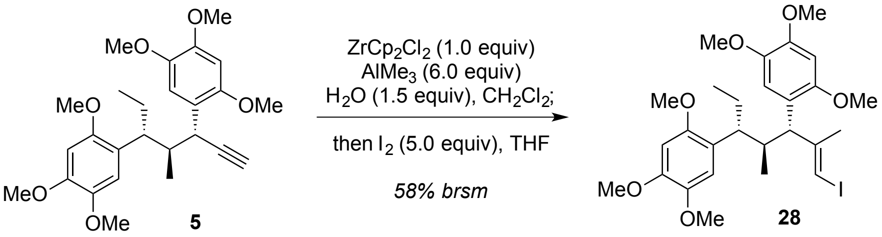

Following a modified literature procedure.[21] A solution of zirconocene dichloride (23 mg, 0.079 mmol, 1.0 equiv) in anhydrous CH_2Cl_2 (0.80 mL) was degassed by three freeze/pump/thaw cycles before cooling to –78 ºC. Trimethylaluminium (2 M in toluene, 0.24 mL, 0.47 mmol, 6.0 equiv) was added followed by H_2O (2.1 µL, 0.12 mmol, 1.5 equiv) and the reaction was transferred to a 0 ºC bath and stirred for 30 min, giving a bright yellow solution, before cooling to –78 ºC. In a separate flask, a solution of alkyne **5** (35 mg, 0.079 mmol, 1.0 equiv) in anhydrous CH_2Cl_2 (0.80 mL) was degassed by three freeze/pump/thaw cycles. This solution was added dropwise over 1 min to the $ZrCp_2Cl_2/AlMe_3$ mixture. The cold bath was removed and the reaction was stirred at room temperature for 18 h before cooling back to –78 ºC. A solution of I_2 (100 mg, 0.395 mmol, 5.00 equiv) in degassed anhydrous THF (0.40 mL) was added dropwsie over 2 min and the resulting mixture stirred at –78 ºC for 30 min, 0 ºC for 30 min, then at room temperature for 30 min. The reaction was cooled to 0 ºC and quenched by the slow [Gas evolution!] addition of saturated aqueous K_2CO_3 (2 mL). The phases were separated, the aqueous phase was extracted with CH_2Cl_2 (3 × 5 mL) and the combined organic extracts were dried ($MgSO_4$), filtered, and concentrated *in vacuo*. The residue was purified by preparative TLC (2 × SiO_2, 1st = 15% EtOAc/toluene, 2nd = 0.5% EtOAc/CH_2Cl_2) to give the title compound (16 mg, 0.027 mmol, 35%, 58% brsm) as a colourless oil, as well as recovered alkyne **5** (14 mg, 0.031 mmol, 40%).

R_f: 0.51 (1% EtOAc/CH_2Cl_2), 0.40 (15% EtOAc/toluene).

^{1}H NMR (500 MHz, $CDCl_3$) δ_H 6.81 (s, 1H), 6.51 (s, 2H), 6.39 (s, 1H), 5.95 (d, *J* = 1.2 Hz, 1H), 3.90 (s, 3H), 3.87 (s, 3H), 3.86 (s, 3H), 3.77 (s, 3H), 3.71 (s, 3H), 3.57 (d, *J* = 11.4 Hz, 1H), 3.44 (s, 3H), 3.04 (br s, 1H), 2.42 (dqd, *J* = 13.7, 6.7, 3.1 Hz, 1H), 1.79–1.62 (m, 2H), 1.62 (d, *J* = 1.0 Hz, 3H), 0.81 (d, *J* = 6.8 Hz, 3H), 0.77 (t, *J* = 7.3 Hz, 3H) ppm.

^{13}C NMR (126 MHz, $CDCl_3$) δ_C 152.8, 152.3, 148.1, 147.8, 147.5, 142.5, 142.0, 121.8, 121.4, 114.7, 112.2, 97.9, 96.8, 77.2, 56.9, 56.7, 56.2, 56.1, 56.0, 55.5, 50.8, 36.6, 26.8, 20.3, 14.6, 12.9 ppm. One signal (corresponding to Ar**C**HCH_2) is not observed.

IR (ν_{max}/cm^{-1}, neat): 2961–2832, 1508, 1463, 1455, 1316, 1203, 1179, 1037.

HRMS (ESI$^+$) calcd. for $C_{27}H_{37}O_6INa$ $[M+Na]^+$ 607.1527, found 607.1513.

$[\alpha]_D^{23}$ +91.1 (*c* 0.90, $CHCl_3$).

Tatanan A (1)

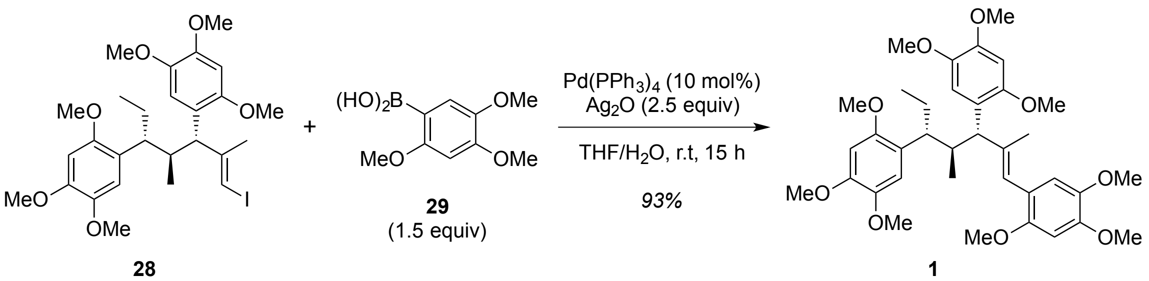

A vial was charged with vinyl iodide **28** (25 mg, 0.043 mmol, 1.0 equiv), 2,4,5-trimethoxyphenylboronic acid (**29**) (14 mg, 0.064 mmol, 1.5 equiv), $Pd(PPh_3)_4$ (4.9 mg, 4.3 μmol, 10 mol%), and Ag_2O (25 mg, 0.11 mmol, 2.5 equiv). The vial was sealed with a septum and triple evacuated/N_2 filled before the addition of degassed (sparged with N_2 for 10 min) THF/H_2O (5:1, 0.86 mL). The mixture was stirred at room temperature for 16 h before filtering through a plug of silica, eluting with EtOAc, and concentrating *in vacuo*. The residue was purified by preparative TLC (SiO_2, 25% EtOAc/toluene) to give the title compound (25 mg, 0.040 mmol, 93%) as a white solid. Recrystallization from hot MeOH gave colourless crystals.

Mpt: 136–138 ºC (MeOH) [Lit. 125–127 ºC (MeOH), 123–125 ºC (CD_3OD)].[26,27]

Mpt (racemate): 122–124 ºC (MeOH).

$\mathbf{R_f}$**:** 0.33 (40% EtOAc/pet. ether).

^{1}H NMR (500 MHz, CD_3OD) δ_H 6.86 (s, 1H), 6.65 (s, 1H), 6.63 (s, 1H), 6.59 (s, 1H), 6.53 (s, 1H), 6.52 (br s, 1H), 6.26 (s, 1H), 3.86 (s, 3H), 3.84 (s, 3H), 3.81 (s, 3H), 3.77 (s, 3H), 3.76 (s, 3H), 3.71 (s, 3H), 3.70 (s, 6H), 3.52 (s, 3H), 3.51 (d, *J* = 10.5 Hz, 1H), 3.10 (br s, 1H), 2.55 (dqd, *J* = 10.4, 6.8, 3.1 Hz, 1H), 1.84–1.68 (m, 2H), 1.55 (s, 3H), 0.95 (d, *J* = 6.8 Hz, 3H), 0.75 (t, *J* = 7.3 Hz, 3H) ppm.

^{1}H NMR (500 MHz, $CDCl_3$) δ_H 6.89 (s, 1H), 6.64 (s, 1H), 6.54 (s, 2H), 6.48 (s, 1H), 6.43 (s, 1H), 6.34 (s, 1H), 3.91 (s, 3H), 3.89 (s, 3H), 3.87 (s, 3H), 3.86 (s, 3H), 3.80 (s, 3H), 3.78 (s, 3H), 3.75 (s, 3H), 3.72 (s, 3H), 3.53 (d, *J* = 11.3 Hz, 1H), 3.50 (s, 3H), 3.08 (br s, 1H), 2.51 (dqd, *J* = 11.2, 6.9, 3.4 Hz, 1H), 1.81–1.62 (m, 2H), 1.60 (d, *J* = 1.3 Hz, 3H), 0.93 (d, *J* = 6.8 Hz, 3H), 0.79 (t, *J* = 7.3 Hz, 3H) ppm.

^{13}C NMR (126 MHz, CD_3OD) δ_C 153.2, 152.8, 152.1, 148.4, 147.79, 147.75, 142.4, 142.3, 142.0, 138.2, 123.6, 122.3, 121.6, 119.9, 115.3, 115.1, 113.8, 98.53, 98.46, 97.2, 56.4, 56.20, 56.16, 55.9, 55.5, 55.41, 55.35, 55.3, 54.7, 50.6, 40.5 (br), 36.9, 25.8, 14.4, 13.5, 11.9 ppm.

^{13}C NMR (126 MHz, $CDCl_3$) δ_C 152.8, 152.5, 151.7, 148.0, 147.3, 147.3, 142.5, 142.4, 142.0, 138.5, 123.8, 122.3, 121.8, 120.1, 114.7 (br), 114.5, 112.7, 98.5, 98.2, 96.9, 57.0, 56.7, 56.7, 56.6, 56.4, 56.2, 56.1, 56.0, 55.6, 51.0, 37.0, 26.8, 14.8, 14.5, 12.9 ppm. One signal (corresponding to Ar**C**HCH$_2$) is not observed.

IR (ν_{max}/cm^{-1}, neat): 2958–2832, 1508, 1464, 1455, 1439, 1396, 1317, 1204, 1178, 1037.

HRMS (ESI$^+$) calcd. for $C_{36}H_{48}O_9Na$ $[M+Na]^+$ 647.3191, found 647.3188.

$[\alpha]_D^{24}$ +90.0 (*c* 0.10, MeOH) [Lit: $[\alpha]_D^{20}$ +10 (*c* 0.1 MeOH),[26] $[\alpha]_D^{23}$ +55.1 (*c* 0.1 MeOH)].[27]

[26] G. Ni, Z.-F. Shen, Y. Lu, Y.-H. Wang, Y.-B. Tang, R.-Y. Chen, Z.-Y. Hao, D.-Q. Yu, *J. Org. Chem.* **2011**, *76*, 2056 – 2061.
[27] Q. Xiao, J. J. Jackson, A. Basak, J. M. Bowler, B. G. Miller, A. Zakarian, *Nat. Chem.* **2013**, *5*, 410 – 416.

12. Comparison of NMR Spectra of Natural and Synthetic Tatanan A

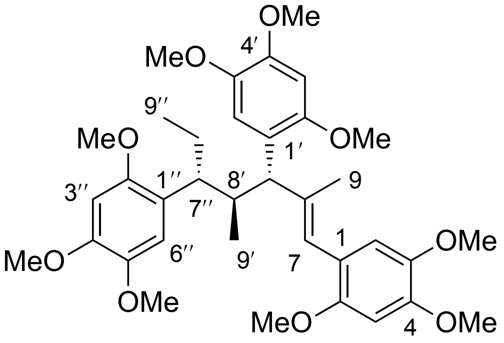

^{1}H NMR (CD$_3$OD):

Natural isolate:§

Synthetic:

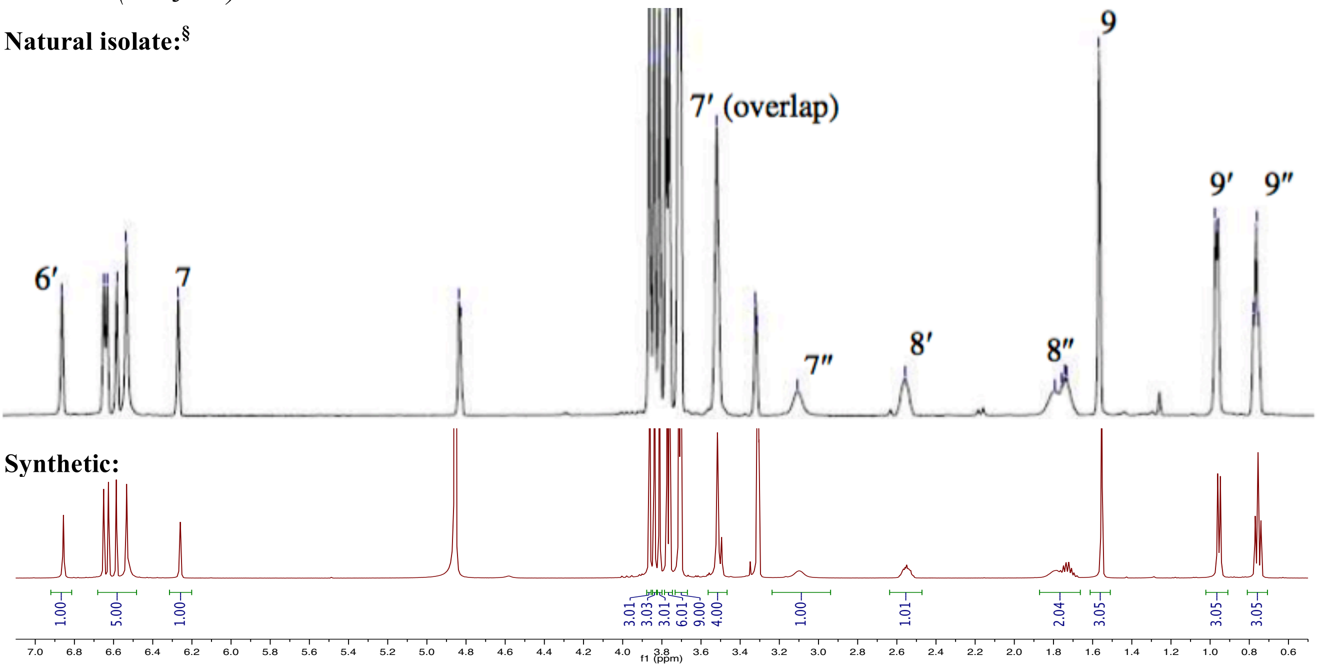

§ NMR spectrum from the Supporting Information of reference 26.

^{13}C NMR (CD$_3$OD):

Natural isolate:§

Synthetic:

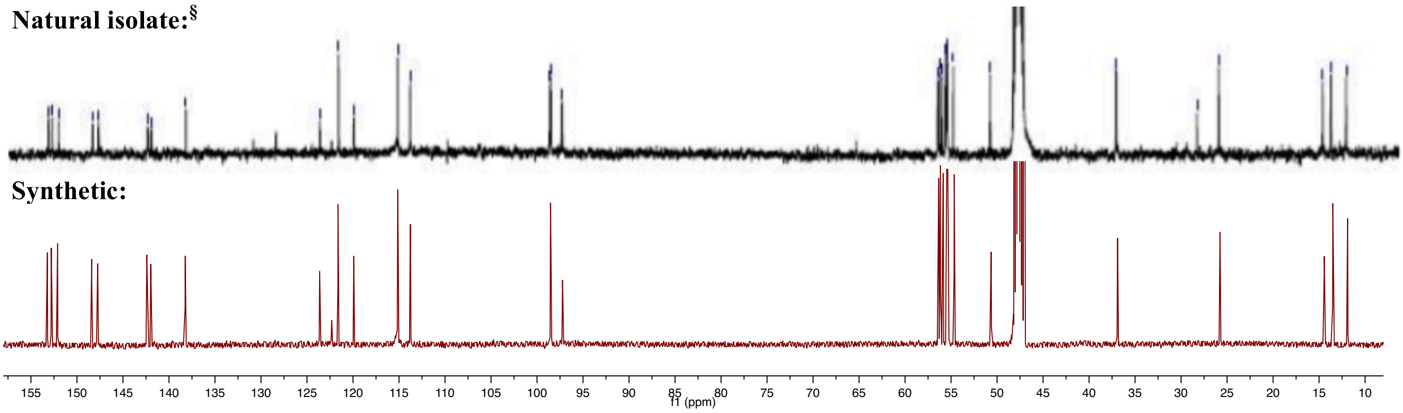

§ NMR spectum from the Supporting Information of reference 26.

^{1}H and ^{13}C NMR Data (CD₃OD):

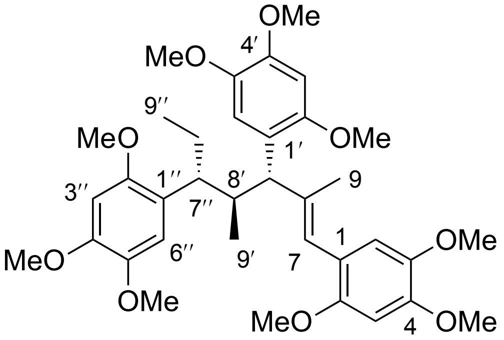

Number	Natural Isolate[26]		Synthetic	
	δ_H (m, *J*)	δ_C	δ_H (m, *J*)[a]	δ_C[b]
1		121.4		121.30
2		153.5		153.49
3	6.59 (s)	98.7	6.59 (s)	98.61
4		149.8		149.78
5		143.8		143.79
6	6.64 (s)	116.6	6.63 (s)	116.71
7	6.28 (s)	123.0	6.26 (s)	123.01
8		139.7		139.62
9	1.57 (s)	14.9	1.55 (s)	14.90
1'		125.1		125.01
2'		154.2		154.18
3'	6.66 (s)	100.0	6.65 (s)	99.91
4'		149.2		149.13
5'		143.7		143.70
6'	6.87 (s)	115.2	6.86 (s)	115.15
7'	3.53 (overlap)	52.0	3.51 (d, 10.5)	52.03
8'	2.56 (1H, m)	38.3	2.55 (dqd, 10.4, 6.8, 3.1)	38.29
9'	0.97 (d, 6.5)	15.8	0.95 (d, 6.8)	15.83
1"		123.8		123.70
2"		154.6		154.60
3"	6.54 (s)	99.9	6.52 (s)	99.85
4"		149.2		149.18
5"		143.4		143.37
6"	6.54 (s)	116.6	6.53 (s)	116.52
7"	3.11 (m)	29.5[c]	3.10 (br s)	41.88 (broad)[c]
8"	a) 1.74 (m); b) 1.80 (m)	27.2	a) 1.73 (m); b) 1.79 (m)	27.15
9"	0.77 (t, 6.5)	13.3	0.75 (t, 7.3)	13.29
OMe	3.53 (s)	56.1	3.52 (s)	56.05
OMe	3.71 (s)	56.7	3.70 (s)	56.66
OMe	3.71 (s)	56.8	3.70 (s)	56.74
OMe	3.71 (s)	56.8	3.71 (s)	56.80
OMe	3.77 (s)	56.9	3.76 (s)	56.86
OMe	3.78 (s)	57.3	3.77 (s)	57.24
OMe	3.82 (s)	57.6	3.81 (s)	57.54
OMe	3.84 (s)	57.6	3.84 (s)	57.58
OMe	3.87 (s)	57.8	3.86 (s)	57.73

[a] Referenced to CD_3OD at 3.31 ppm. [b] Referenced to C-2" at 154.60 ppm. [c] Peak misassigned in the isolation paper due to impurity in the ^{13}C NMR spectrum. The reassigned signal at 41.9 ppm is very broad and easily missed.

13. ^{1}H and ^{13}C NMR spectra

^{1}H NMR (400 MHz, $CDCl_3$):

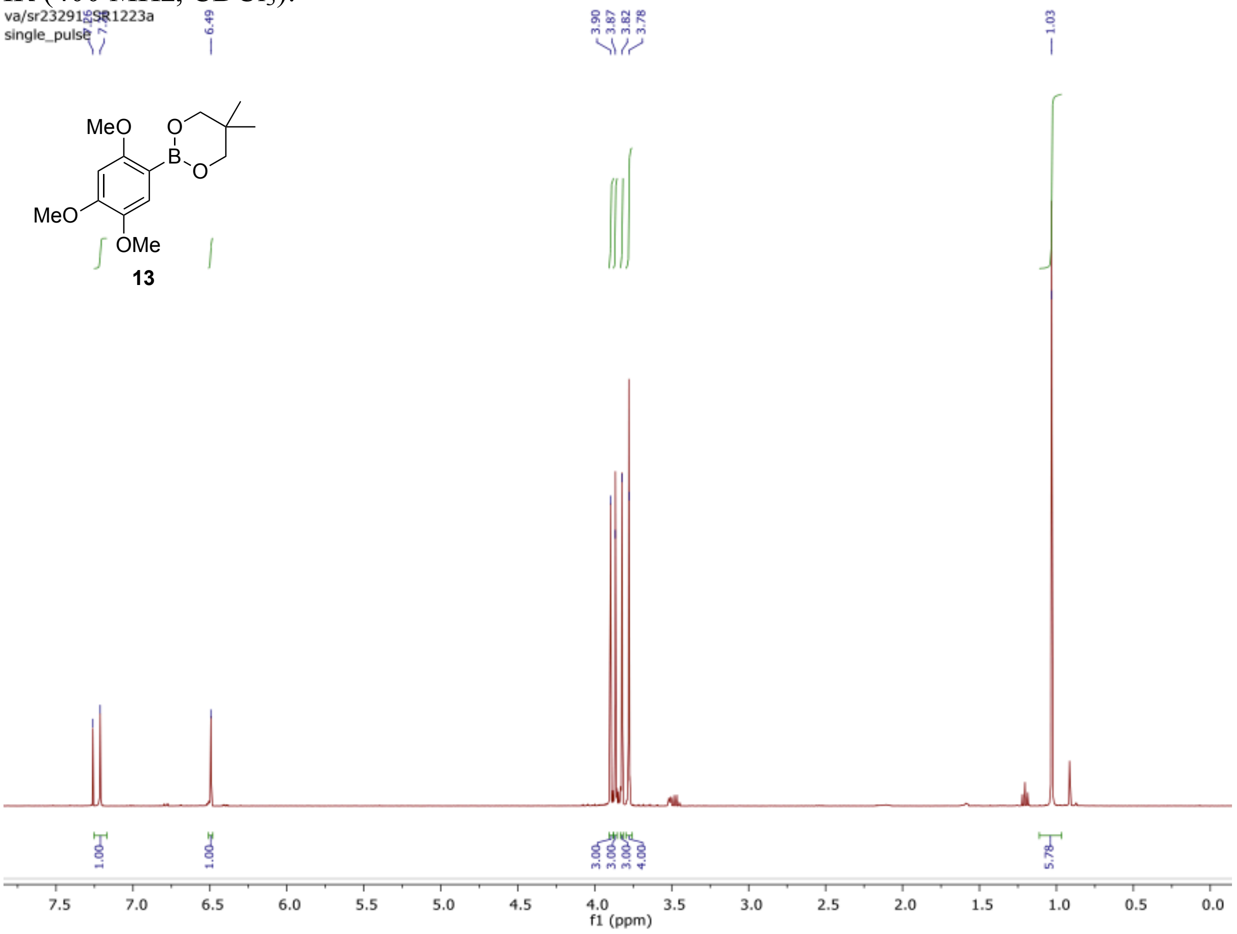

^{13}C NMR (101 MHz, $CDCl_3$):

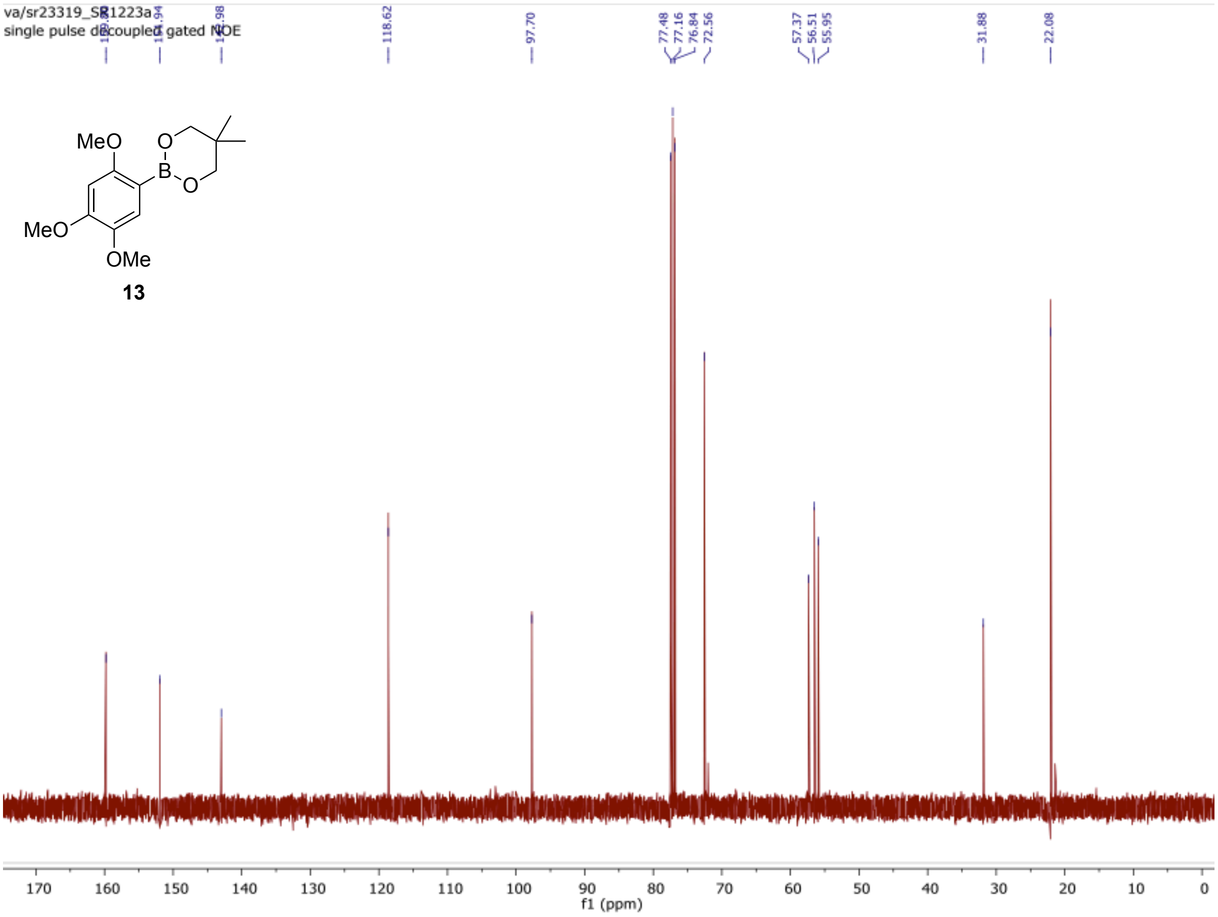

^{1}H NMR (400 MHz, $CDCl_3$):

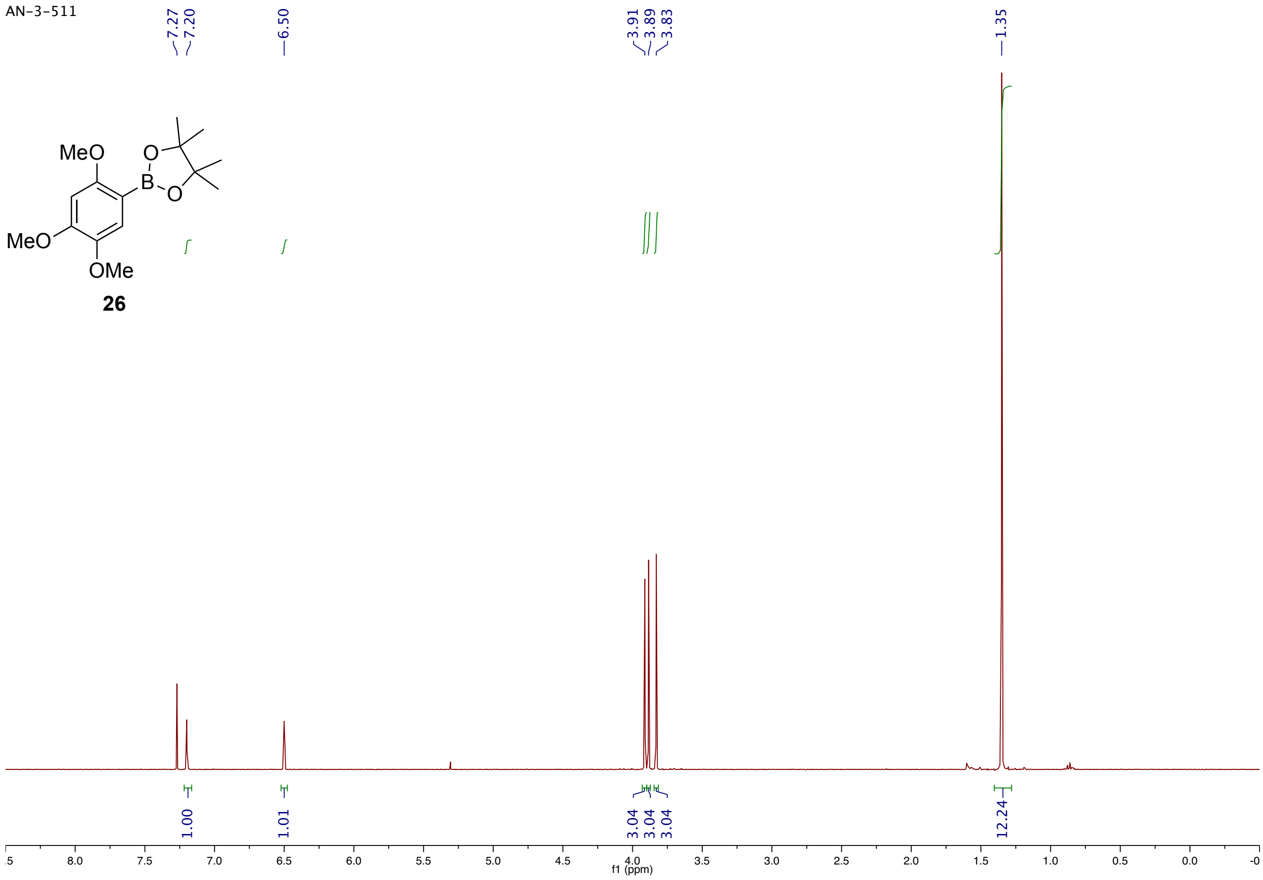

^{13}C NMR (101 MHz, $CDCl_3$):

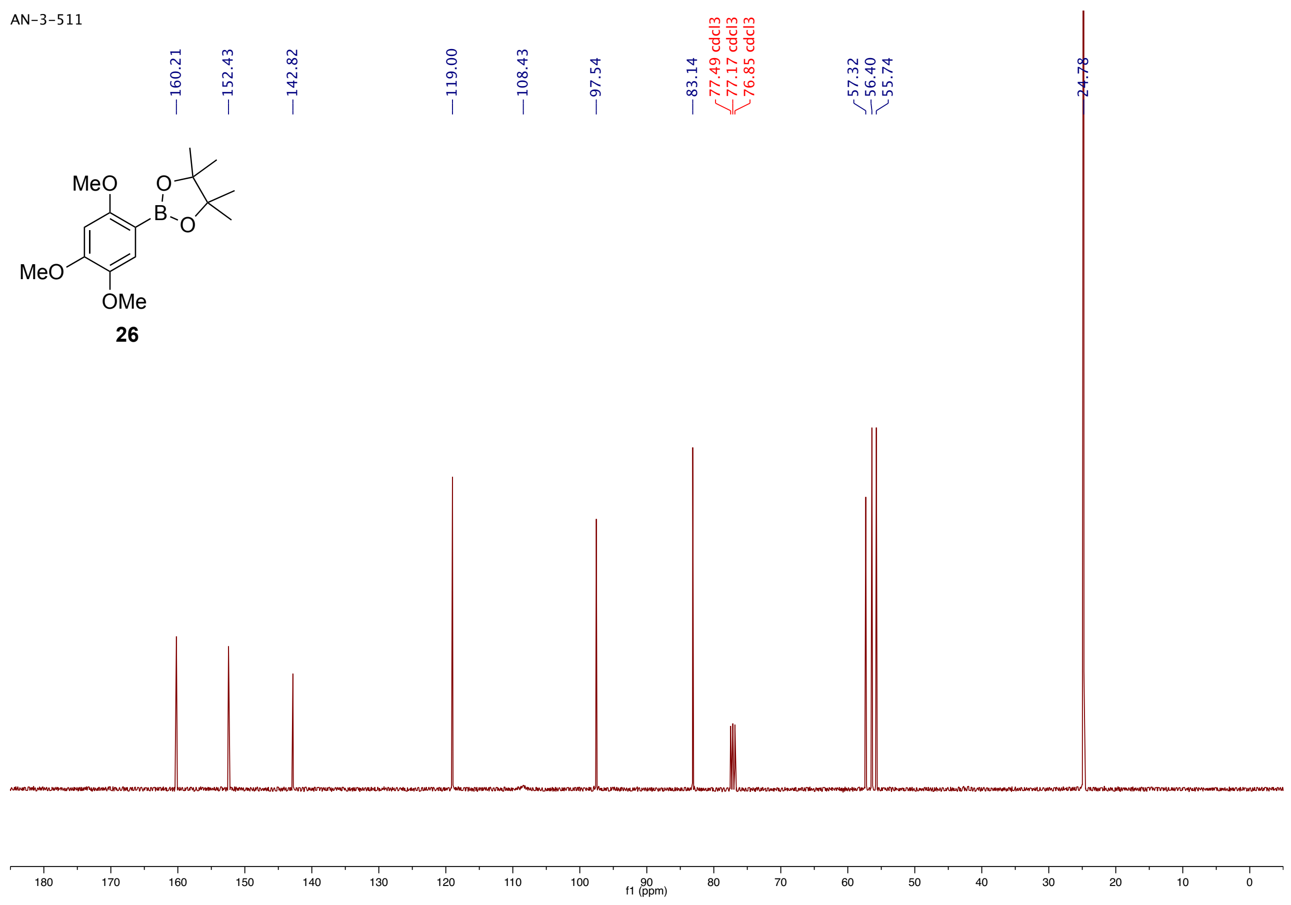

^{1}H NMR (400 MHz, $CDCl_3$):

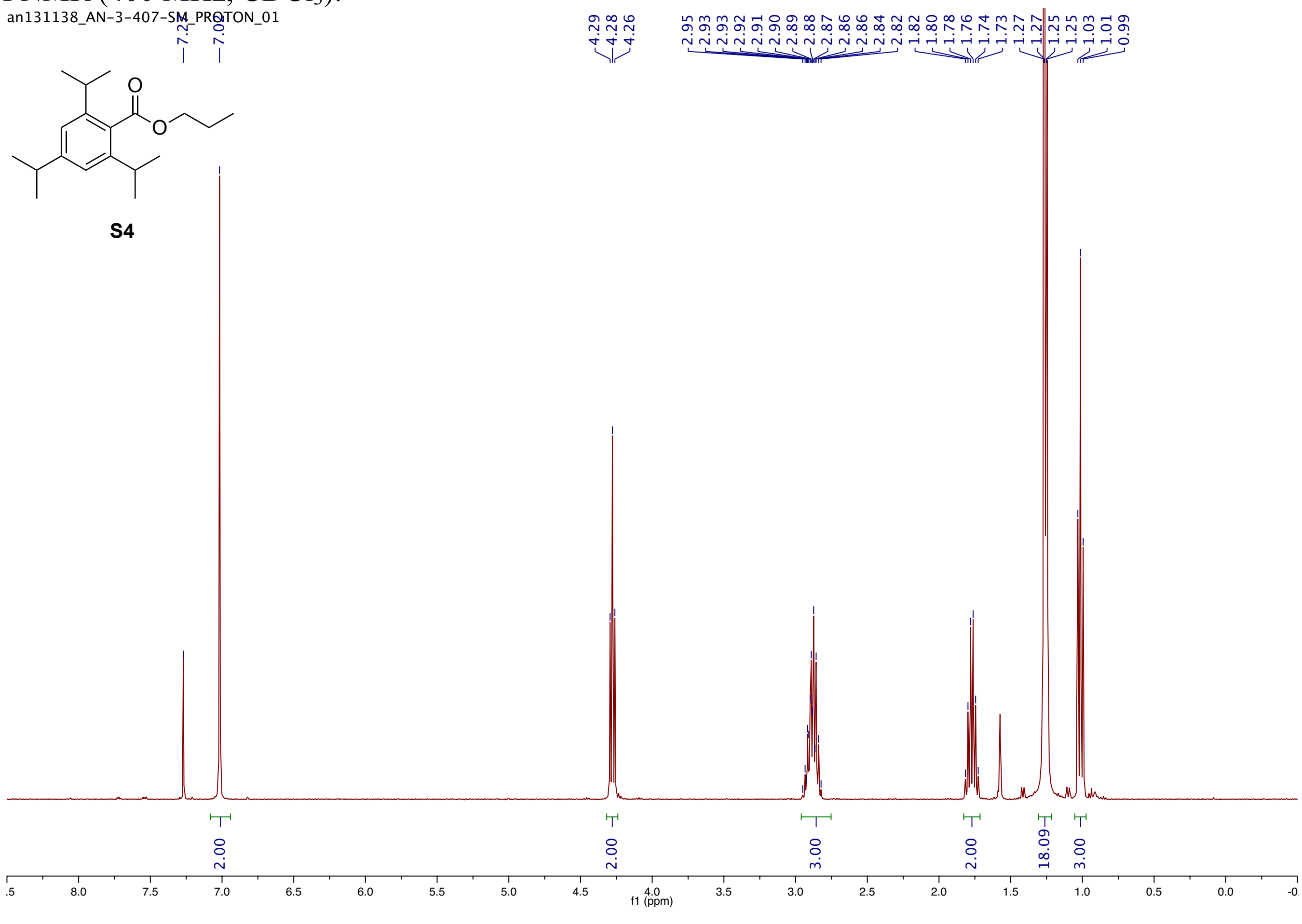

^{13}C NMR (101 MHz, $CDCl_3$):

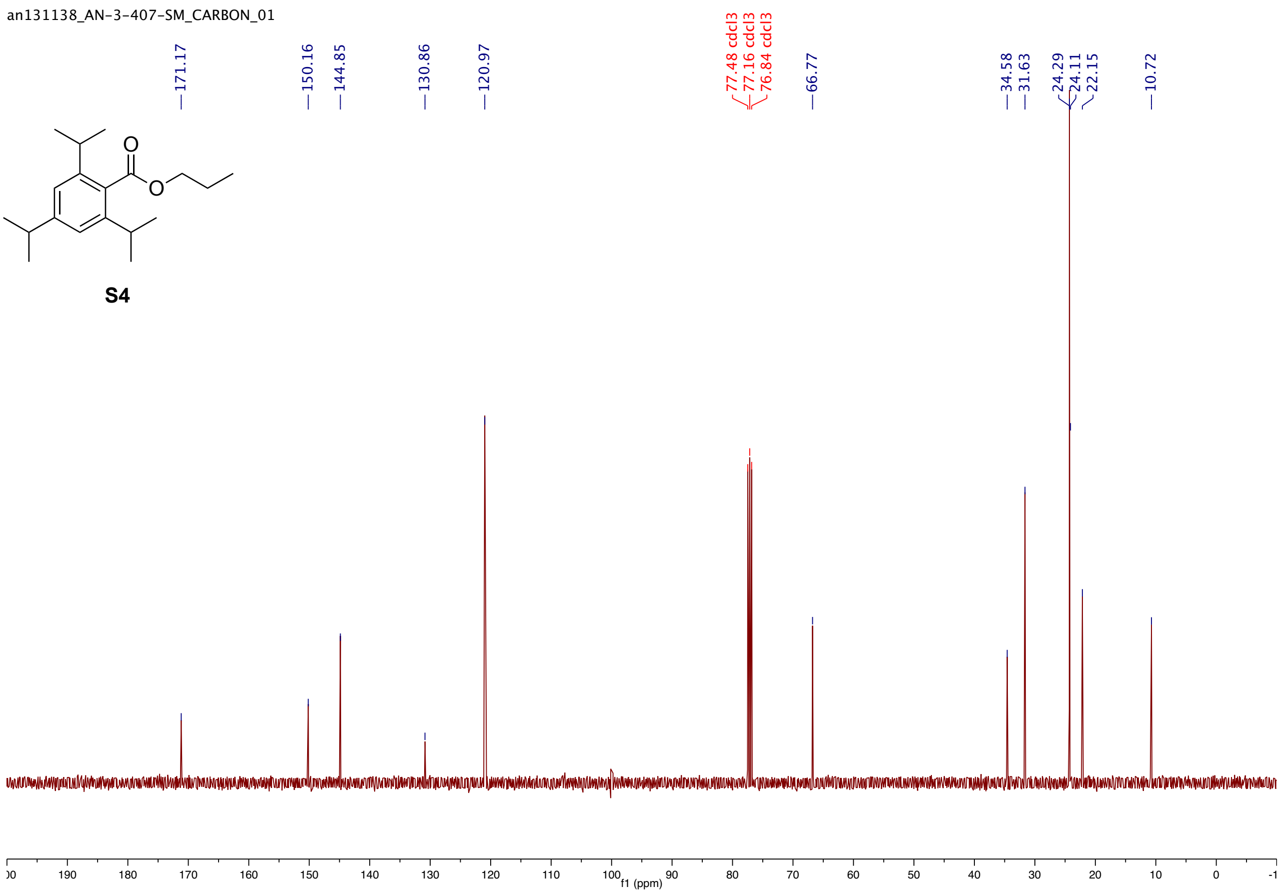

^{1}H NMR (400 MHz, $CDCl_3$):

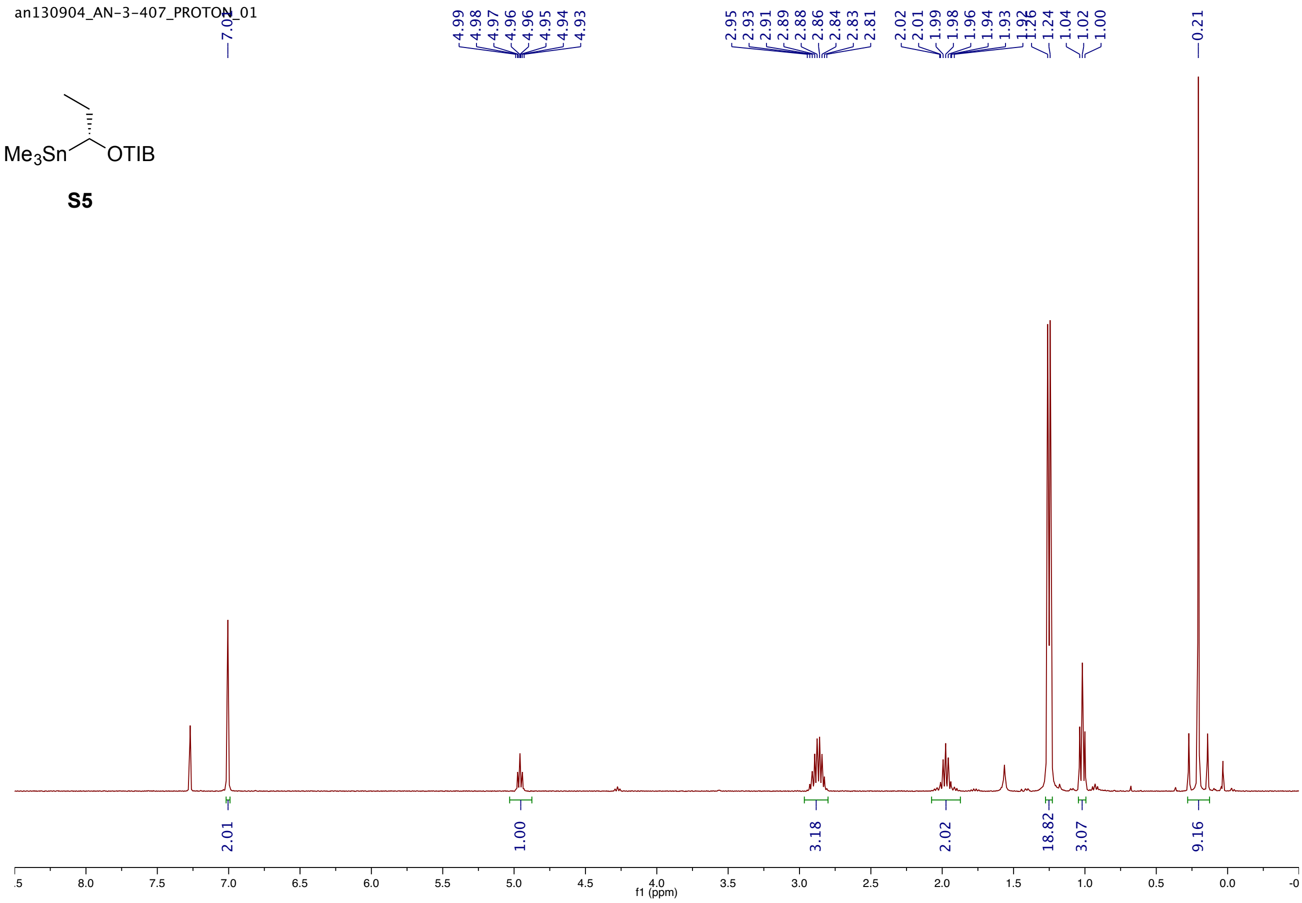

^{13}C NMR (101 MHz, $CDCl_3$):

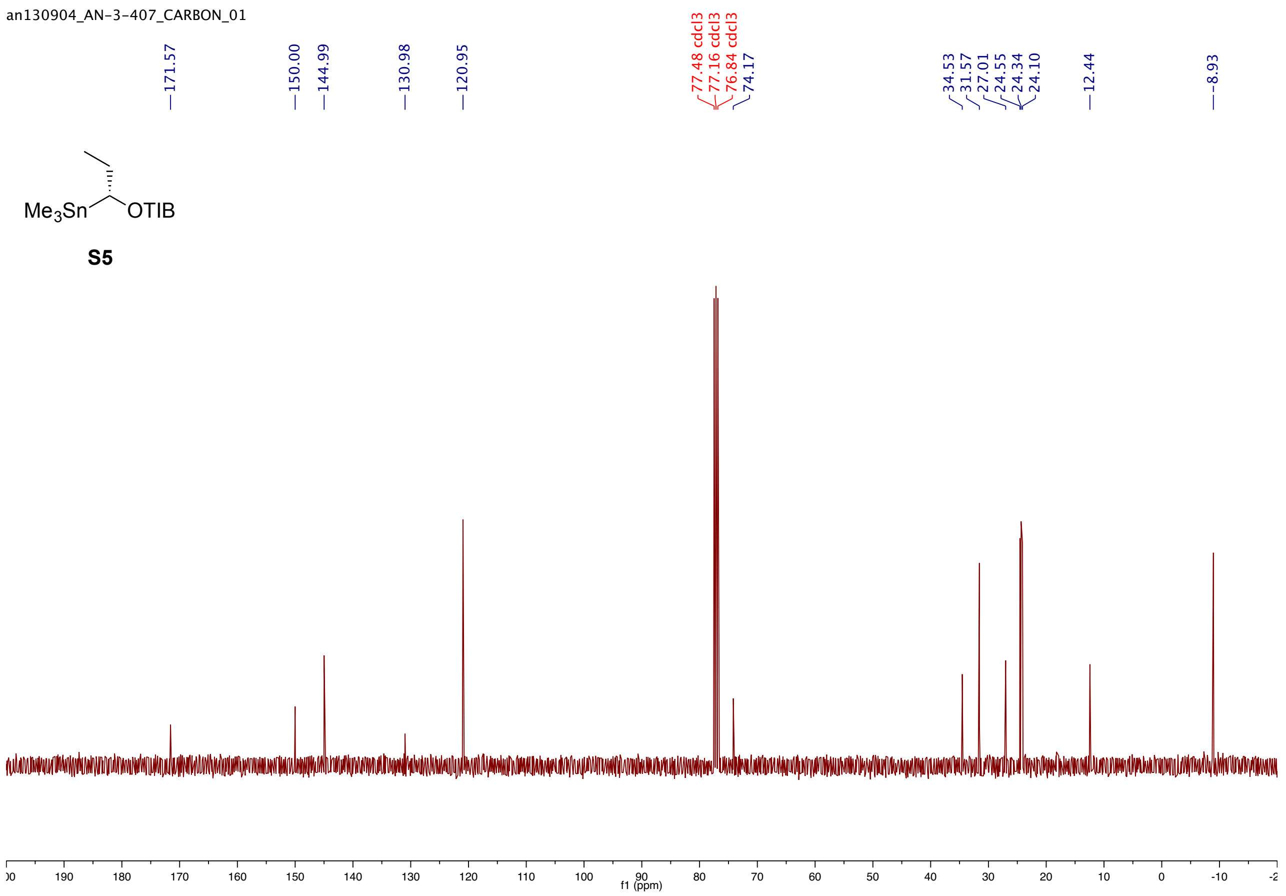

^{1}H NMR (500 MHz, $CDCl_3$):

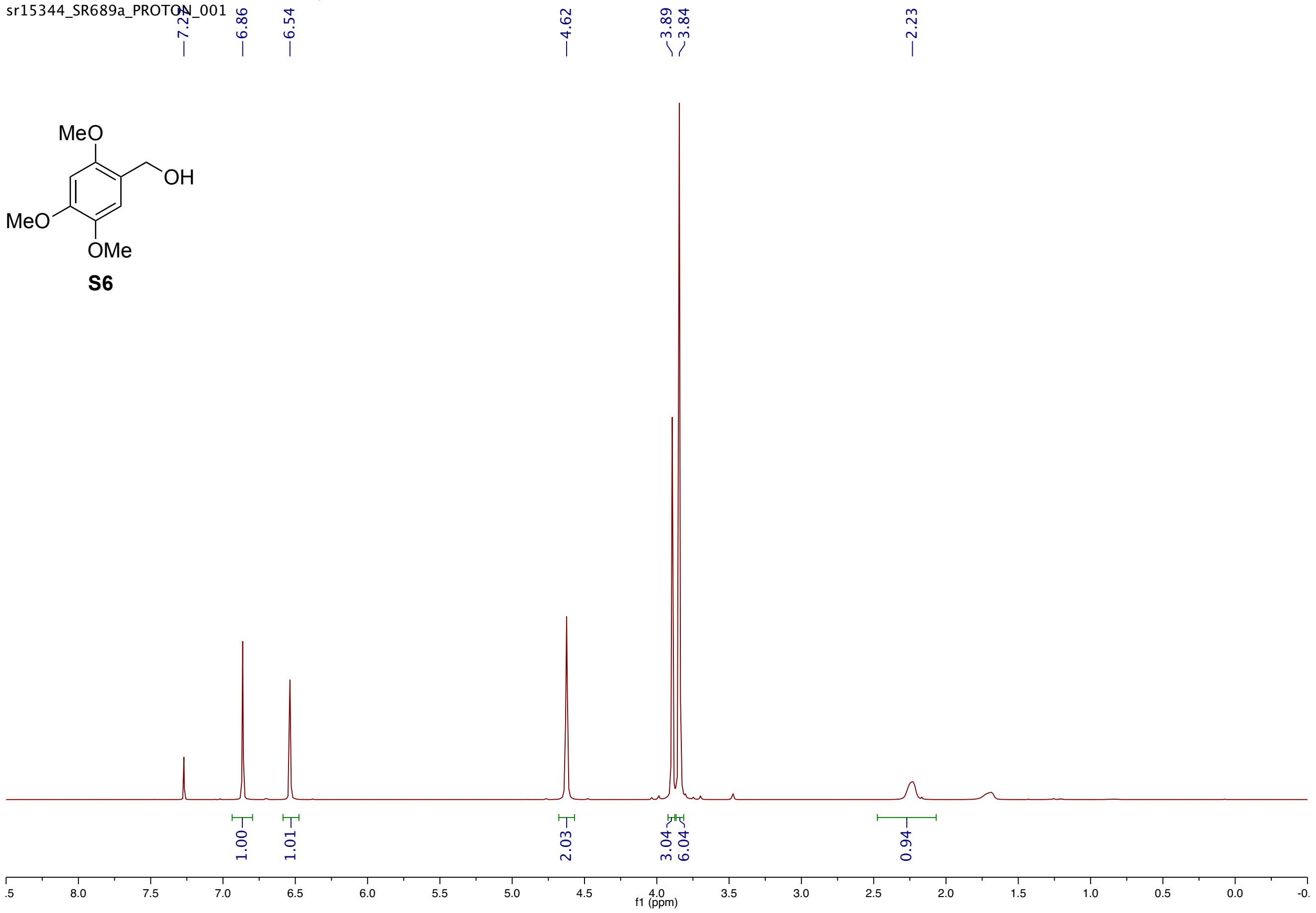

^{13}C NMR (126 MHz, $CDCl_3$):

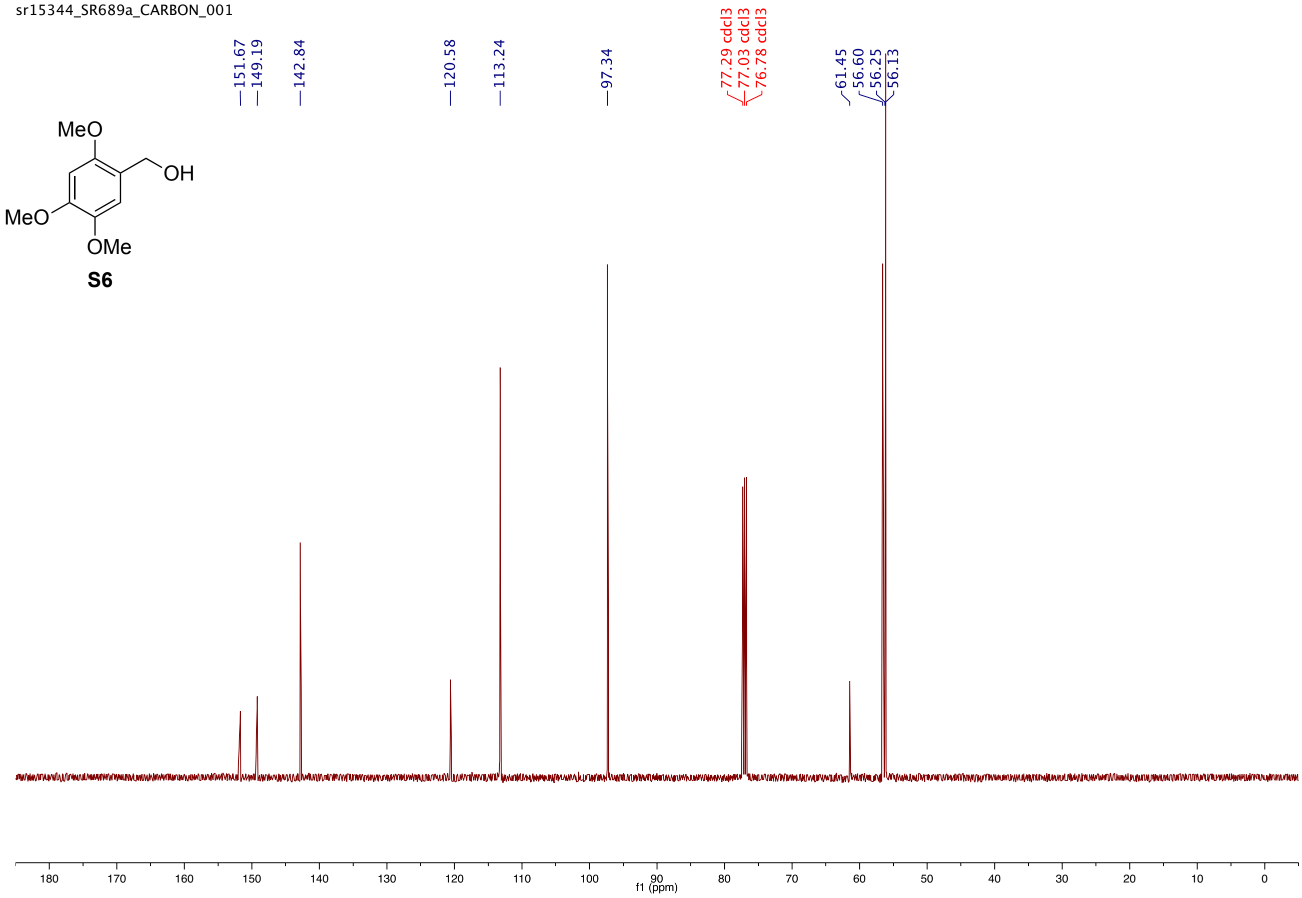

^{1}H NMR (400 MHz, $CDCl_3$):

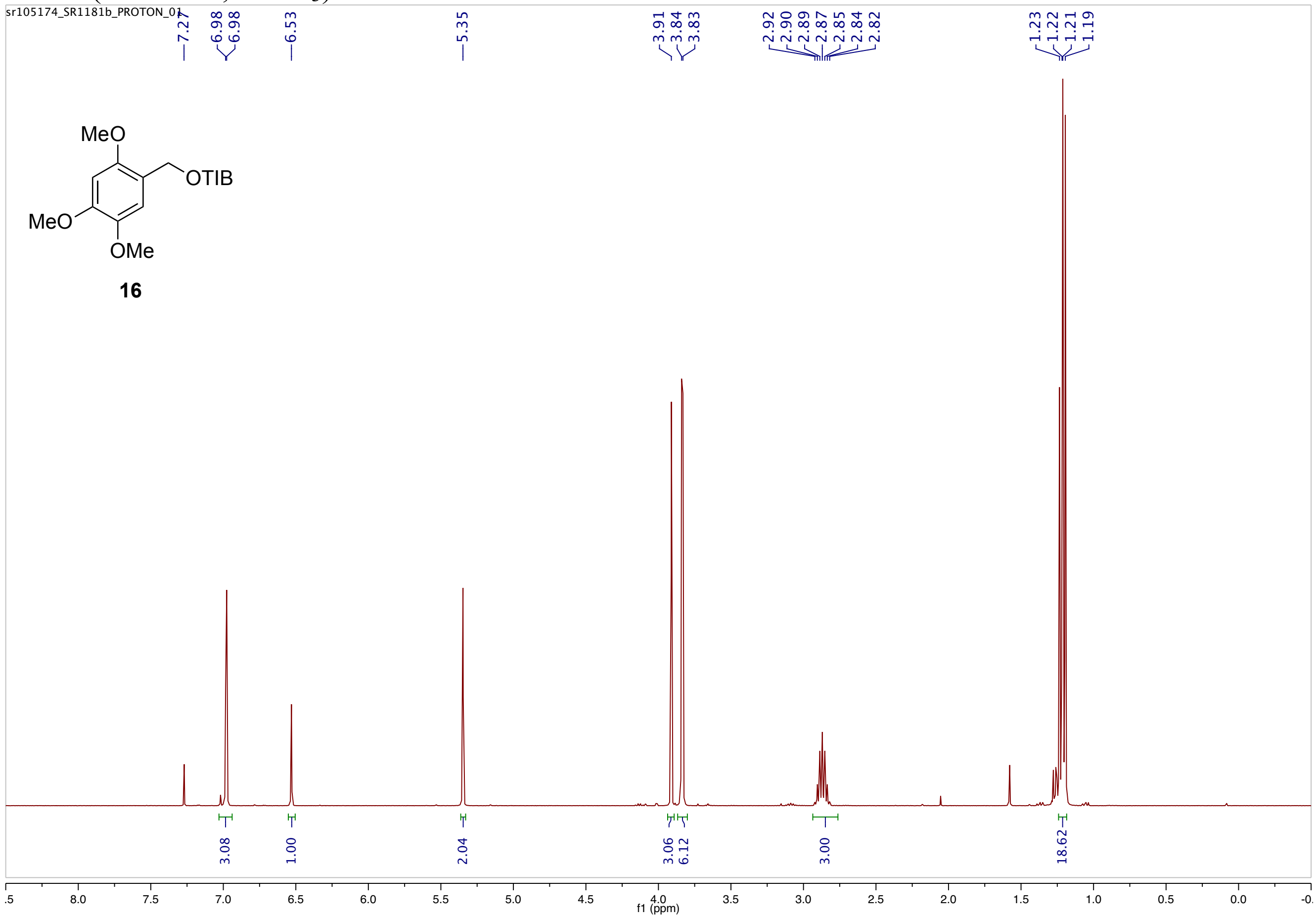

^{13}C NMR (101 MHz, $CDCl_3$):

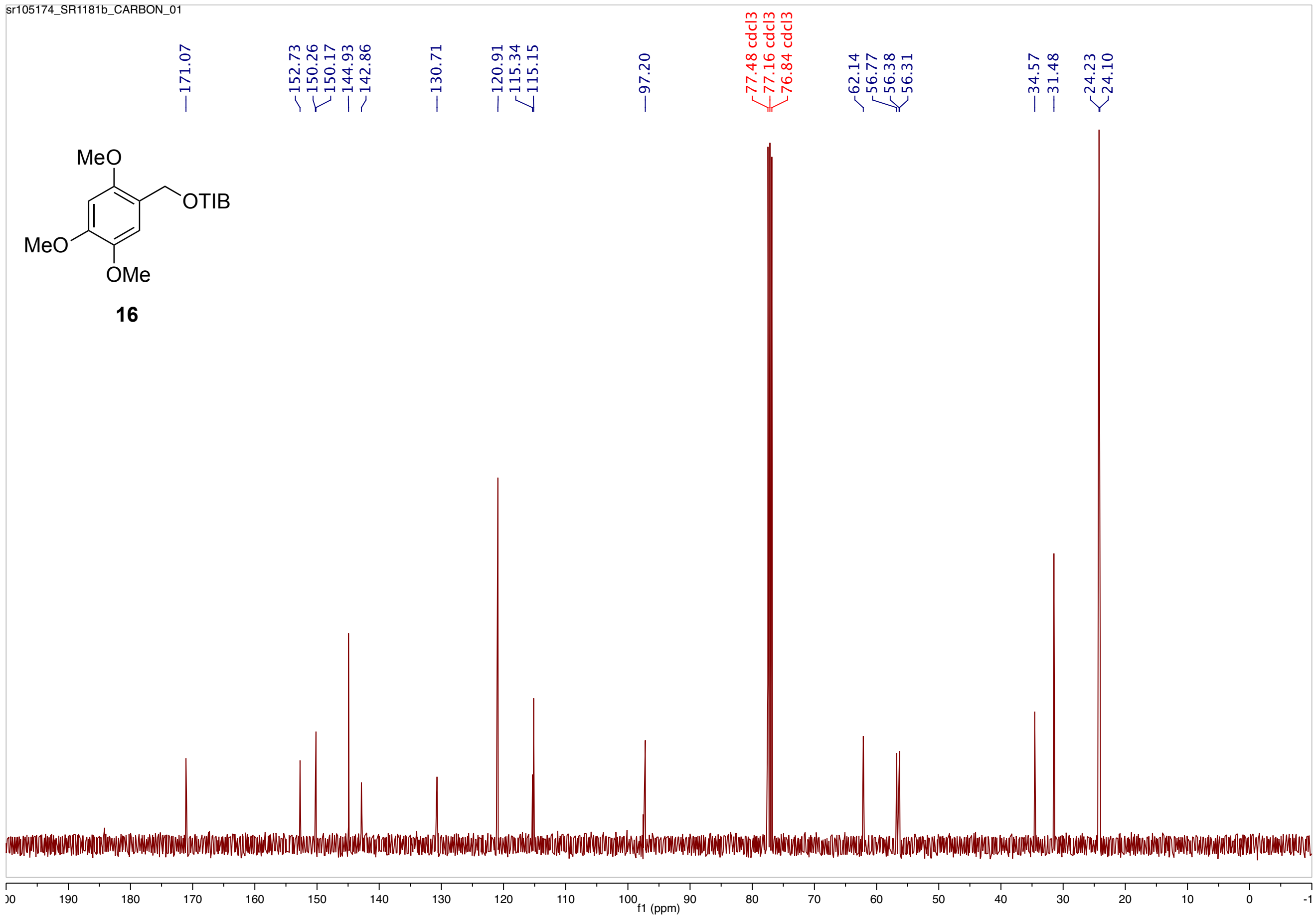

^{1}H NMR (400 MHz, $CDCl_3$):

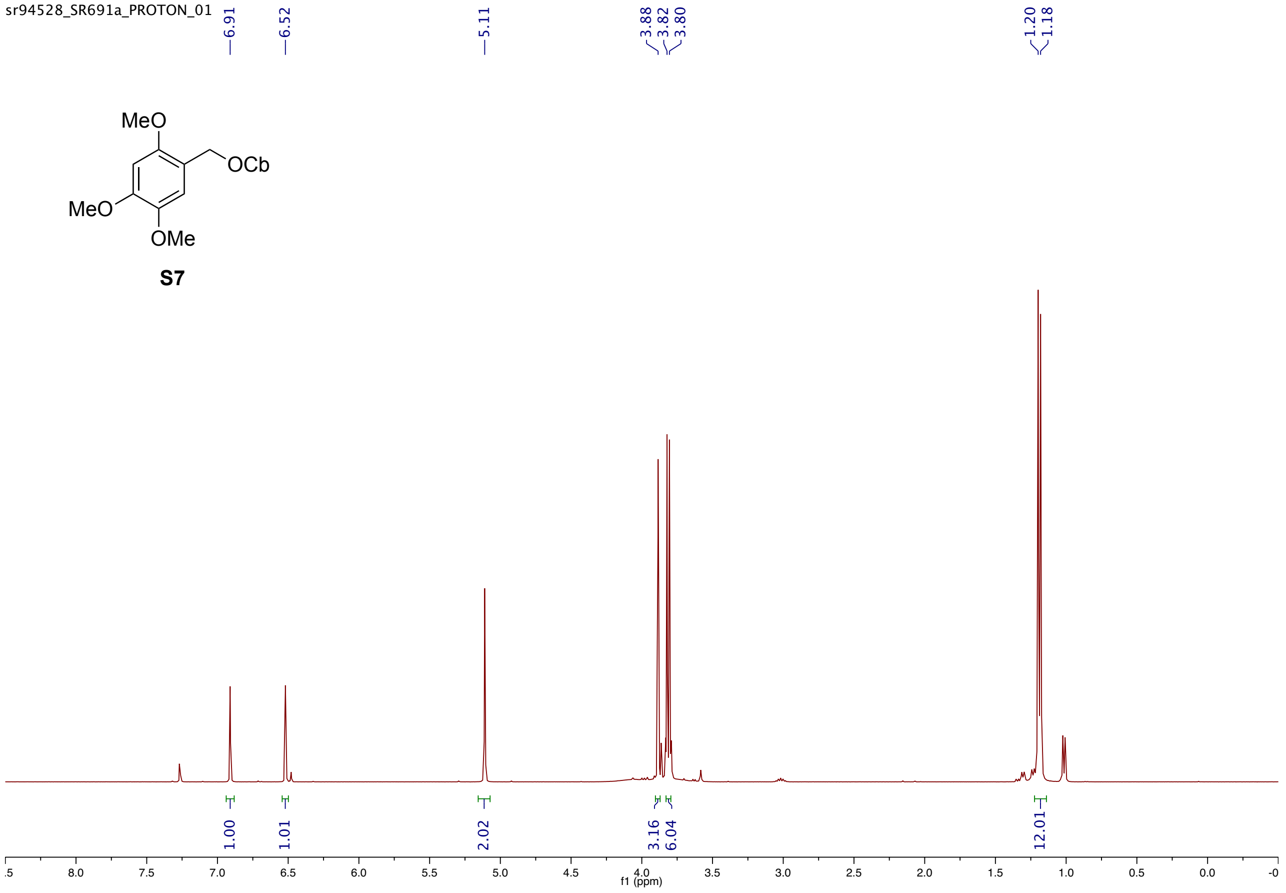

^{13}C NMR (101 MHz, $CDCl_3$):

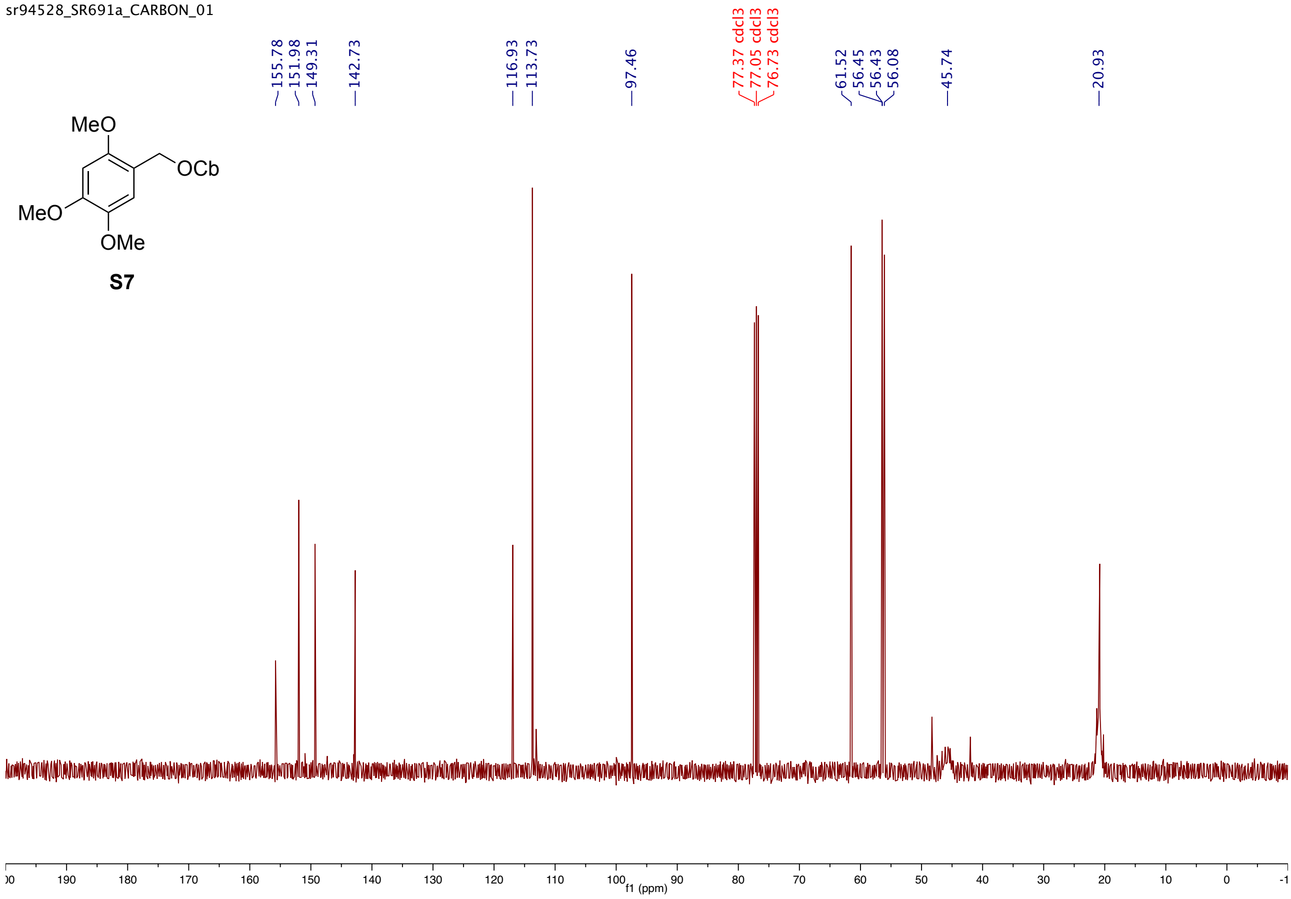

S7

^{1}H NMR (500 MHz, $CDCl_3$):

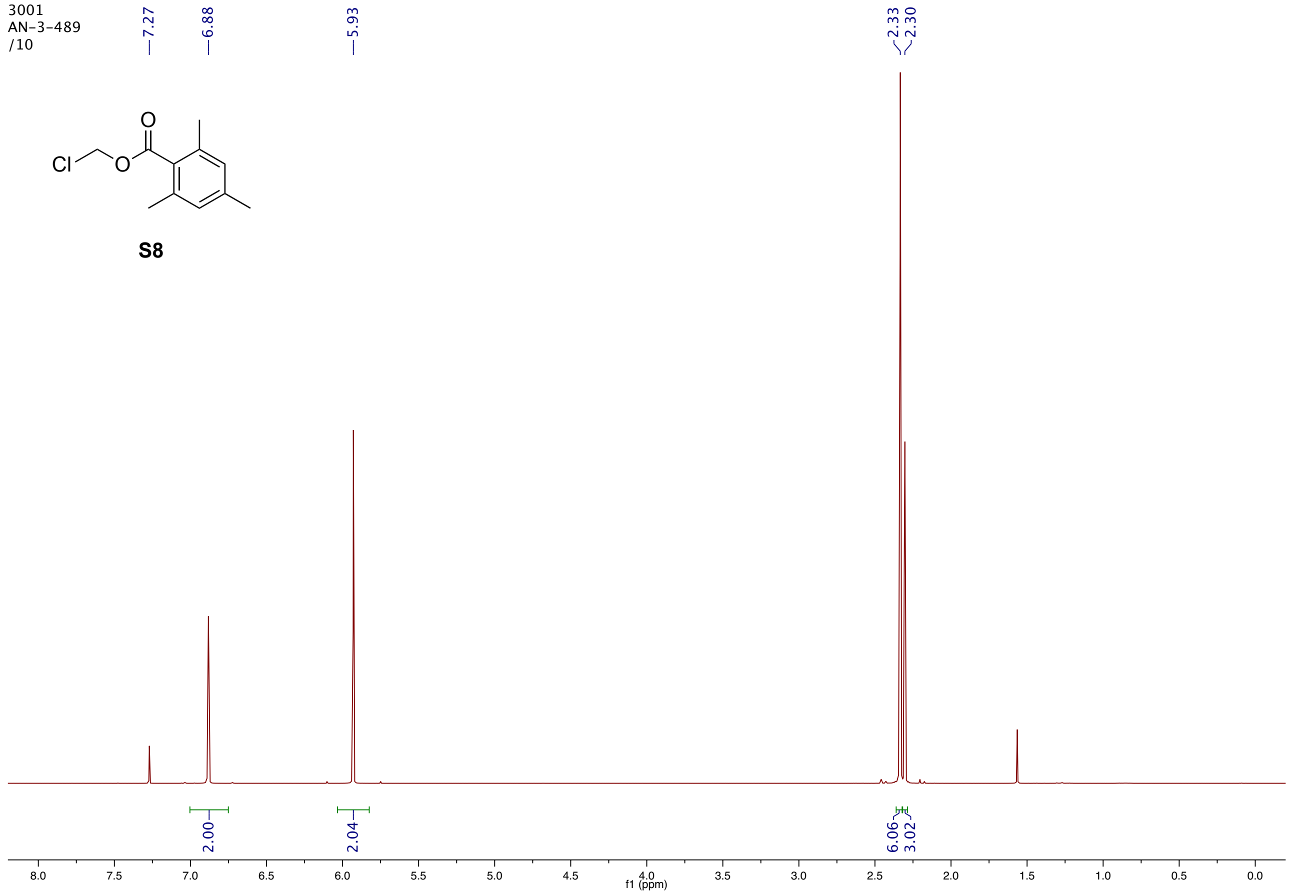

S8

^{13}C NMR (126 MHz, $CDCl_3$):

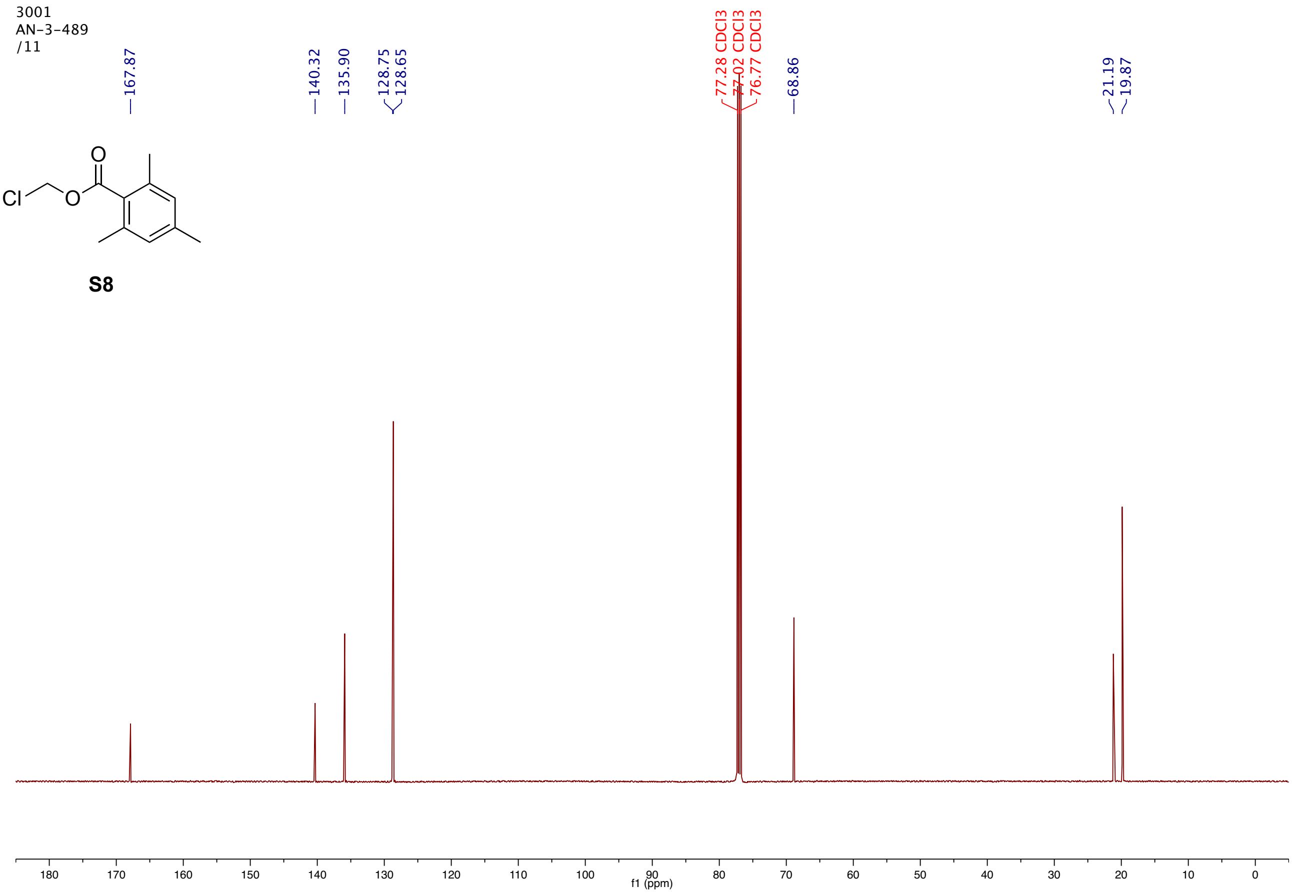

^{1}H NMR (400 MHz, $CDCl_3$):

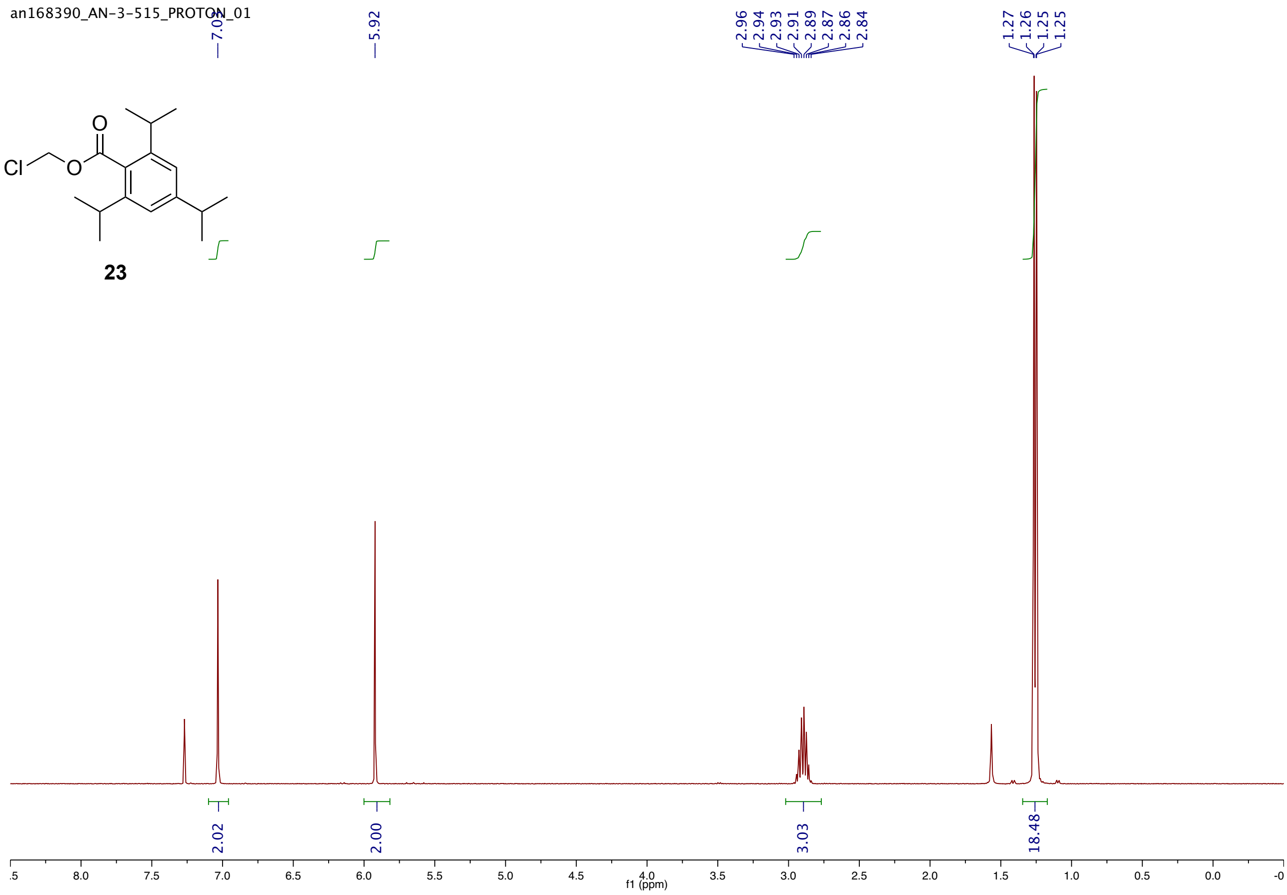

^{13}C NMR (101 MHz, $CDCl_3$):

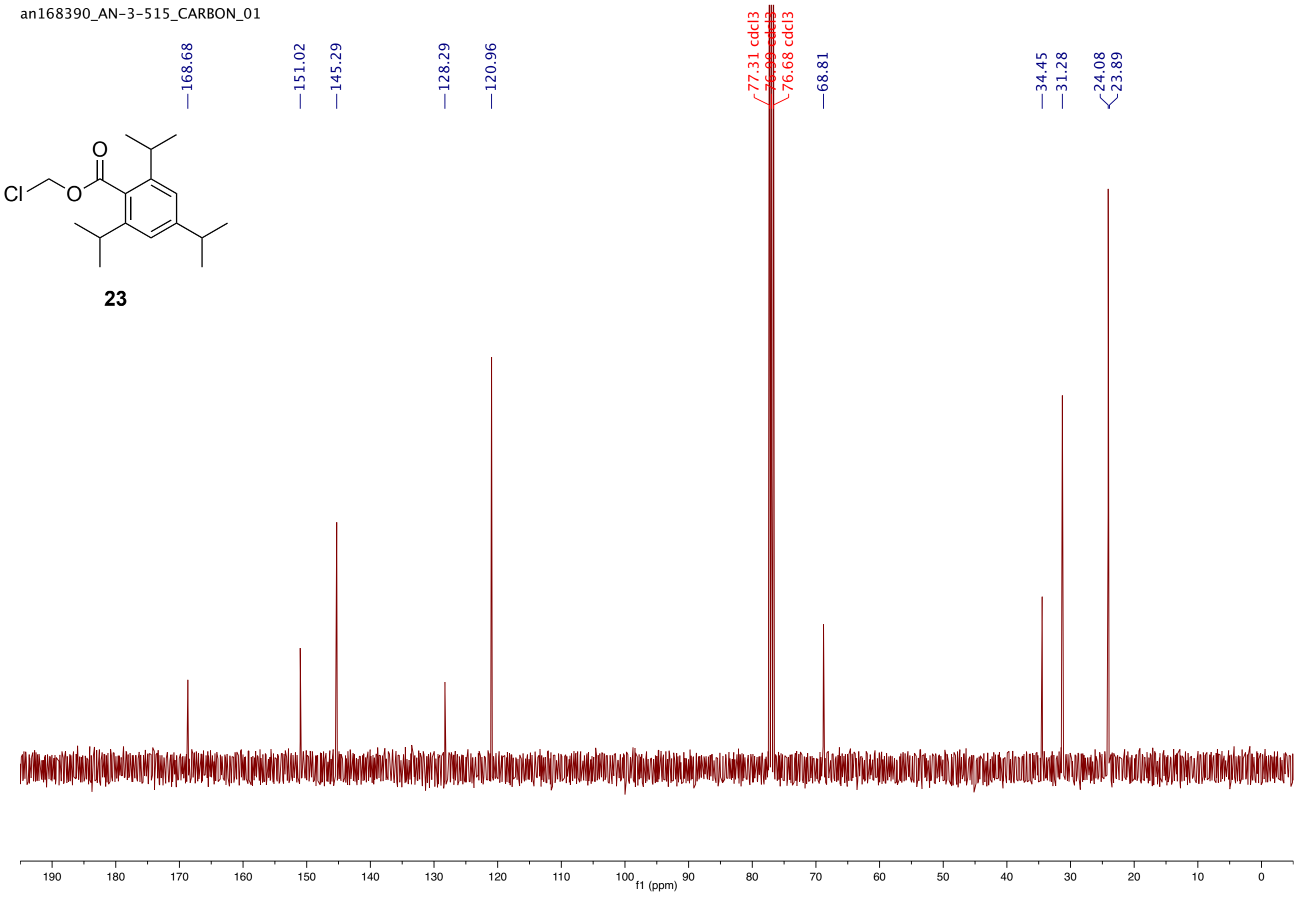

^{1}H NMR (500 MHz, $CDCl_3$):

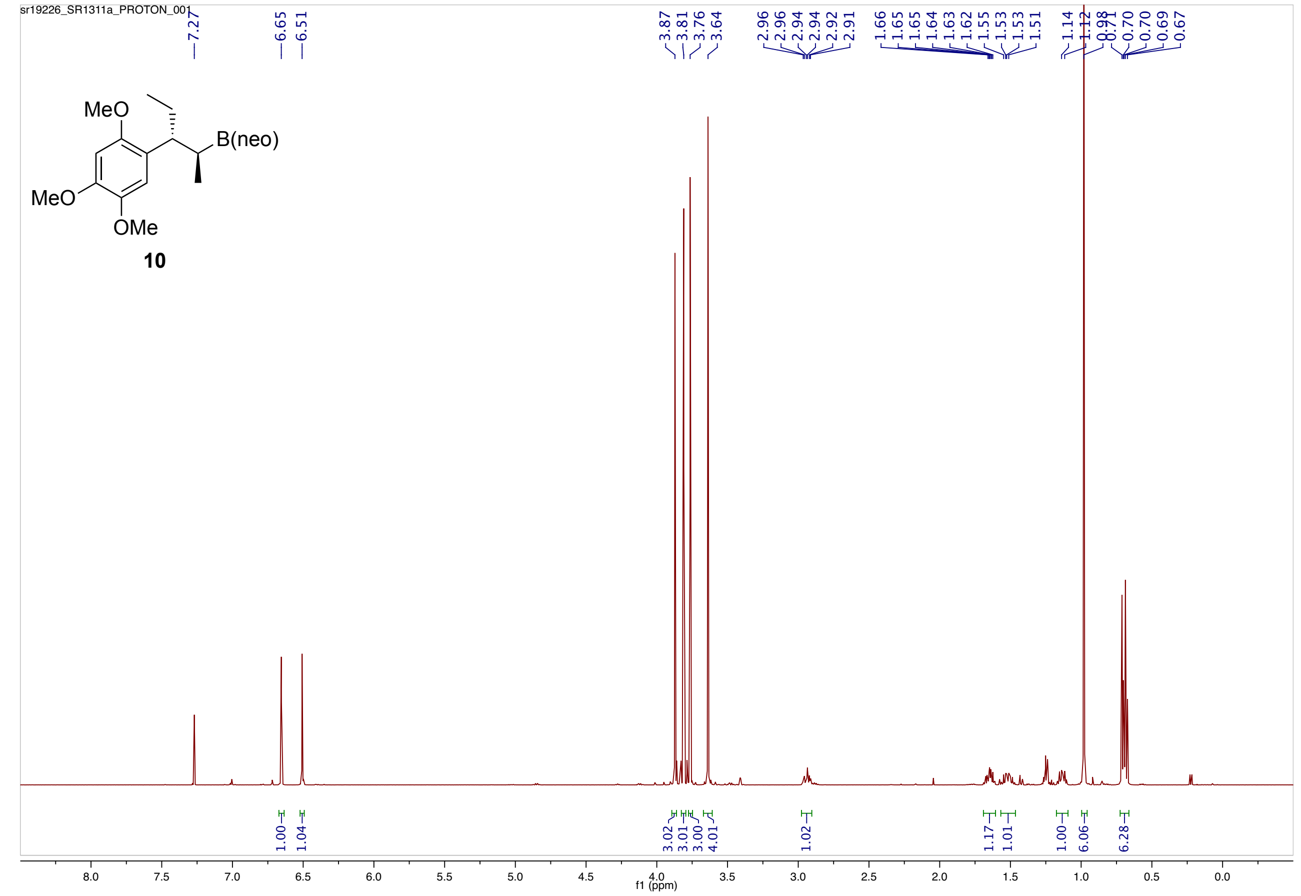

^{13}C NMR (126 MHz, $CDCl_3$):

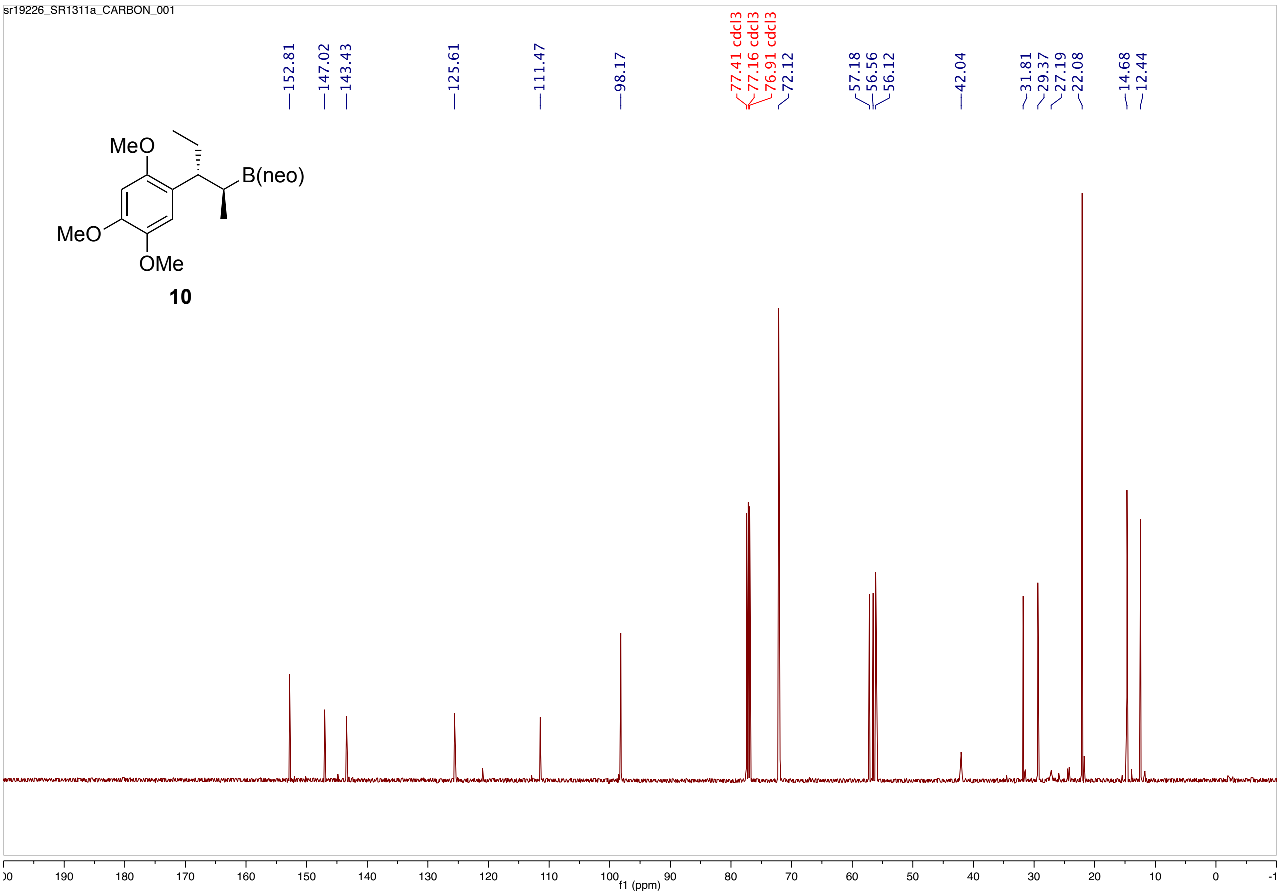

^{1}H NMR (500 MHz, $CDCl_3$):

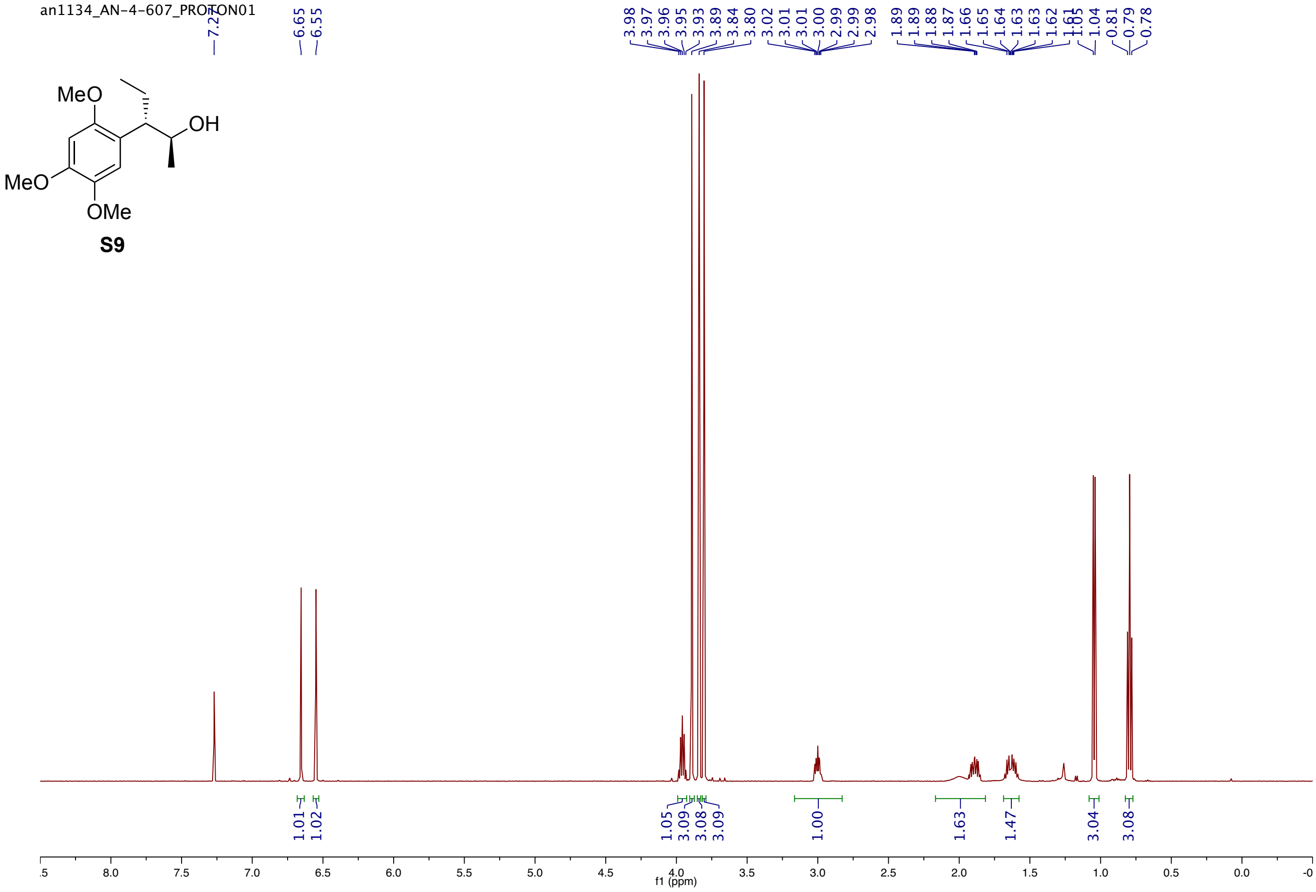

^{13}C NMR (126 MHz, $CDCl_3$):

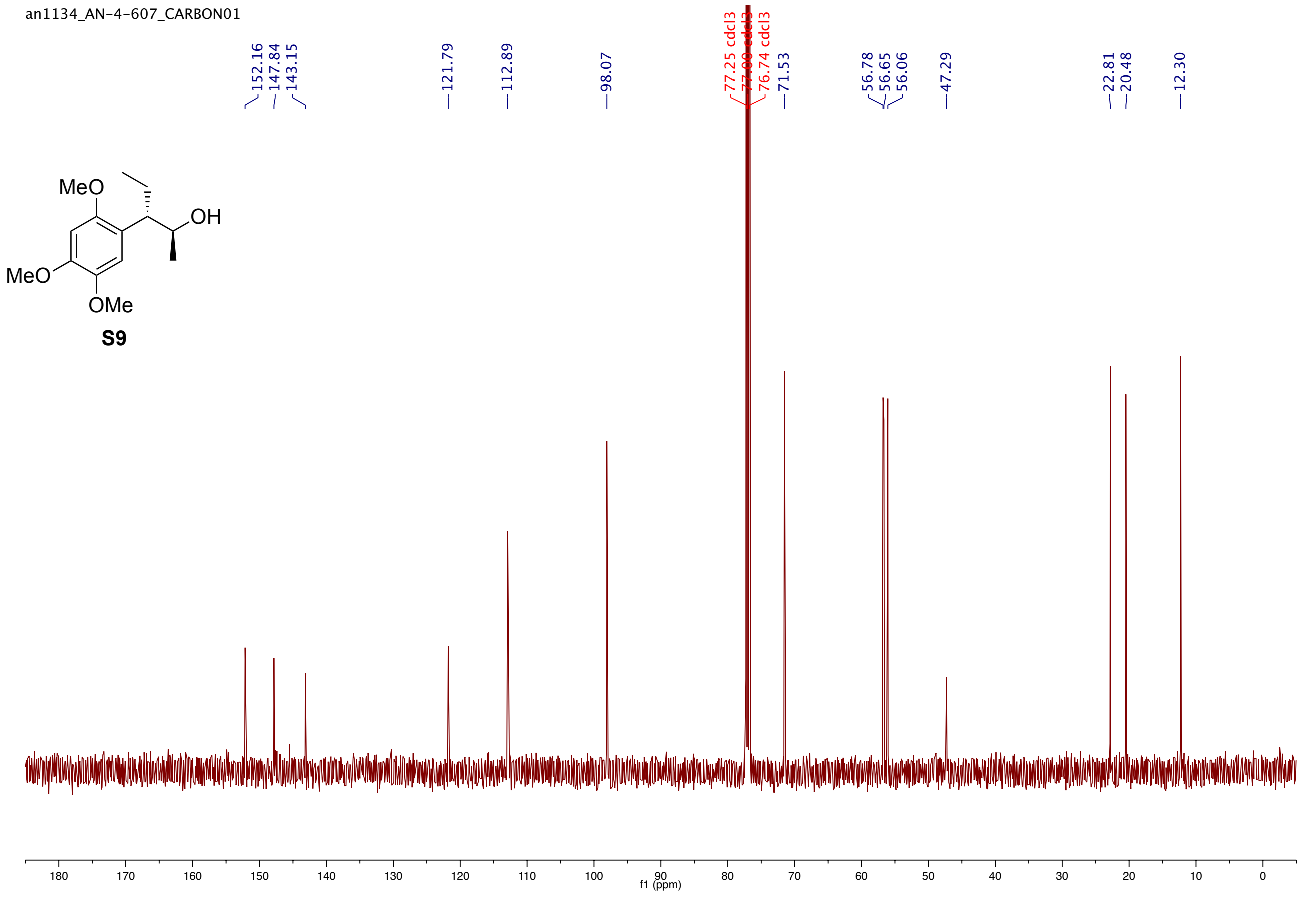

^{1}H NMR (400 MHz, $CDCl_3$):

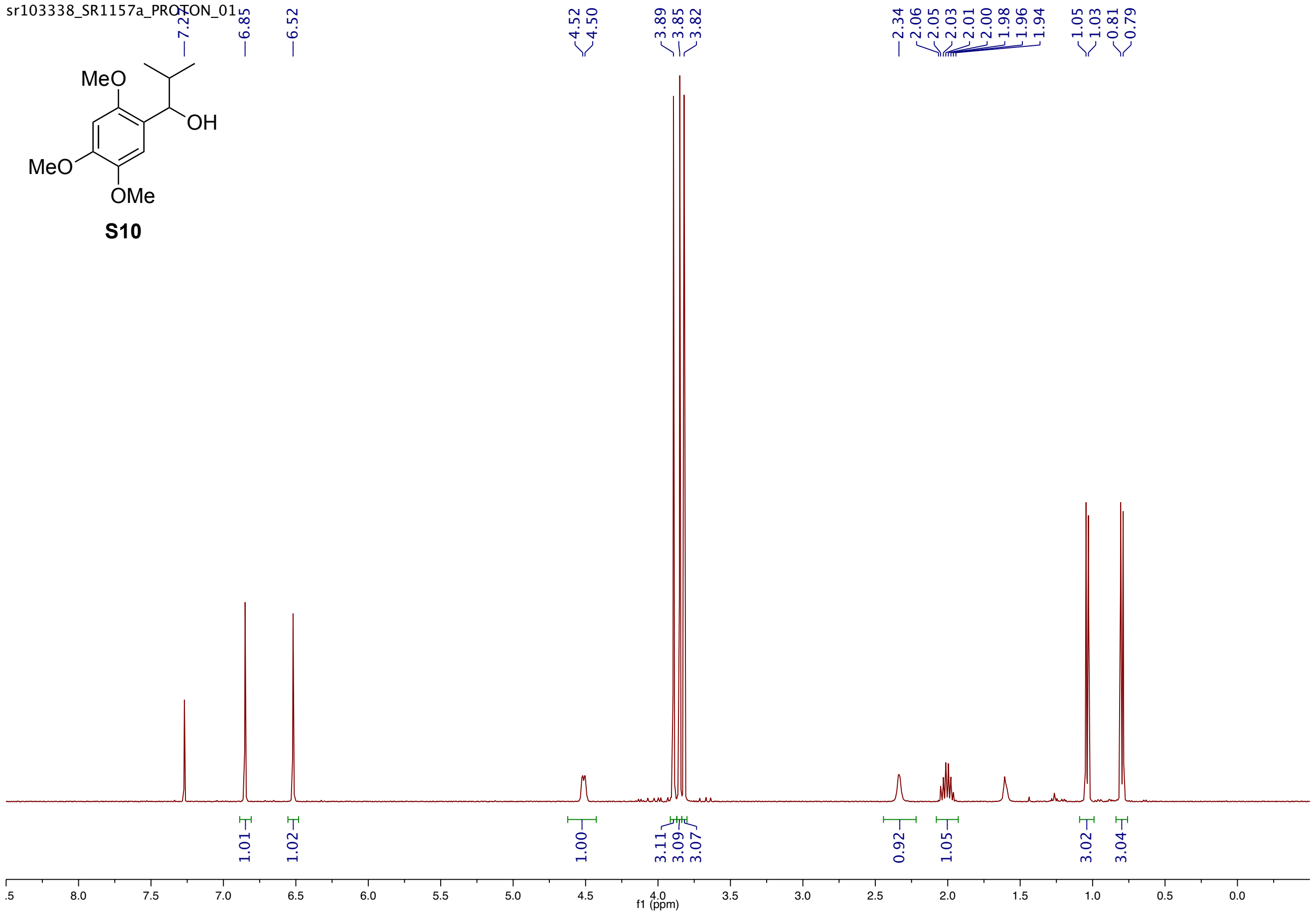

^{13}C NMR (101 MHz, $CDCl_3$):

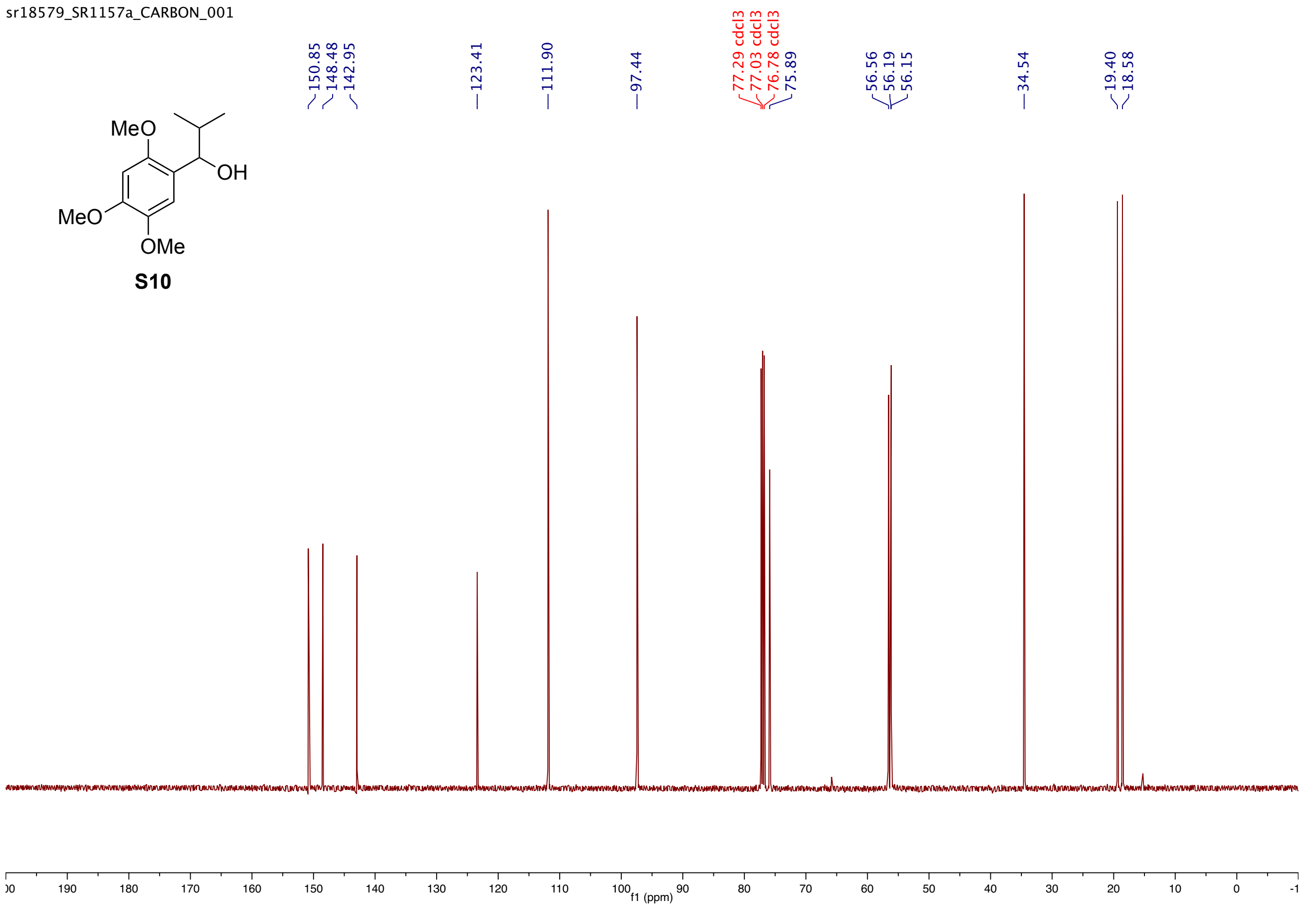

^{1}H NMR (500 MHz, $CDCl_3$):

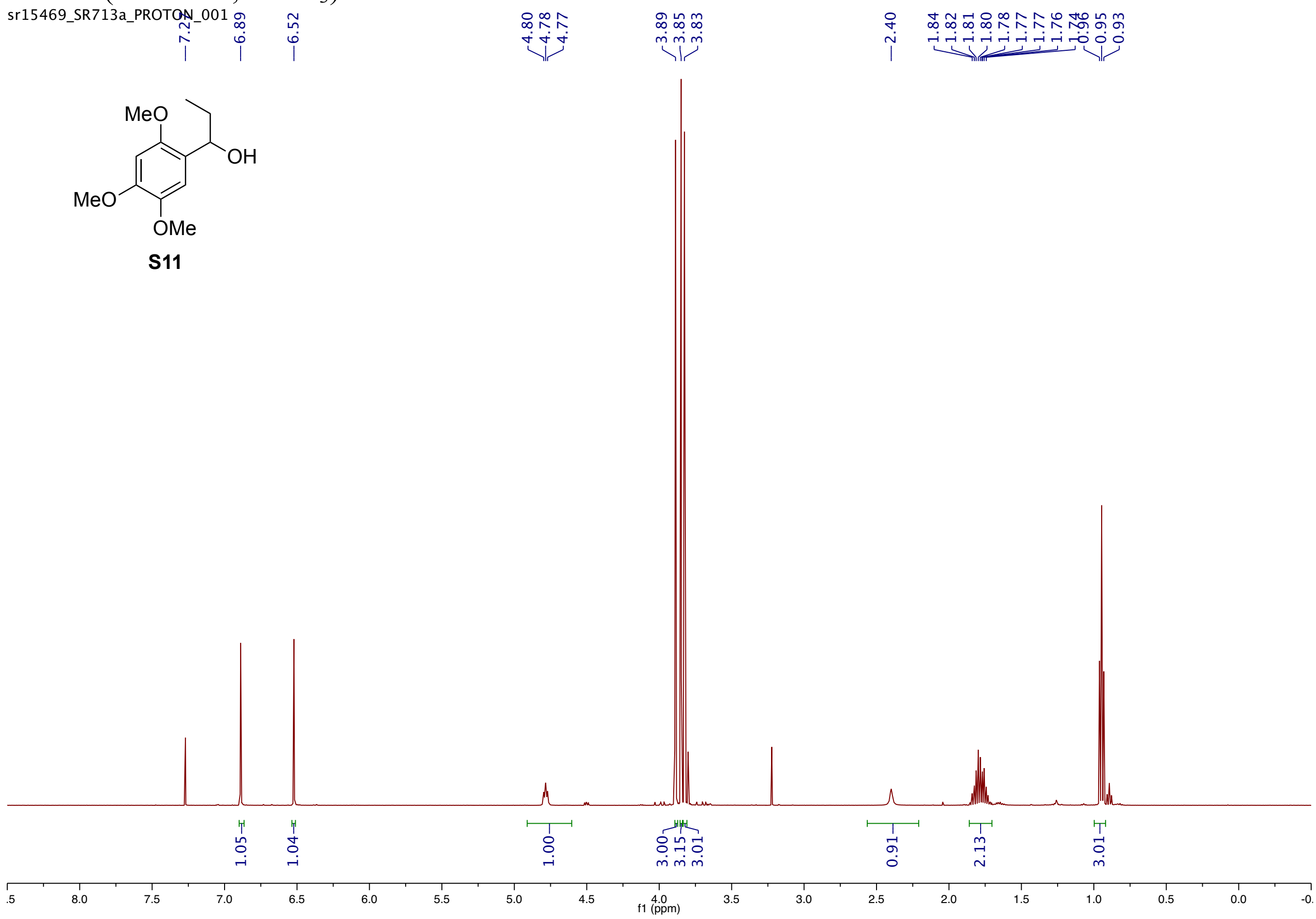

^{13}C NMR (101 MHz, $CDCl_3$):

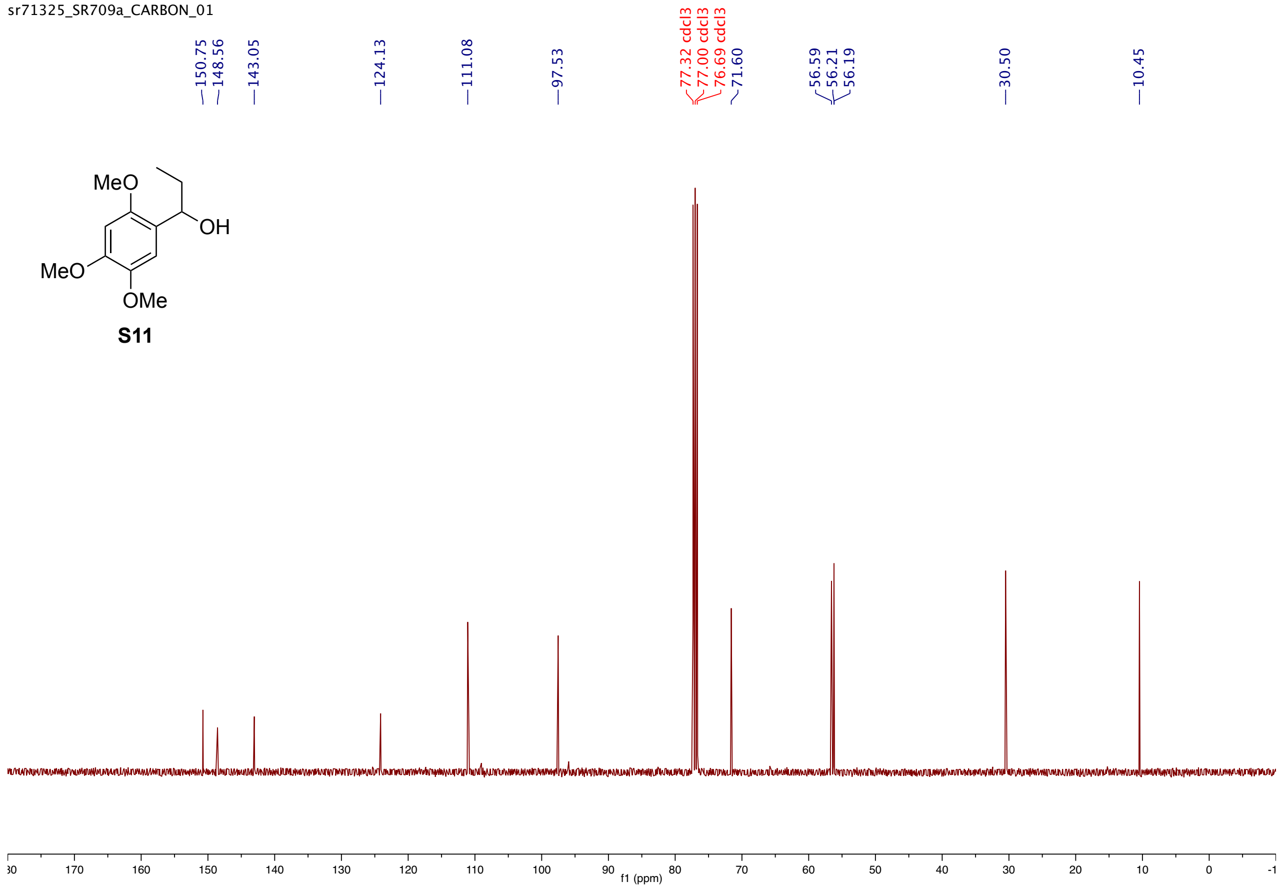

^{1}H NMR (500 MHz, $CDCl_3$):

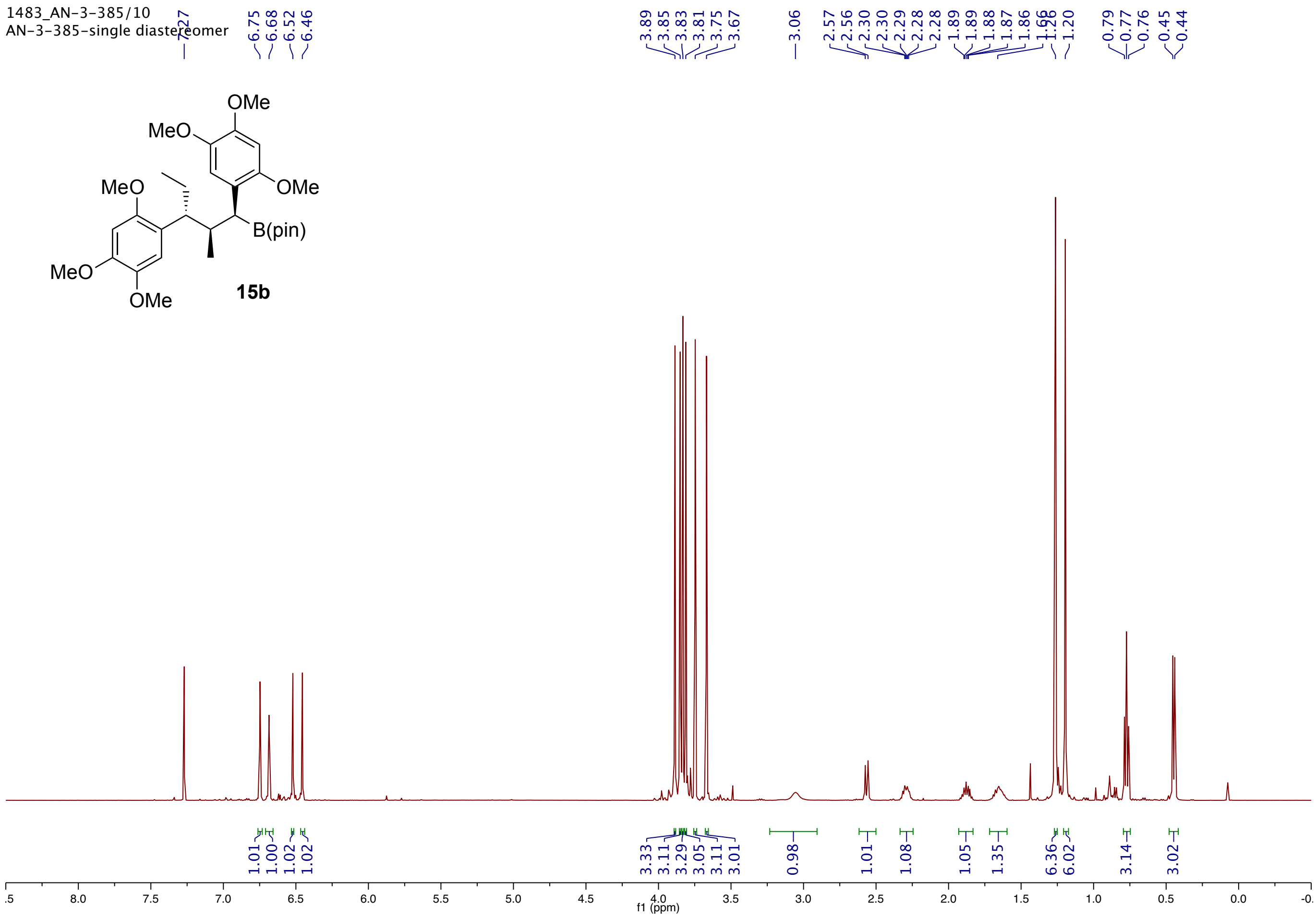

^{13}C NMR (126 MHz, $CDCl_3$):

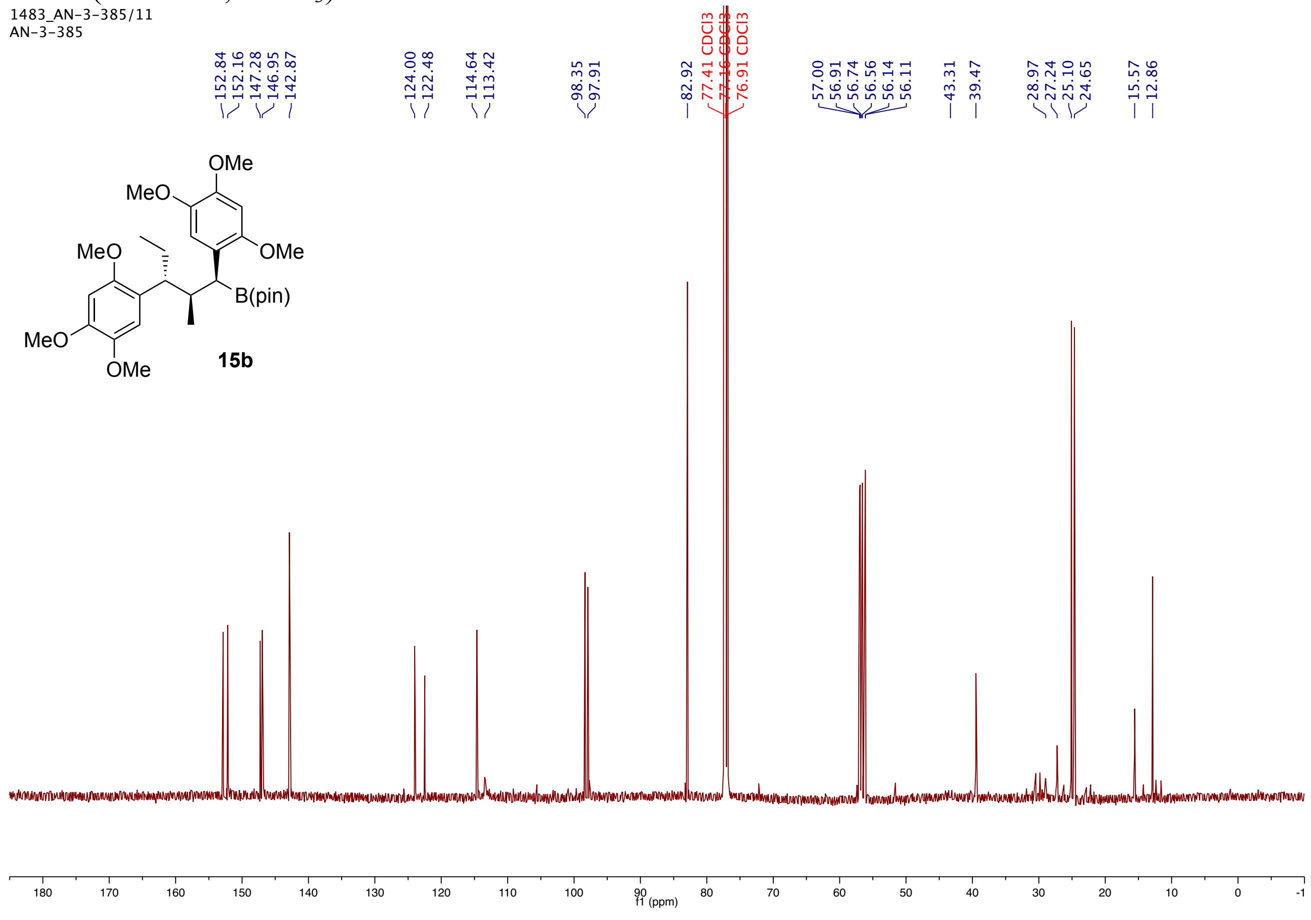

^{1}H NMR (400 MHz, $CDCl_3$):

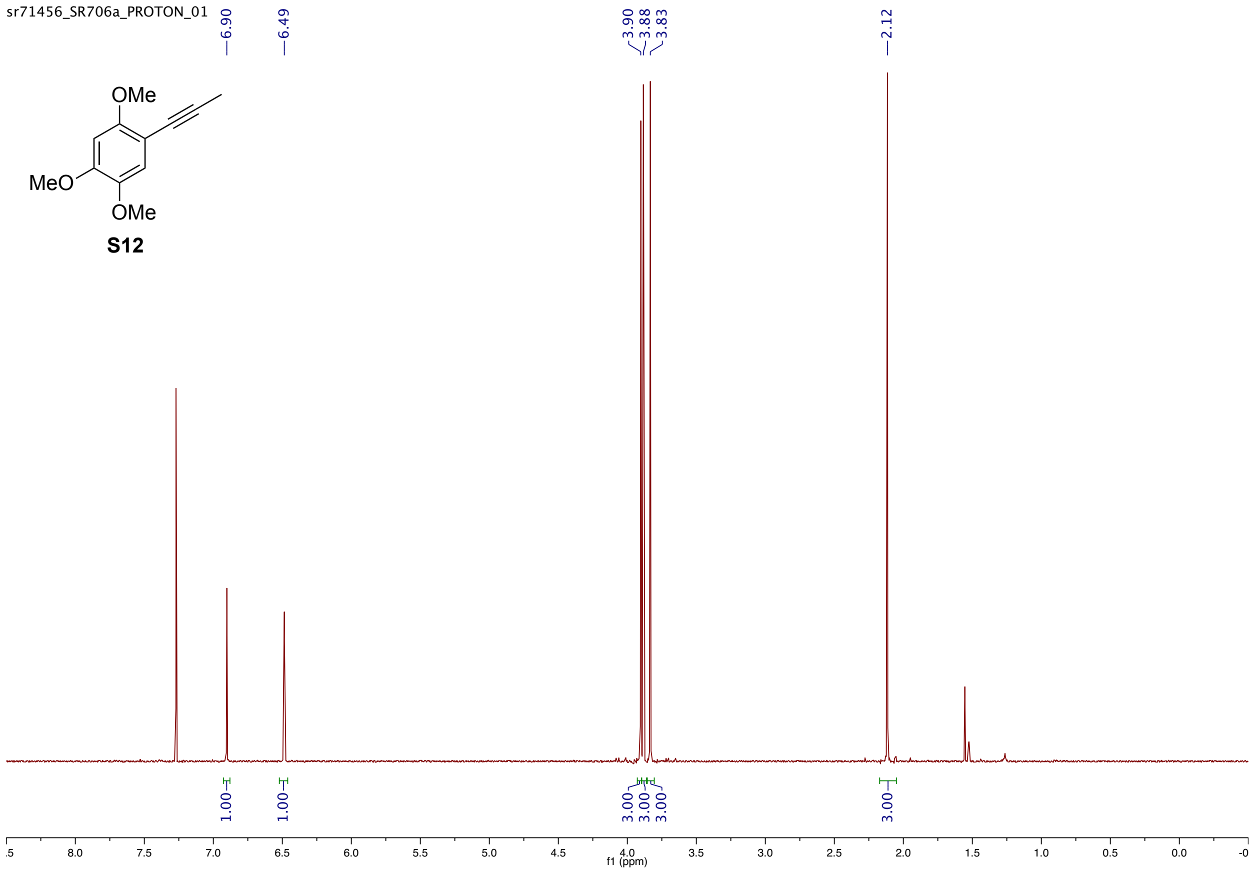

^{13}C NMR (126 MHz, $CDCl_3$):

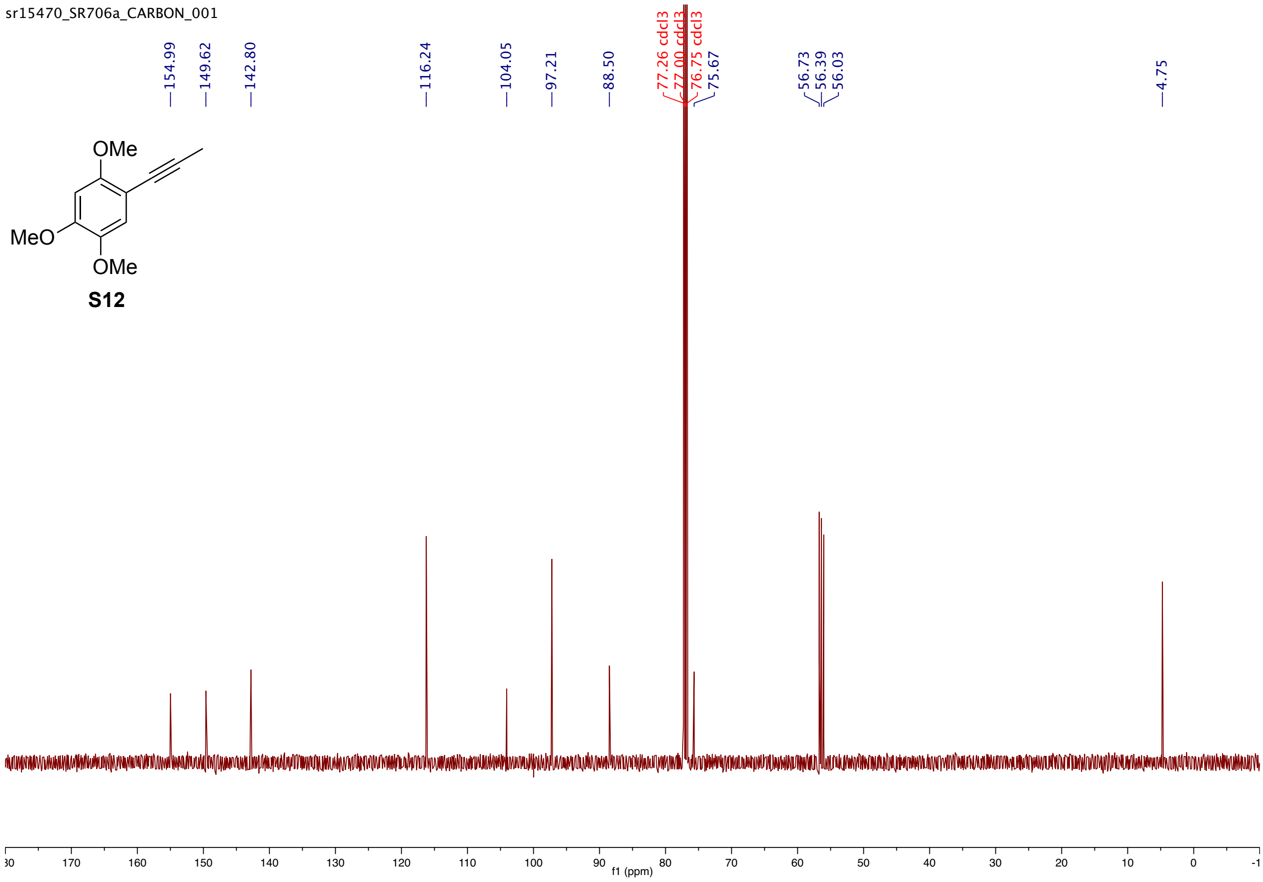

^{1}H NMR (400 MHz, $CDCl_3$):

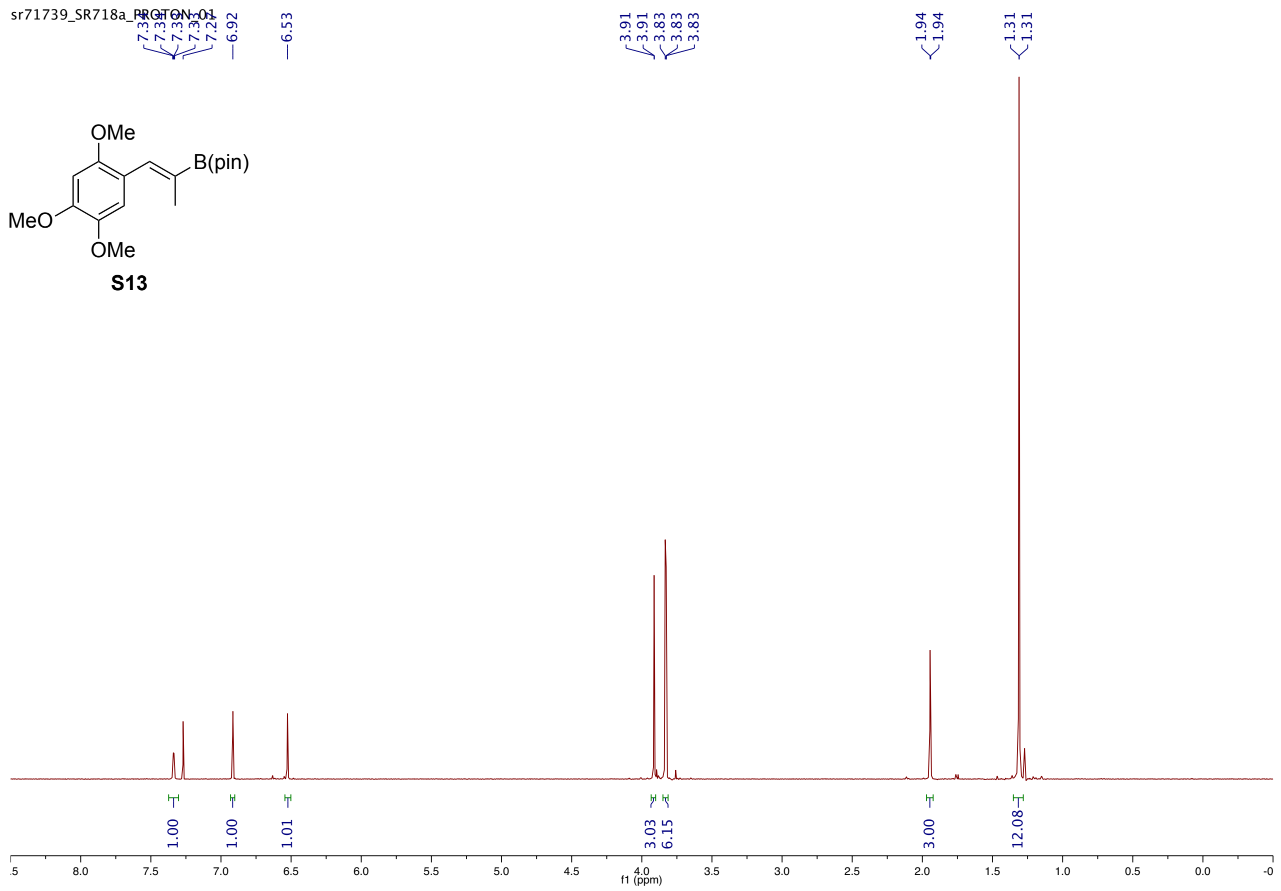

^{13}C NMR (126 MHz, $CDCl_3$):

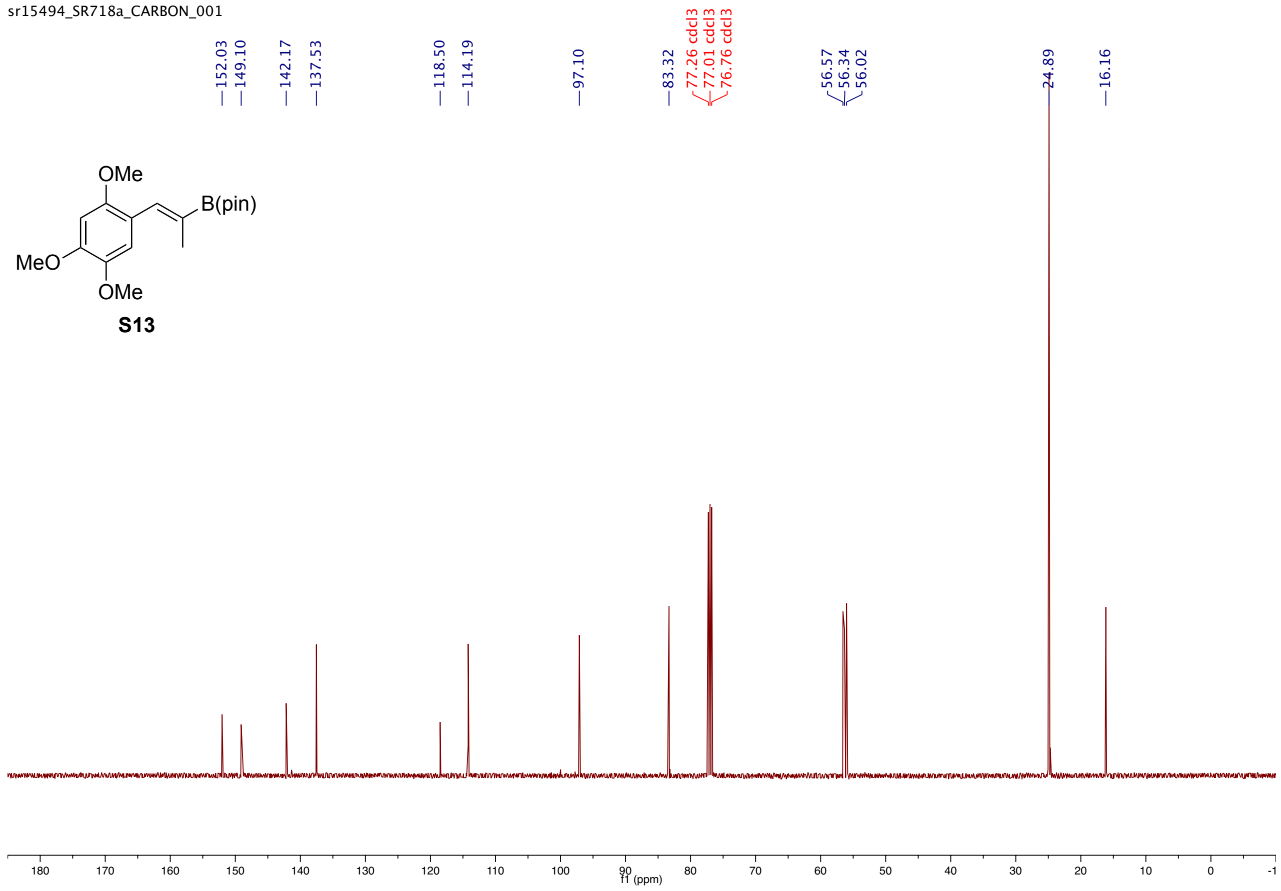

^{1}H NMR (500 MHz, $CDCl_3$):

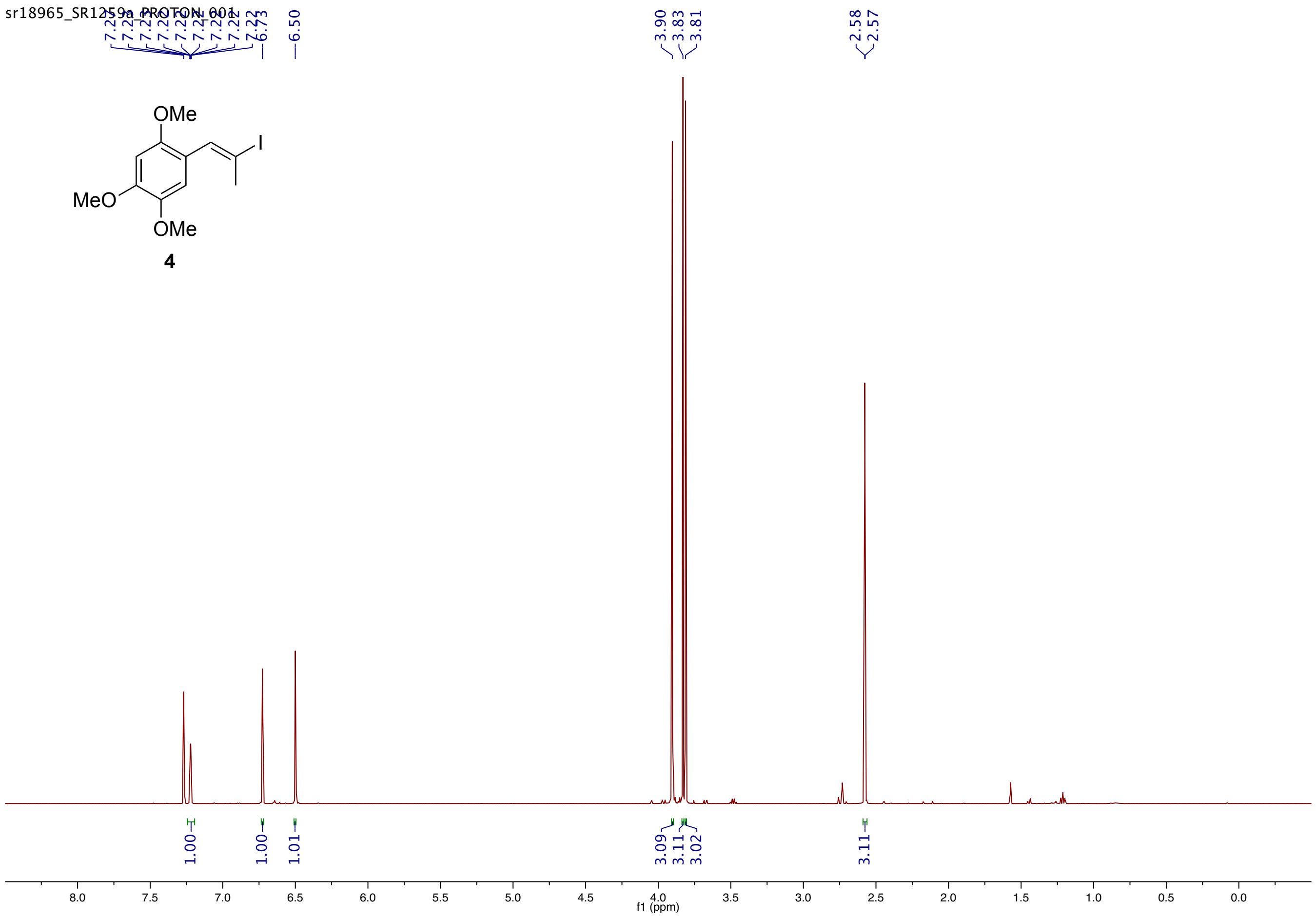

^{13}C NMR (126 MHz, $CDCl_3$):

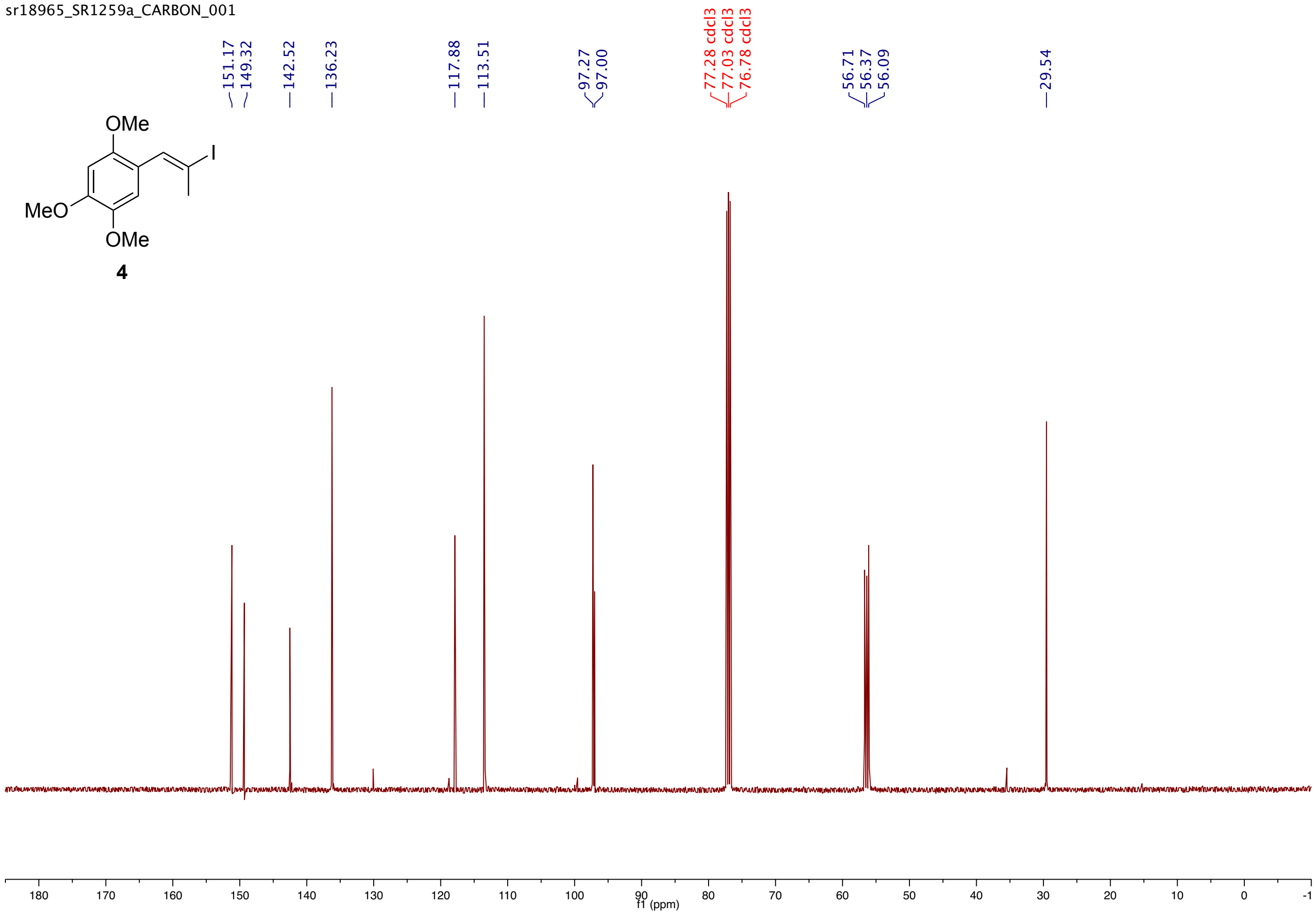

^{1}H NMR (500 MHz, $CDCl_3$):

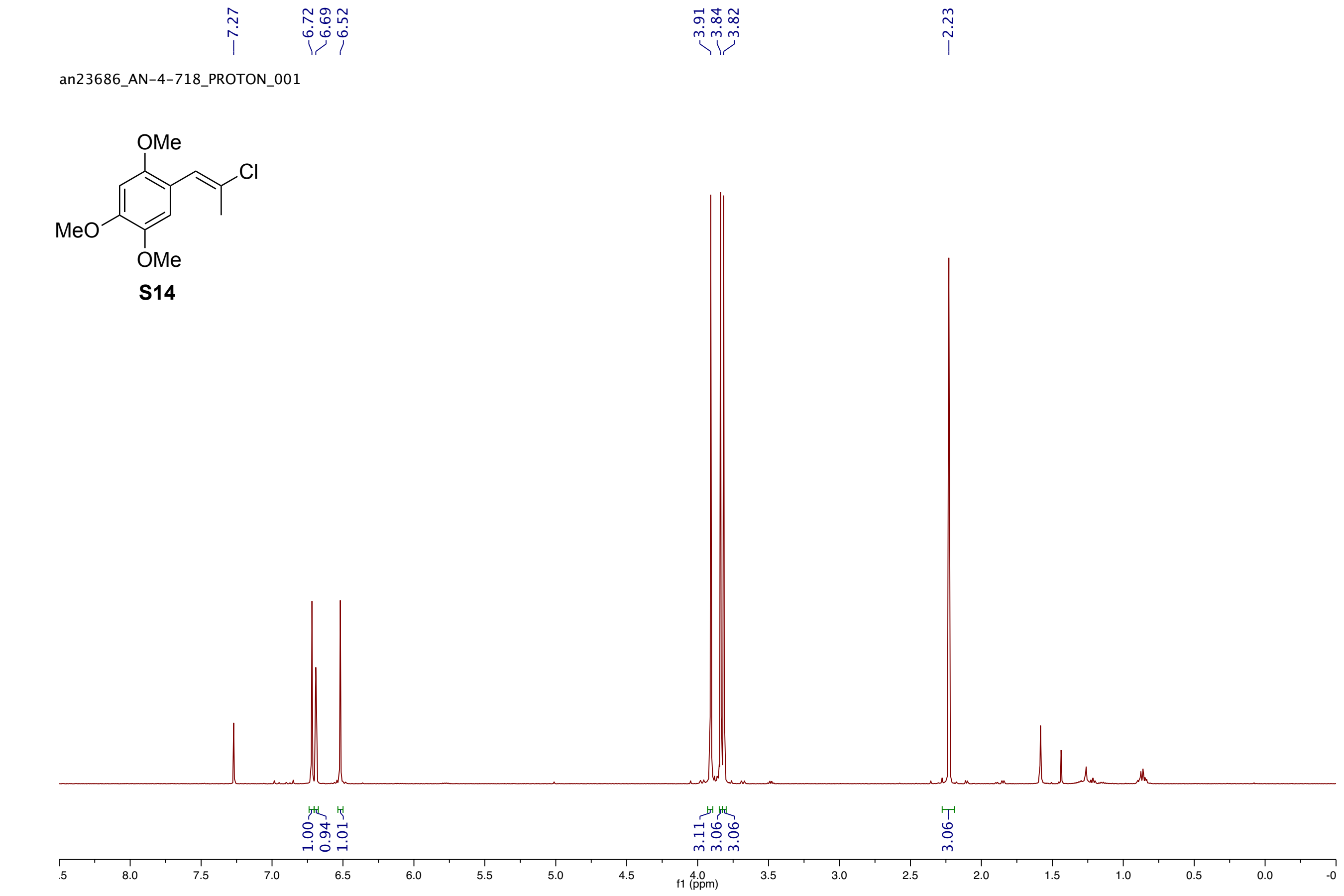

^{13}C NMR (126 MHz, $CDCl_3$):

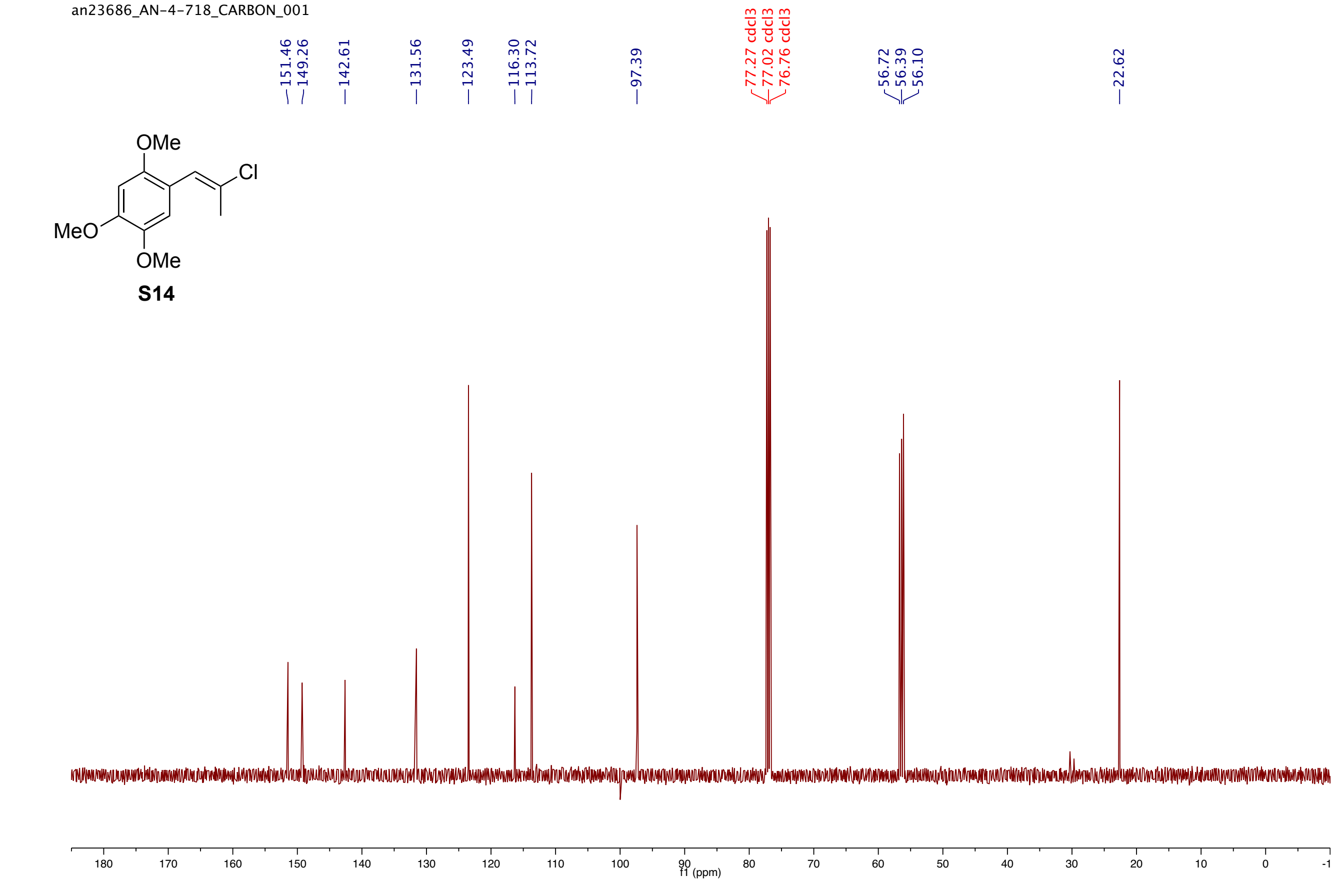

^{1}H NMR (400 MHz, $CDCl_3$):

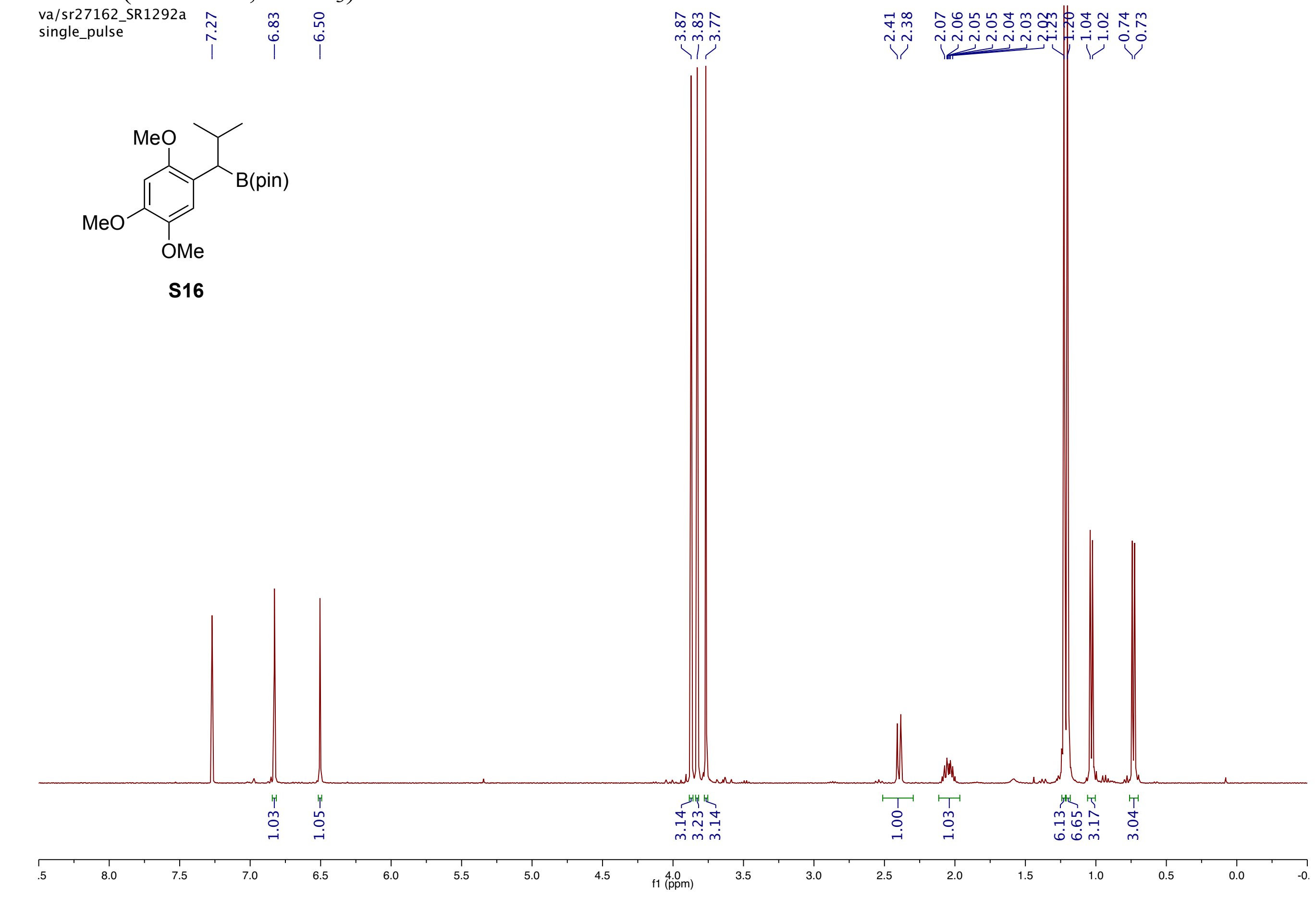

^{13}C NMR (126 MHz, $CDCl_3$):

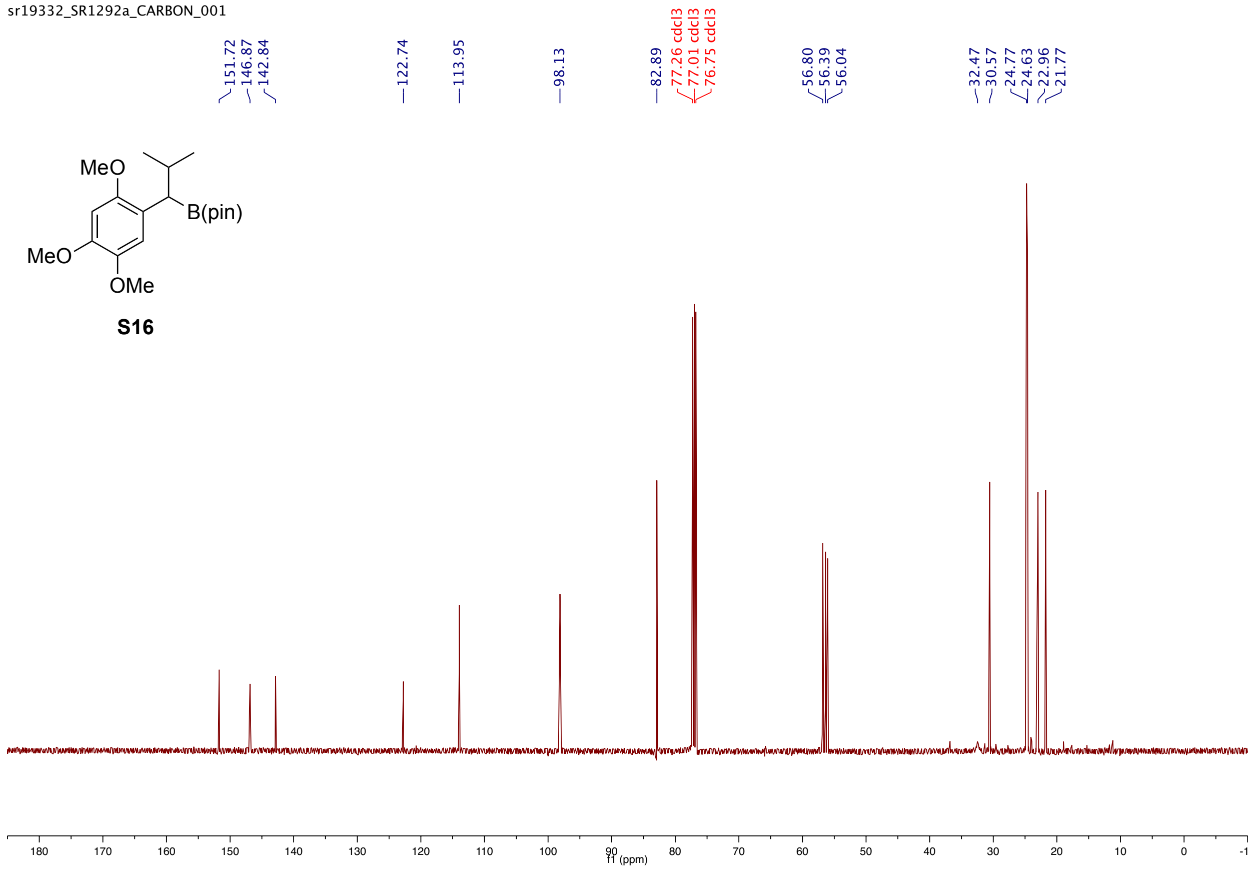

^{1}H NMR (500 MHz, DMSO-d_6):

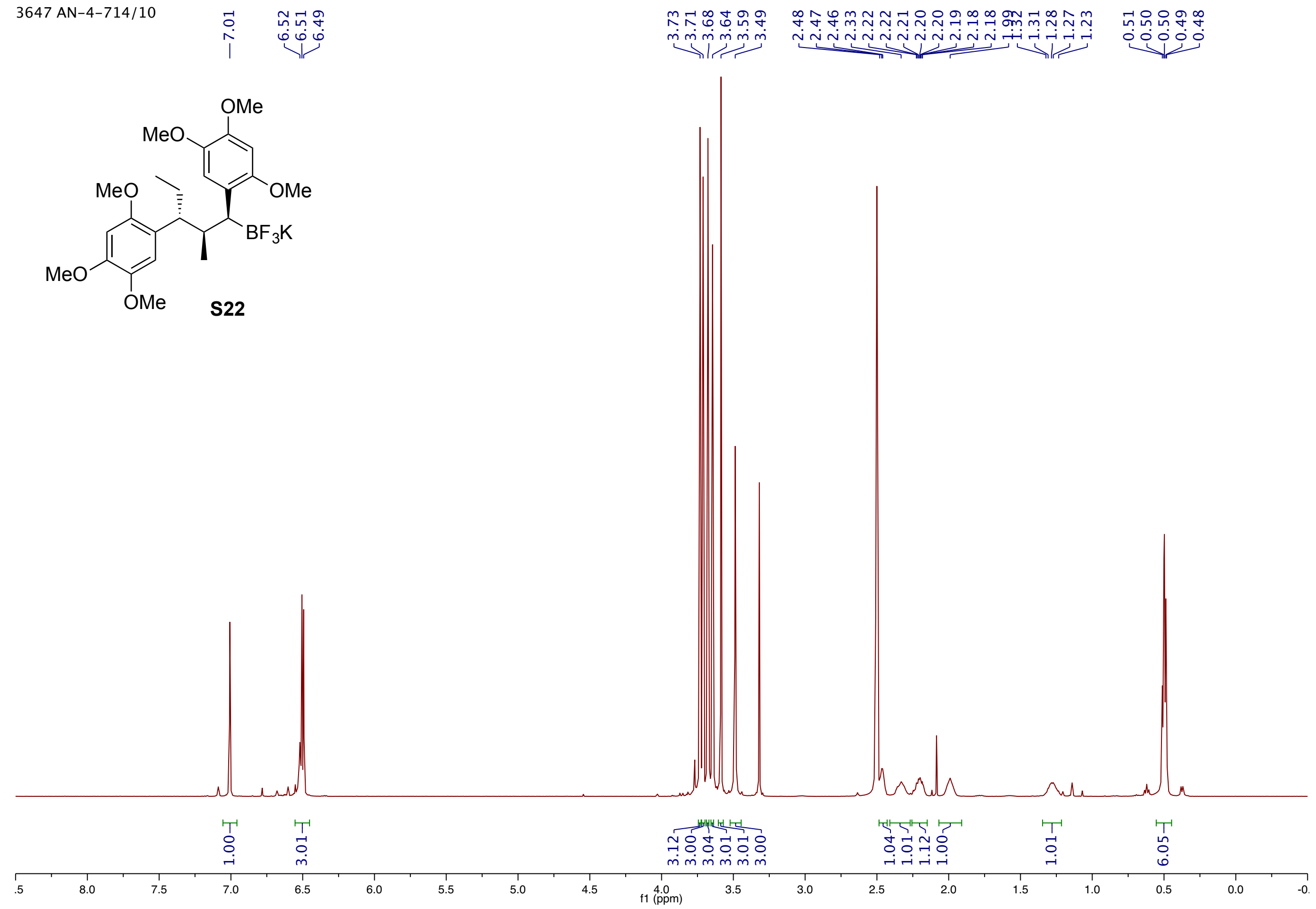

^{13}C NMR (126 MHz, DMSO-d_6):

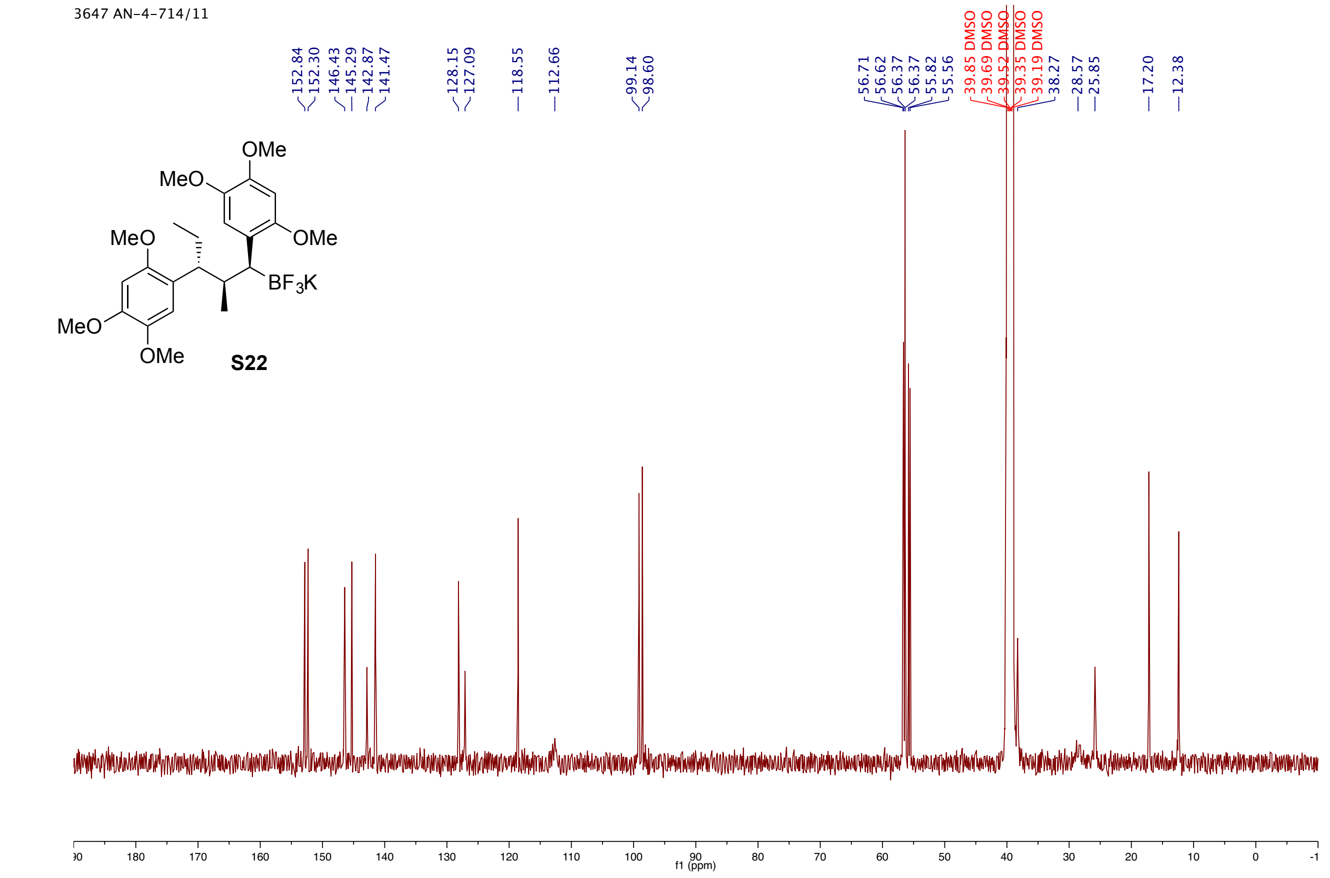

^{1}H NMR (500 MHz, $CDCl_3$):

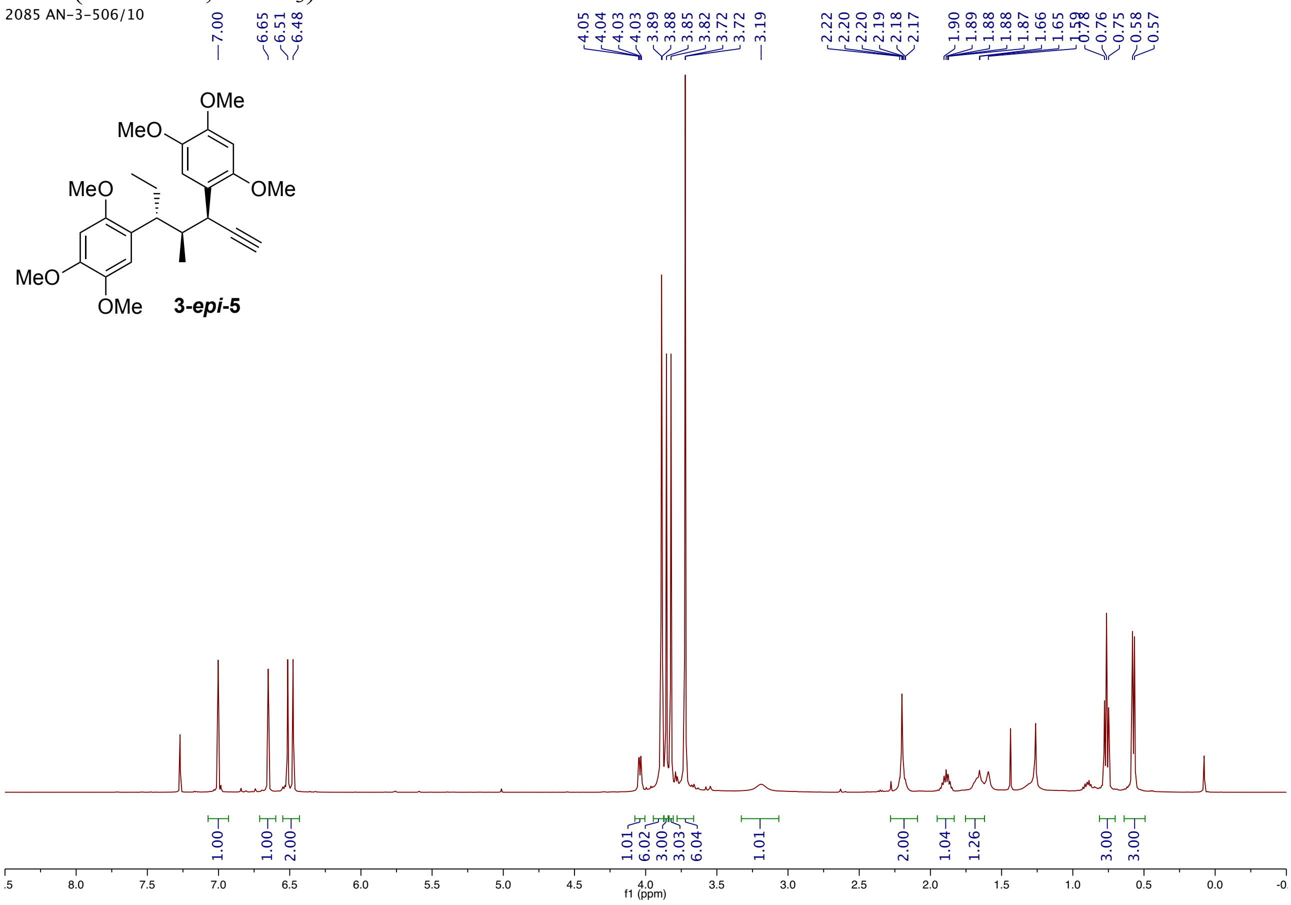

^{13}C NMR (126 MHz, $CDCl_3$):

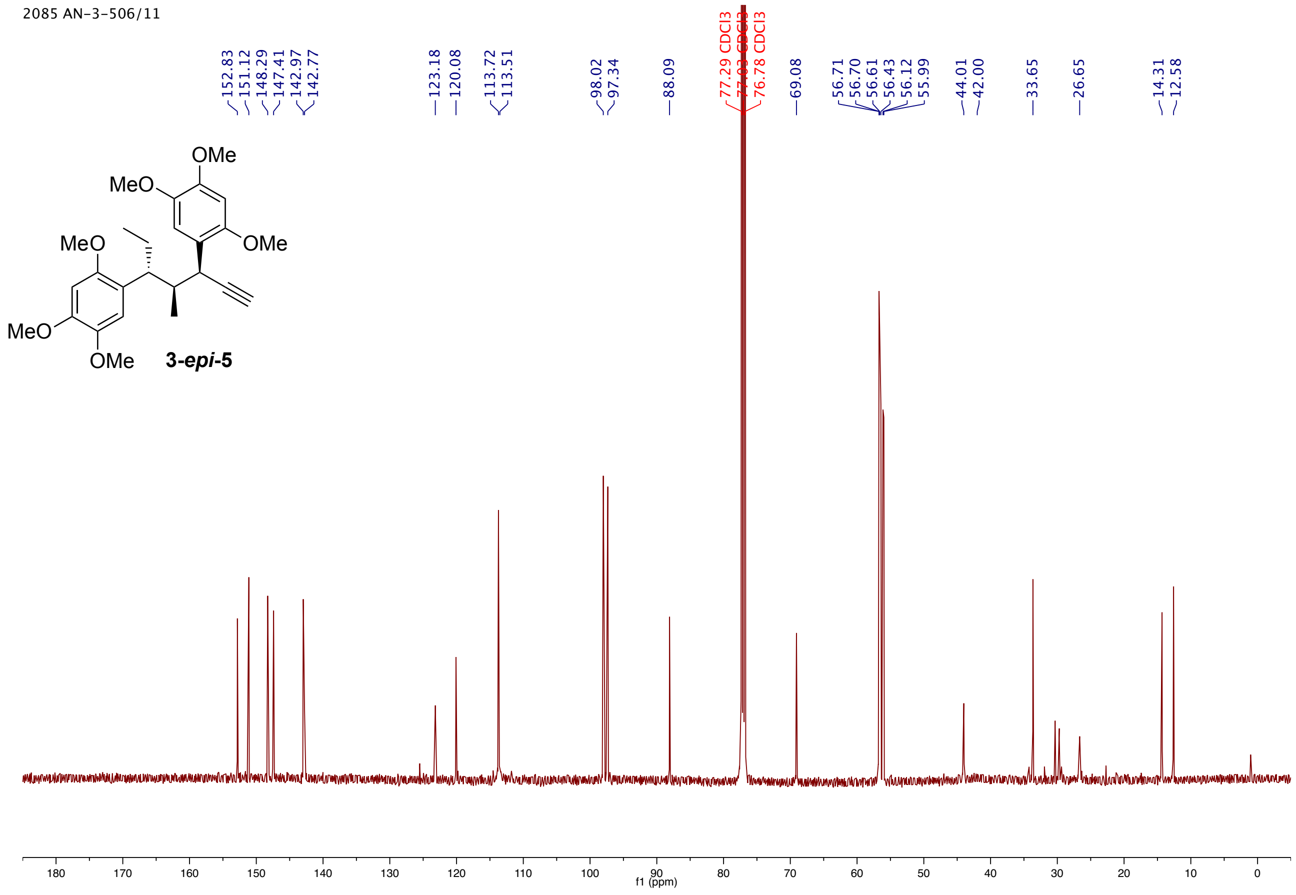

^{1}H NMR (500 MHz, $CDCl_3$):

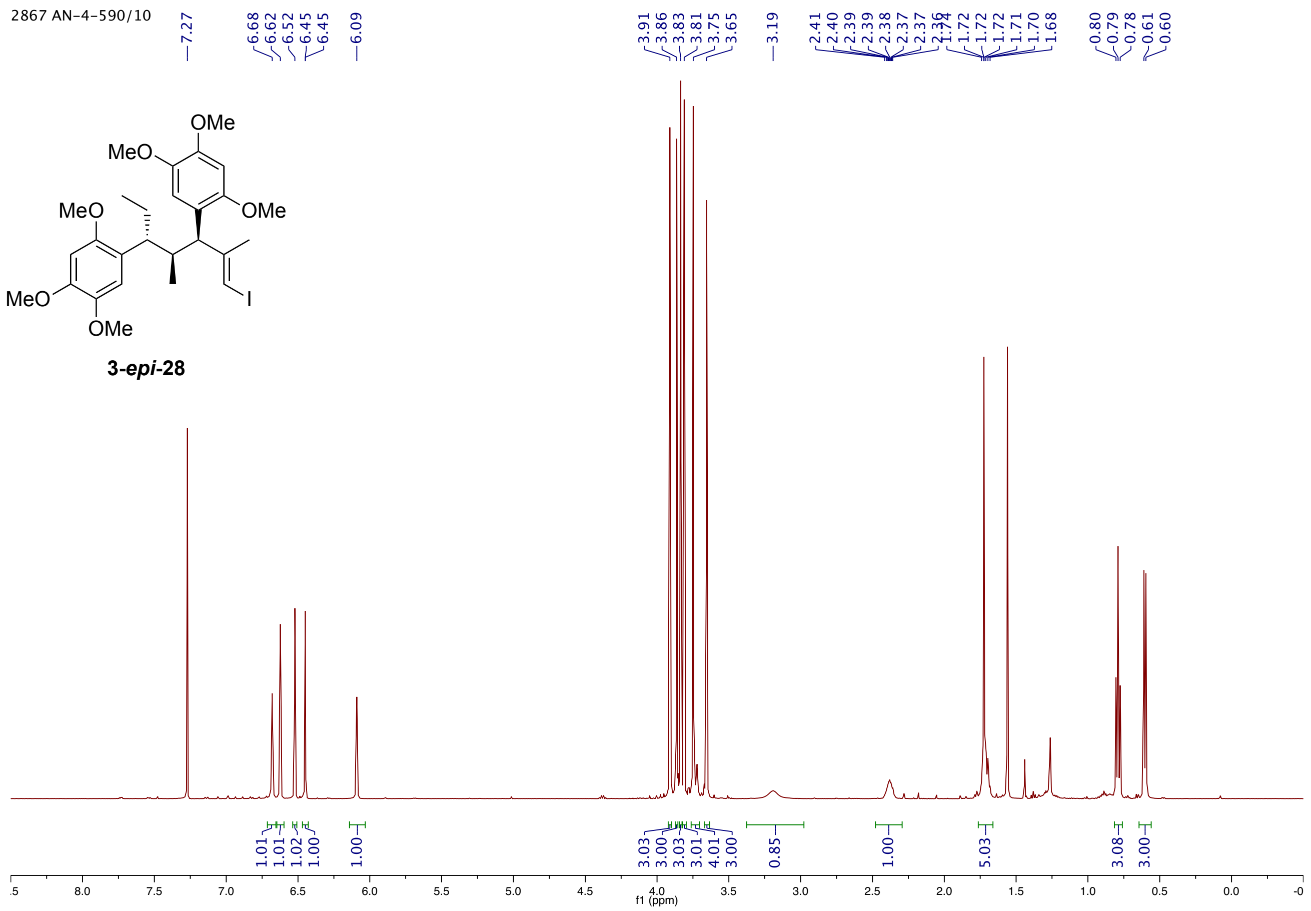

^{13}C NMR (126 MHz, $CDCl_3$):

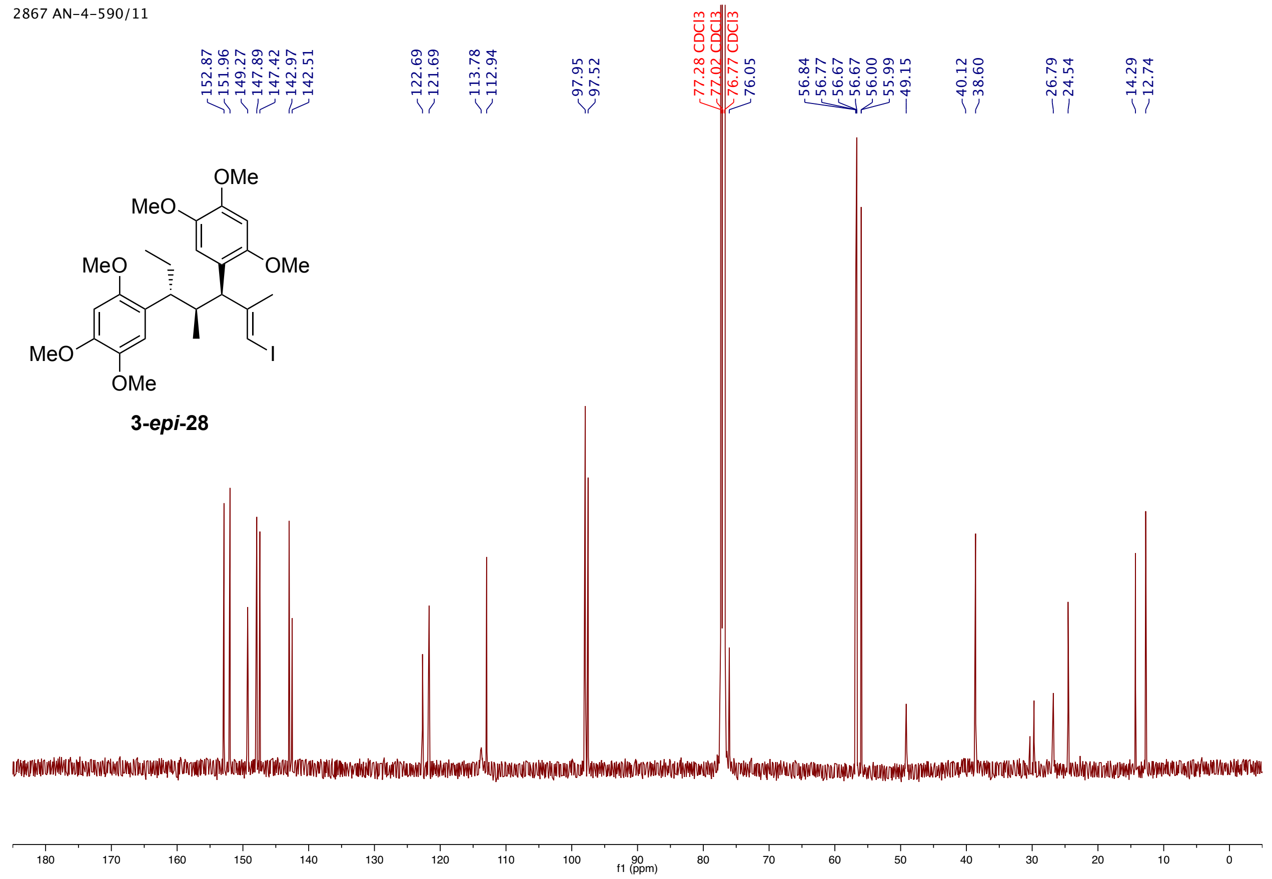

^{1}H NMR (500 MHz, $CDCl_3$):

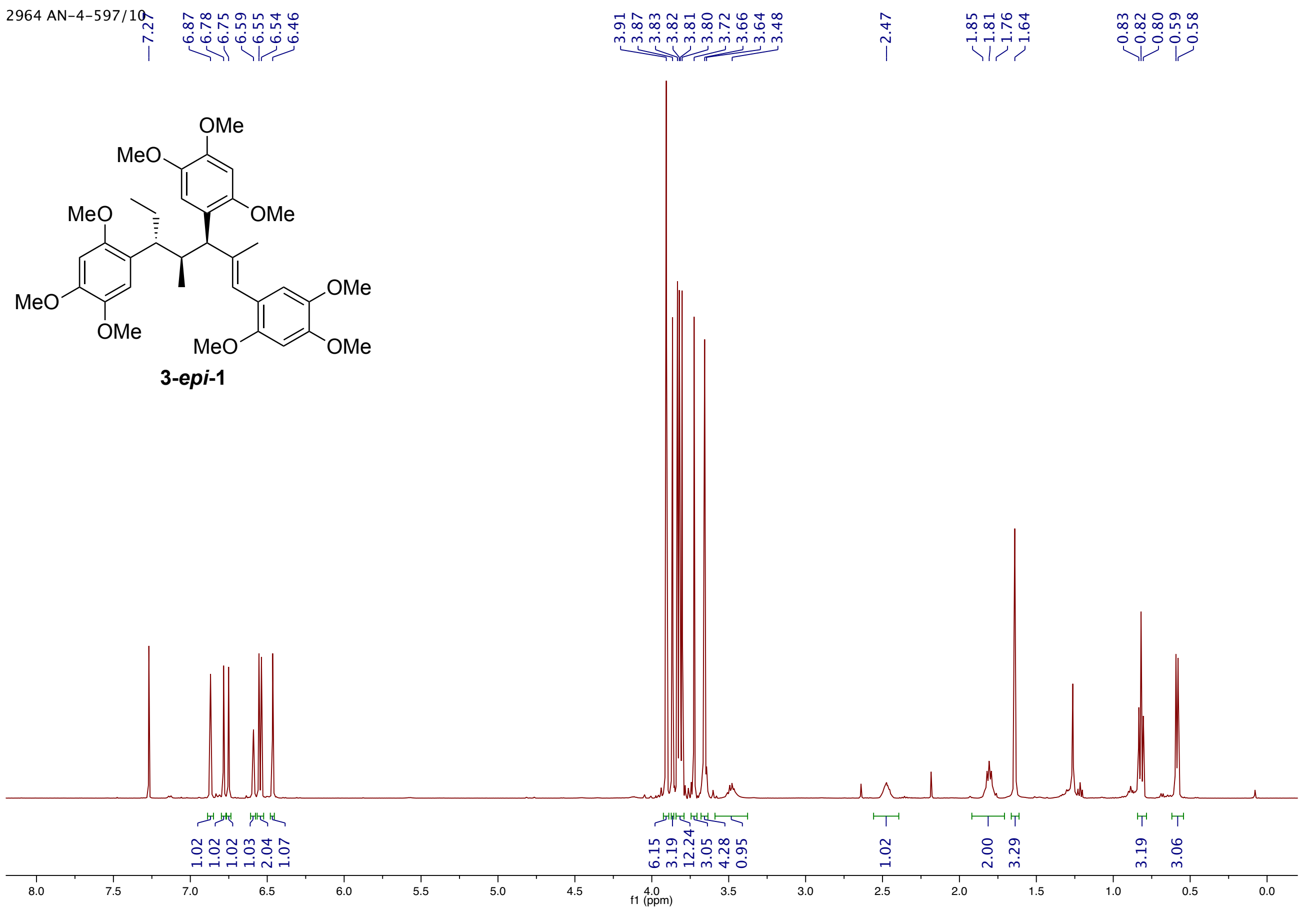

^{13}C NMR (126 MHz, $CDCl_3$):

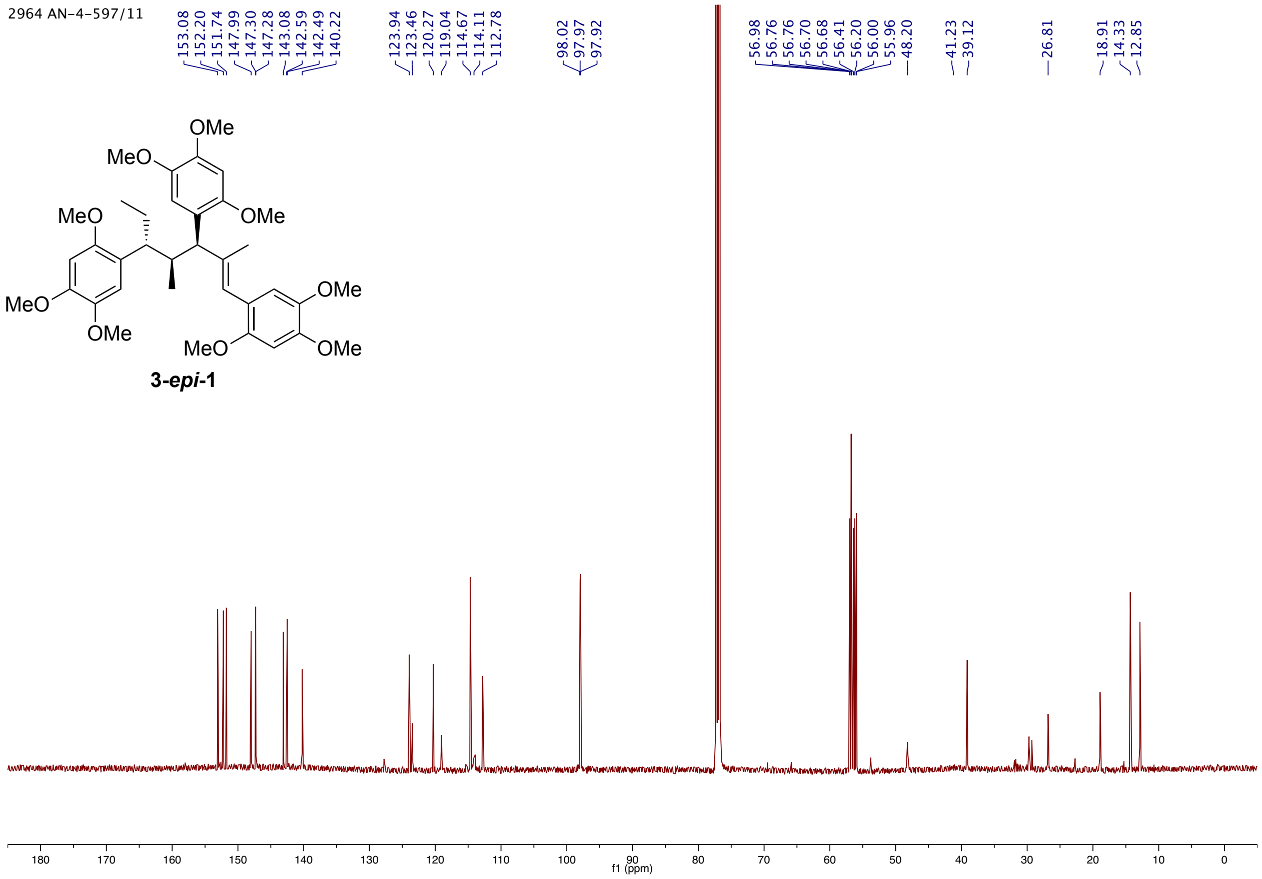

^{1}H NMR (500 MHz, CD_3OD):

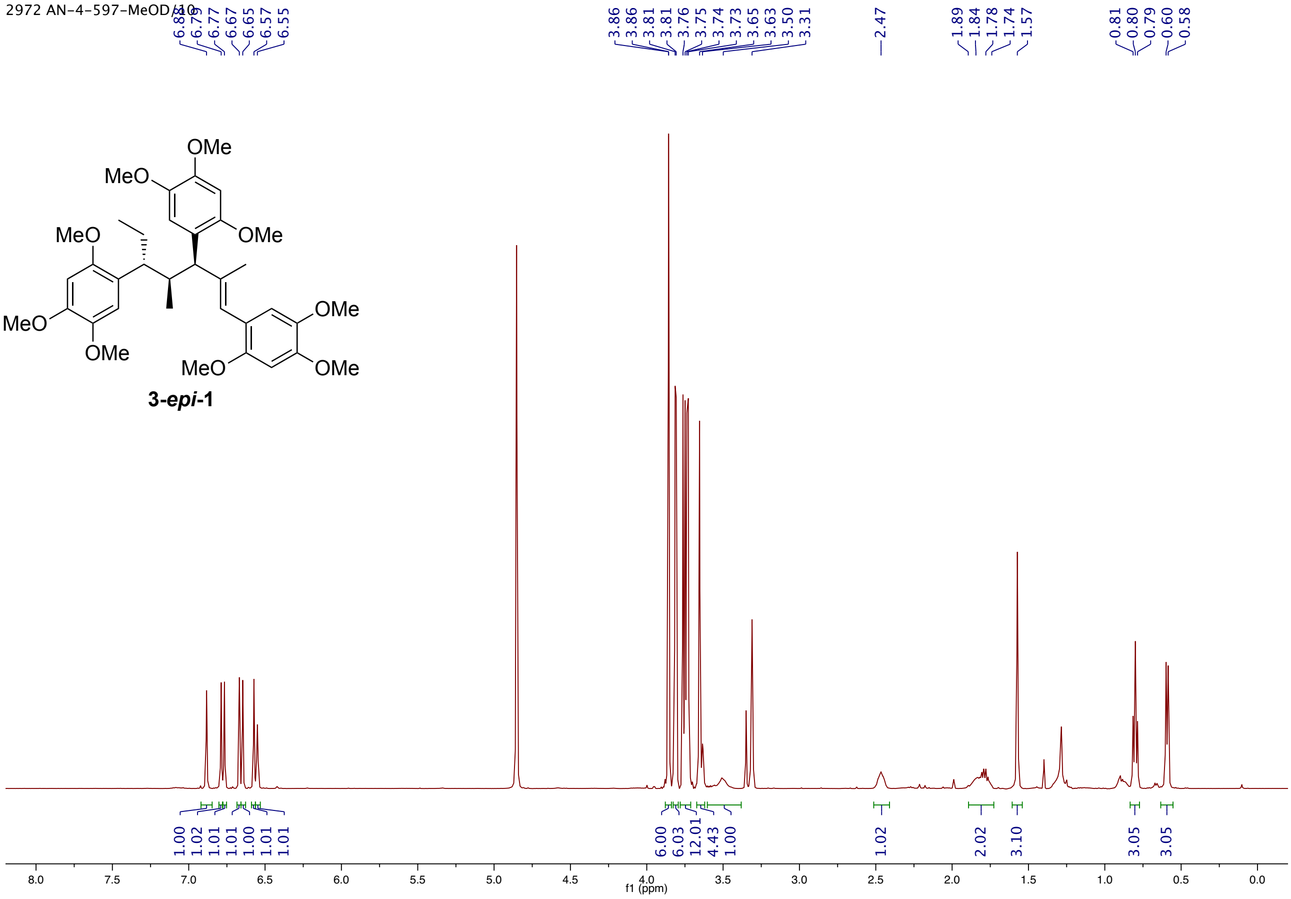

^{13}C NMR (126 MHz, CD_3OD):

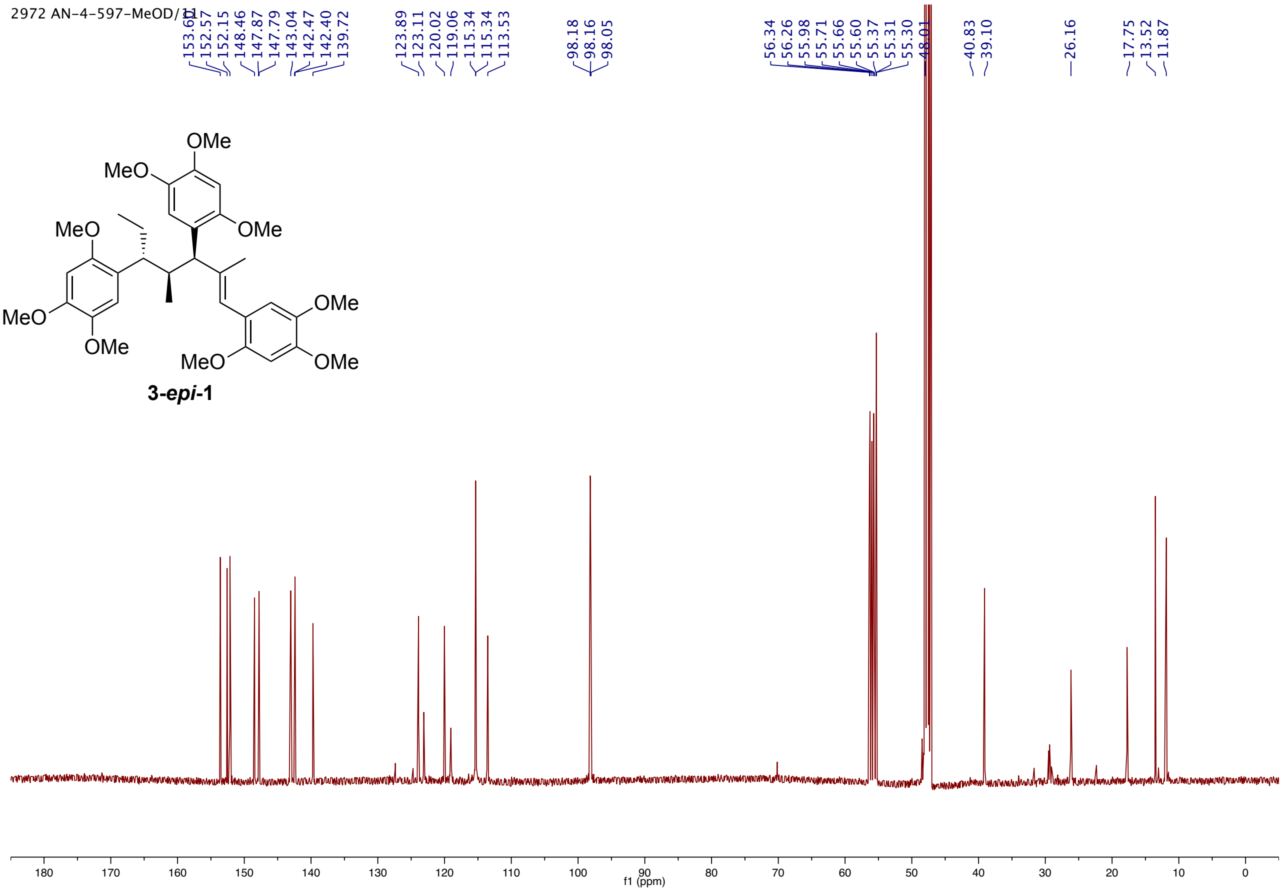

^{1}H NMR (500 MHz, $CDCl_3$):

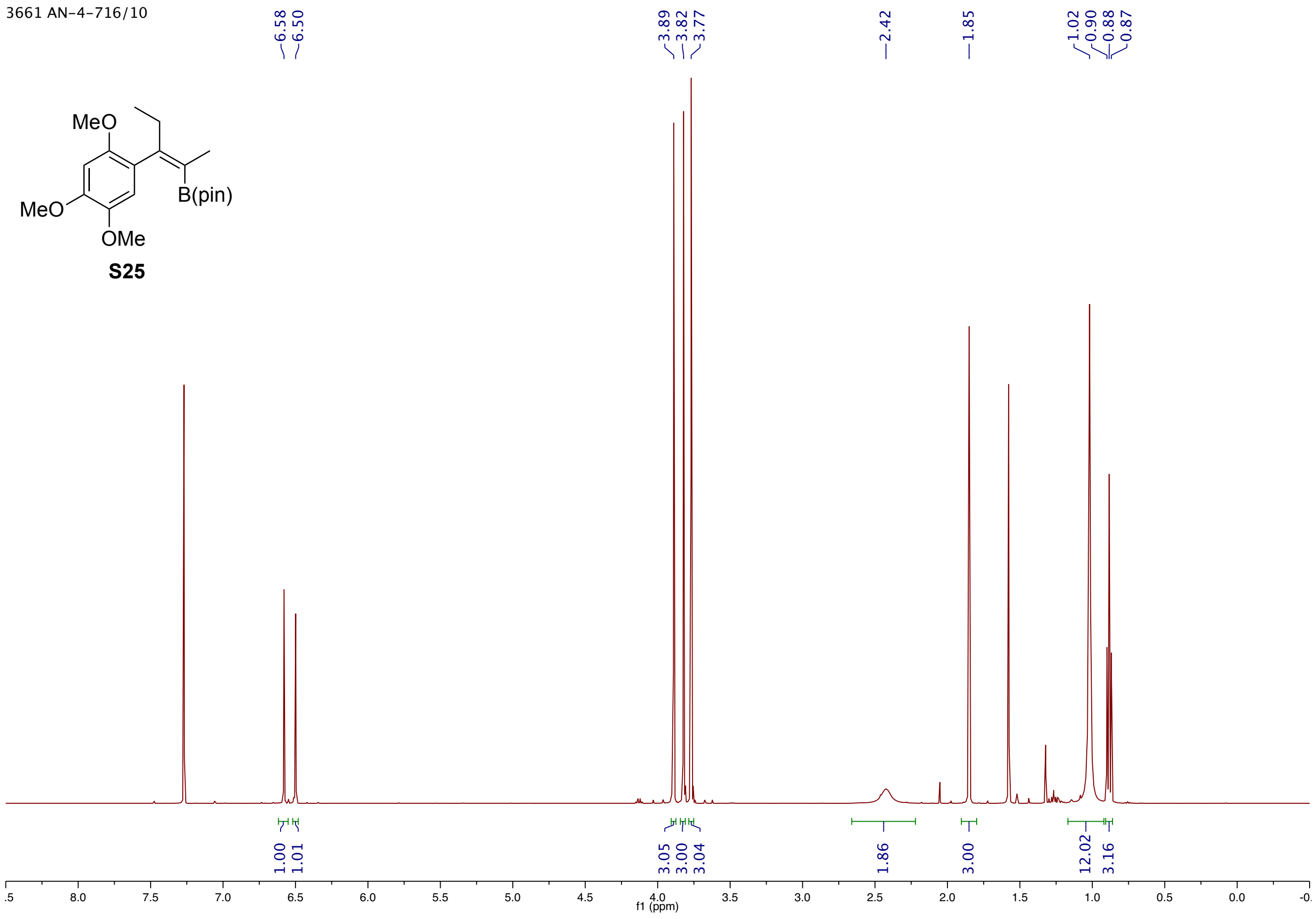

^{13}C NMR (126 MHz, $CDCl_3$):

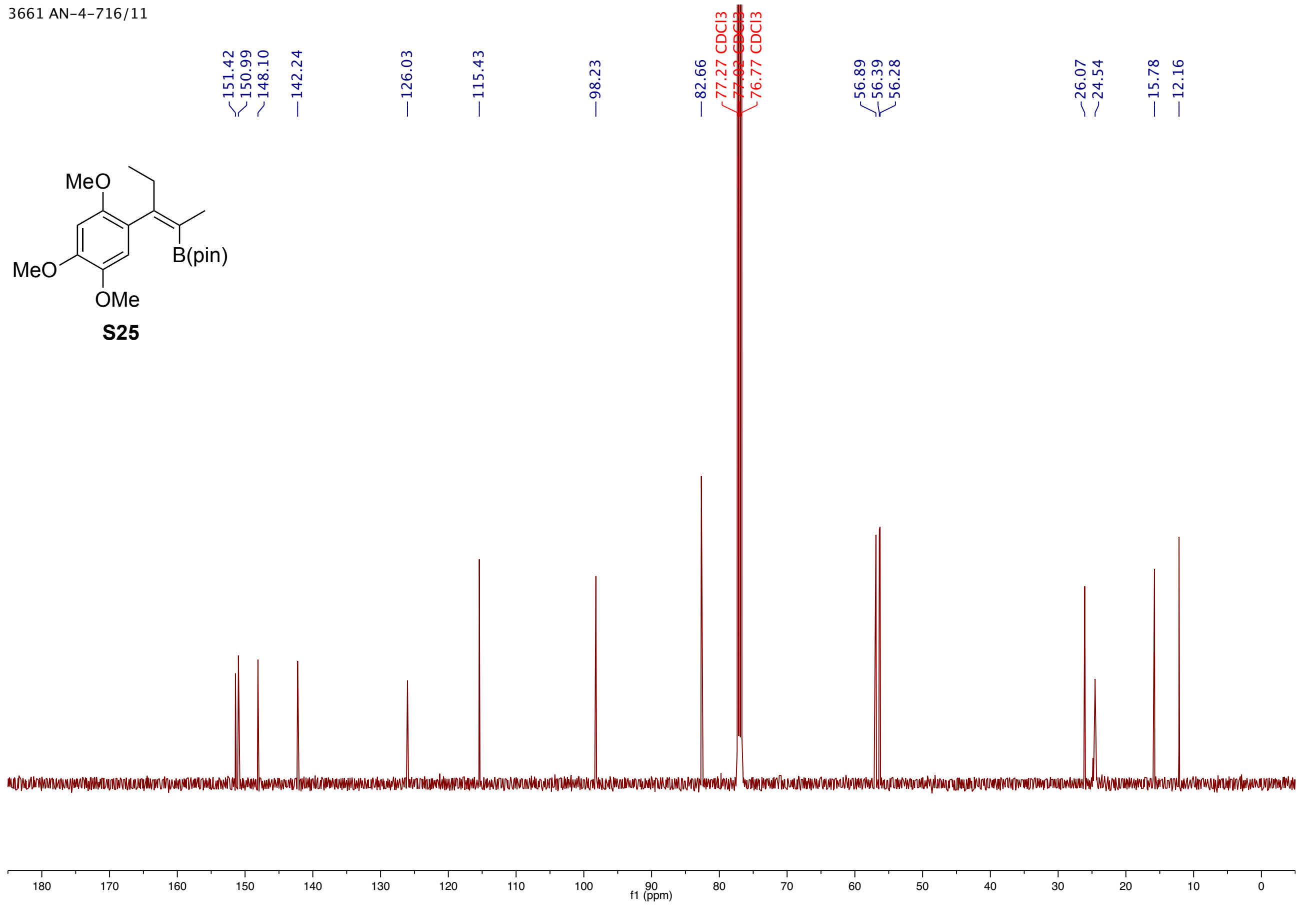

^{1}H NMR (500 MHz, $CDCl_3$):

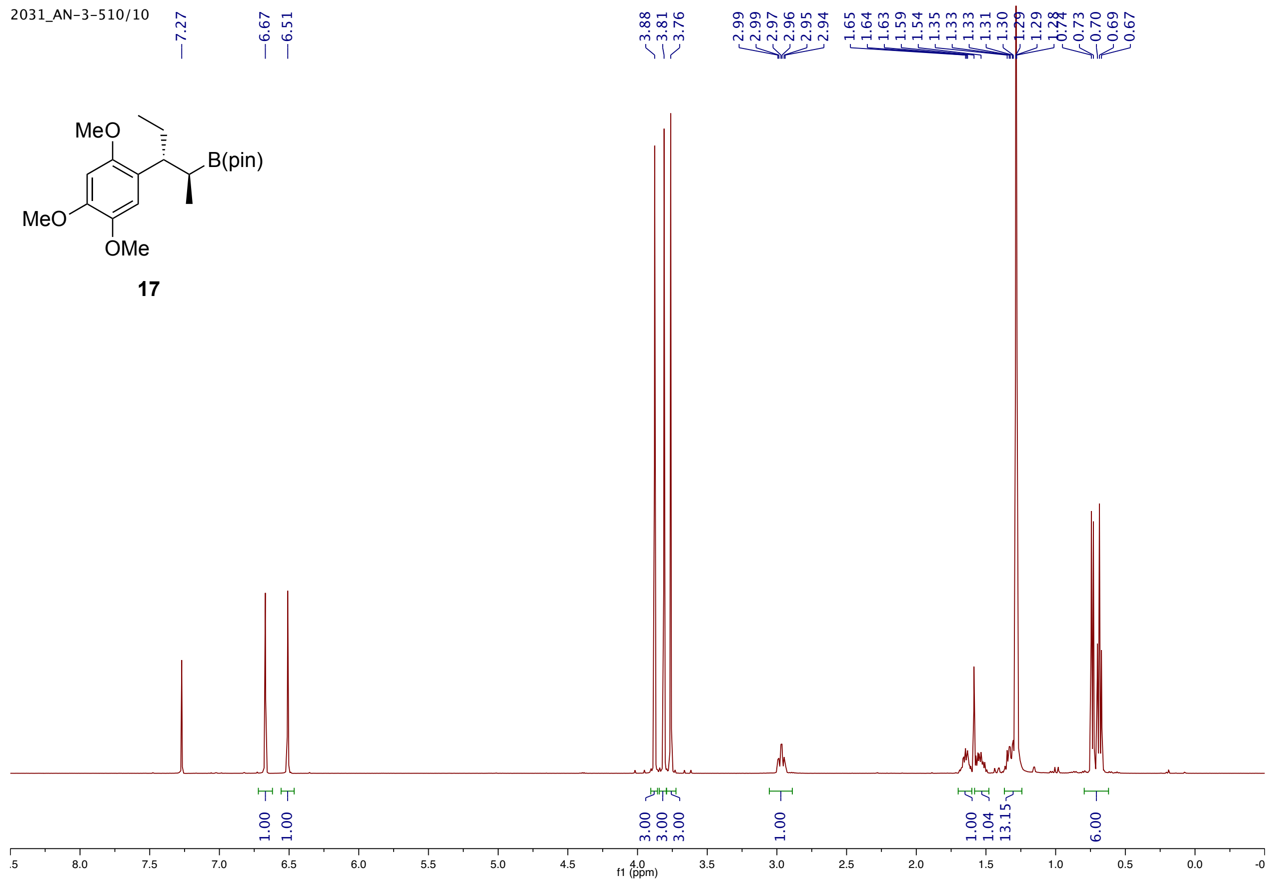

^{13}C NMR (126 MHz, $CDCl_3$):

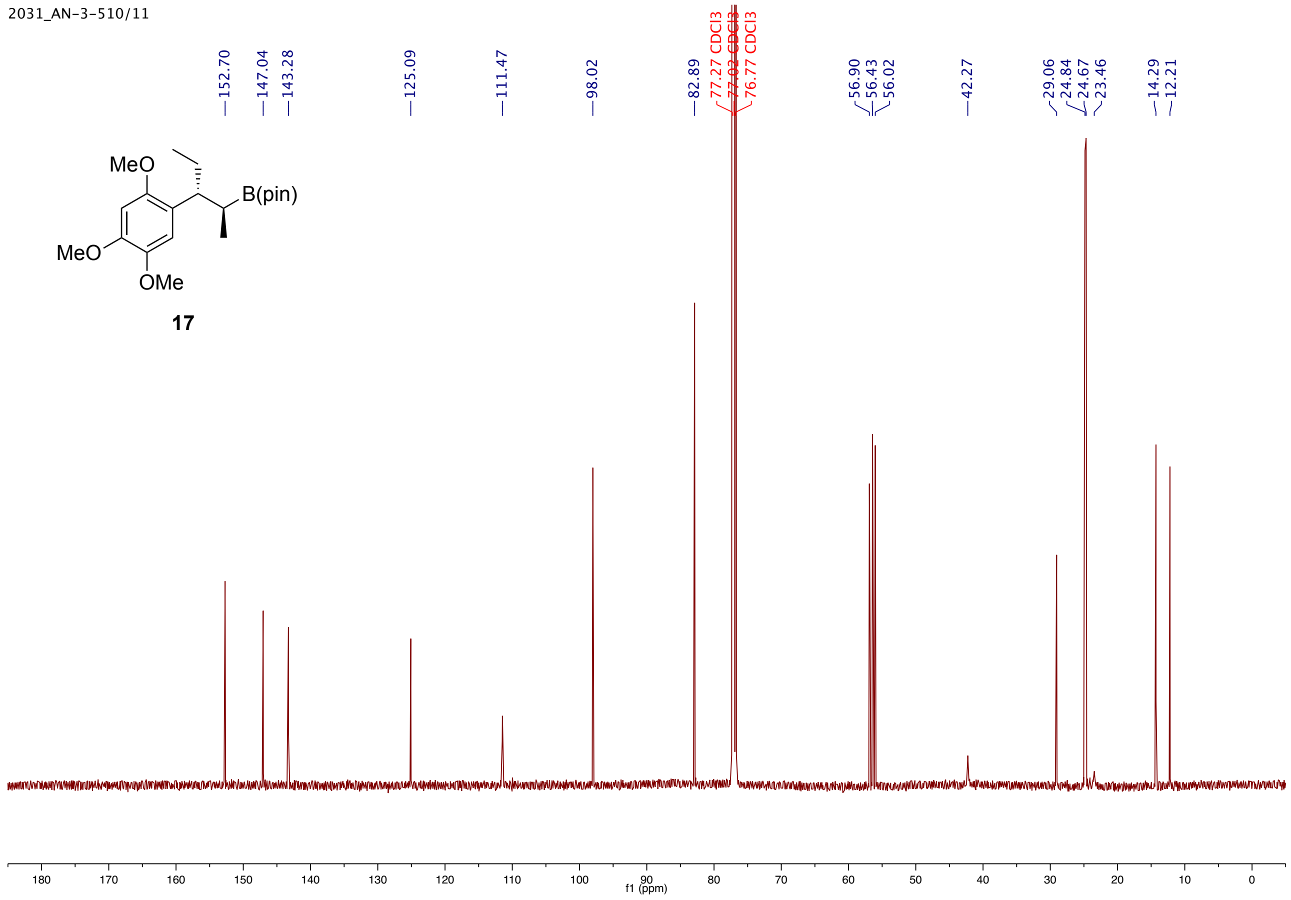

^{1}H NMR (500 MHz, $CDCl_3$):

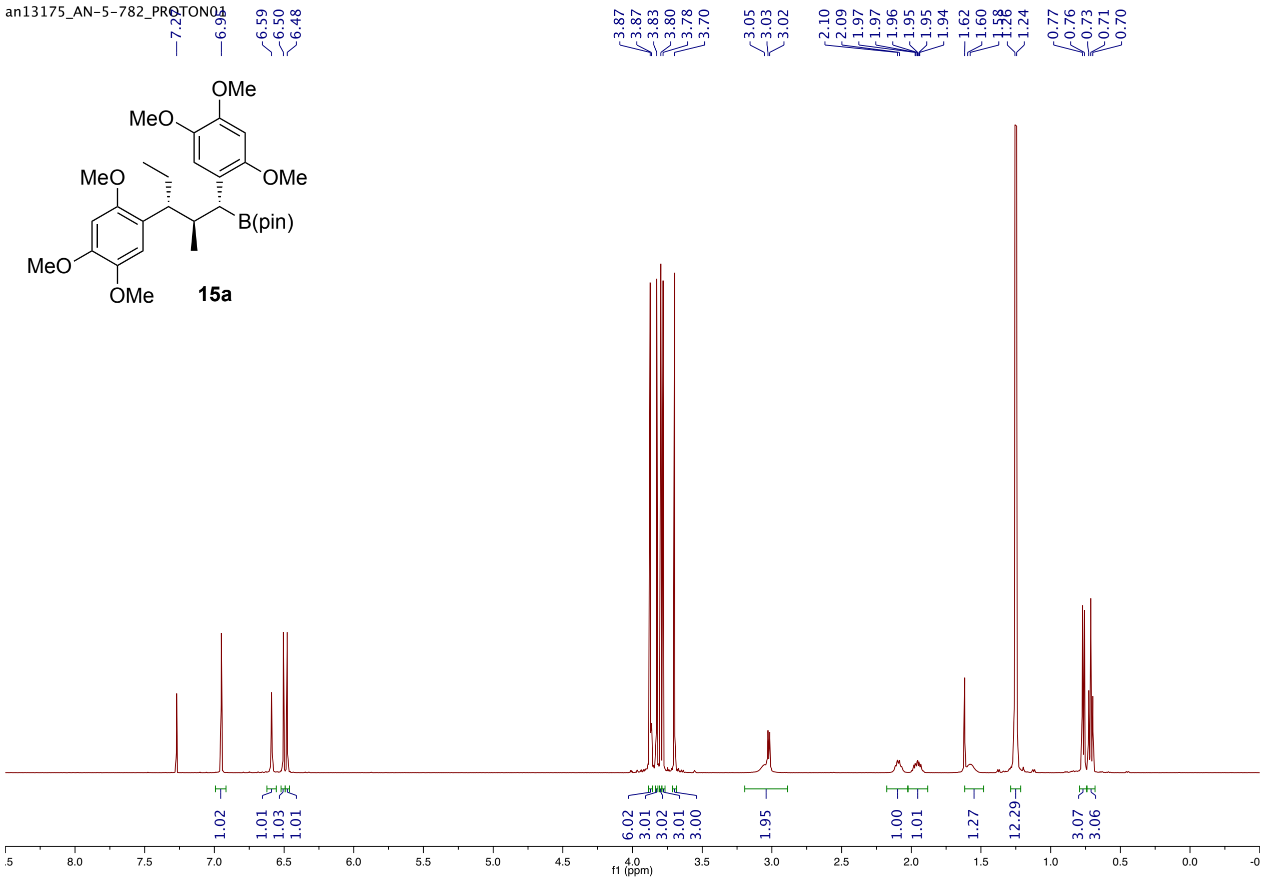

^{13}C NMR (126 MHz, $CDCl_3$):

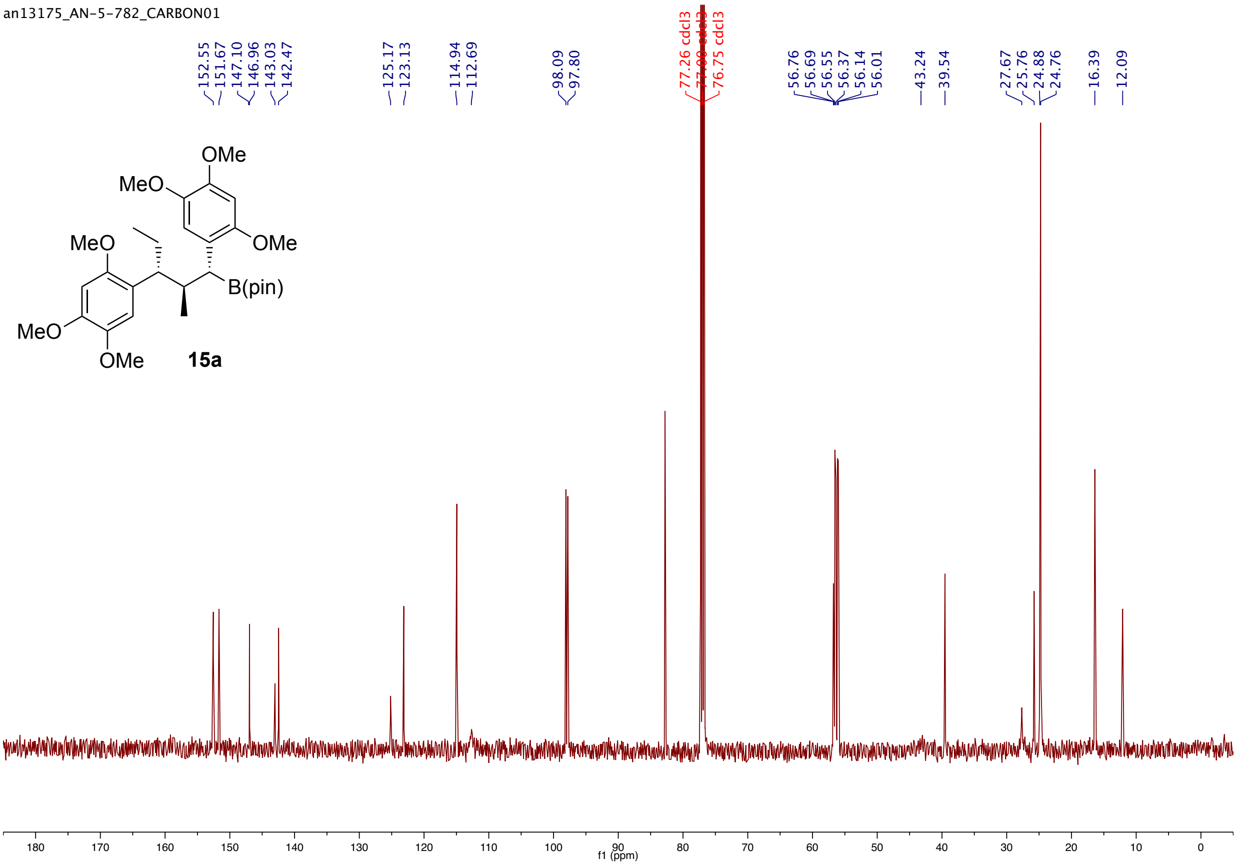

^{1}H NMR (500 MHz, $CDCl_3$):

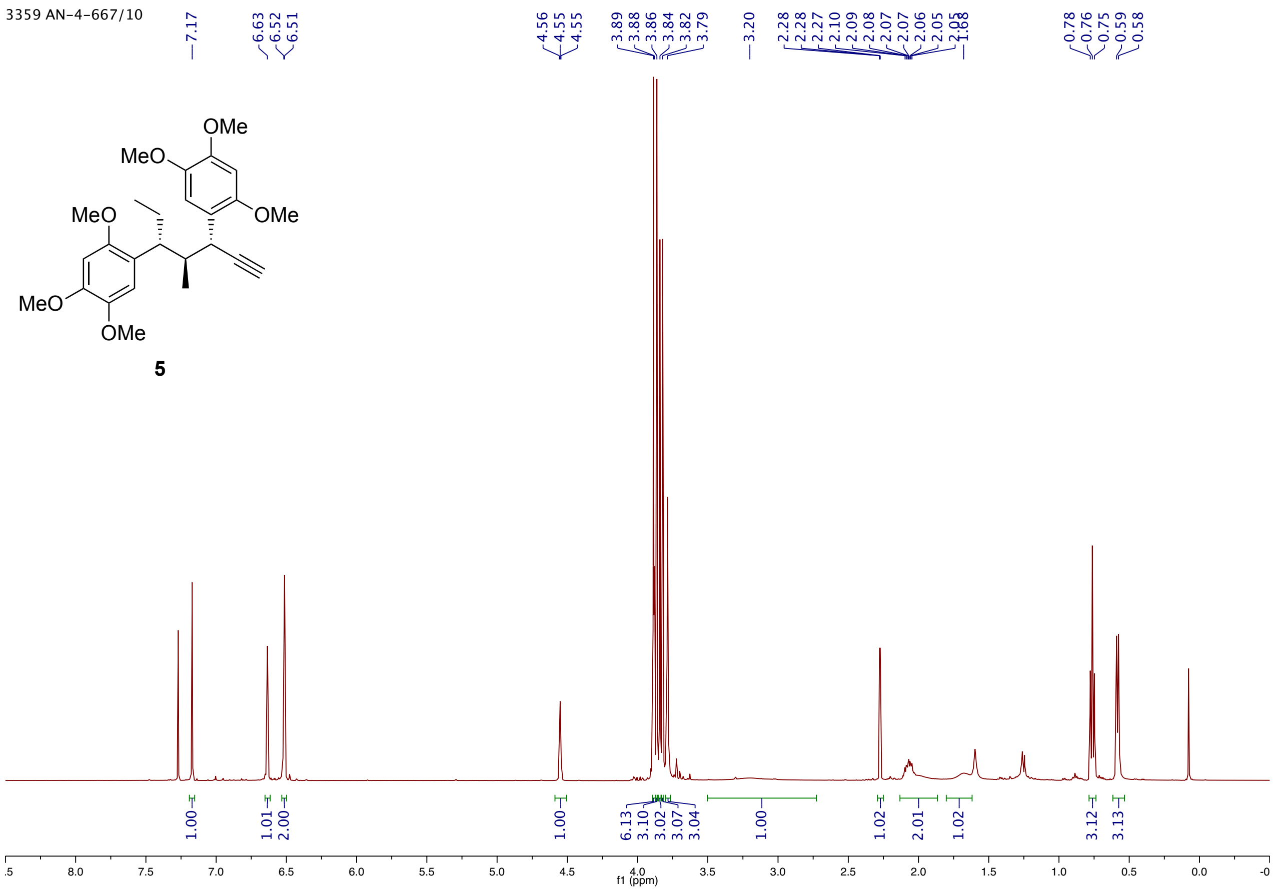

^{13}C NMR (126 MHz, CDCl$_3$):

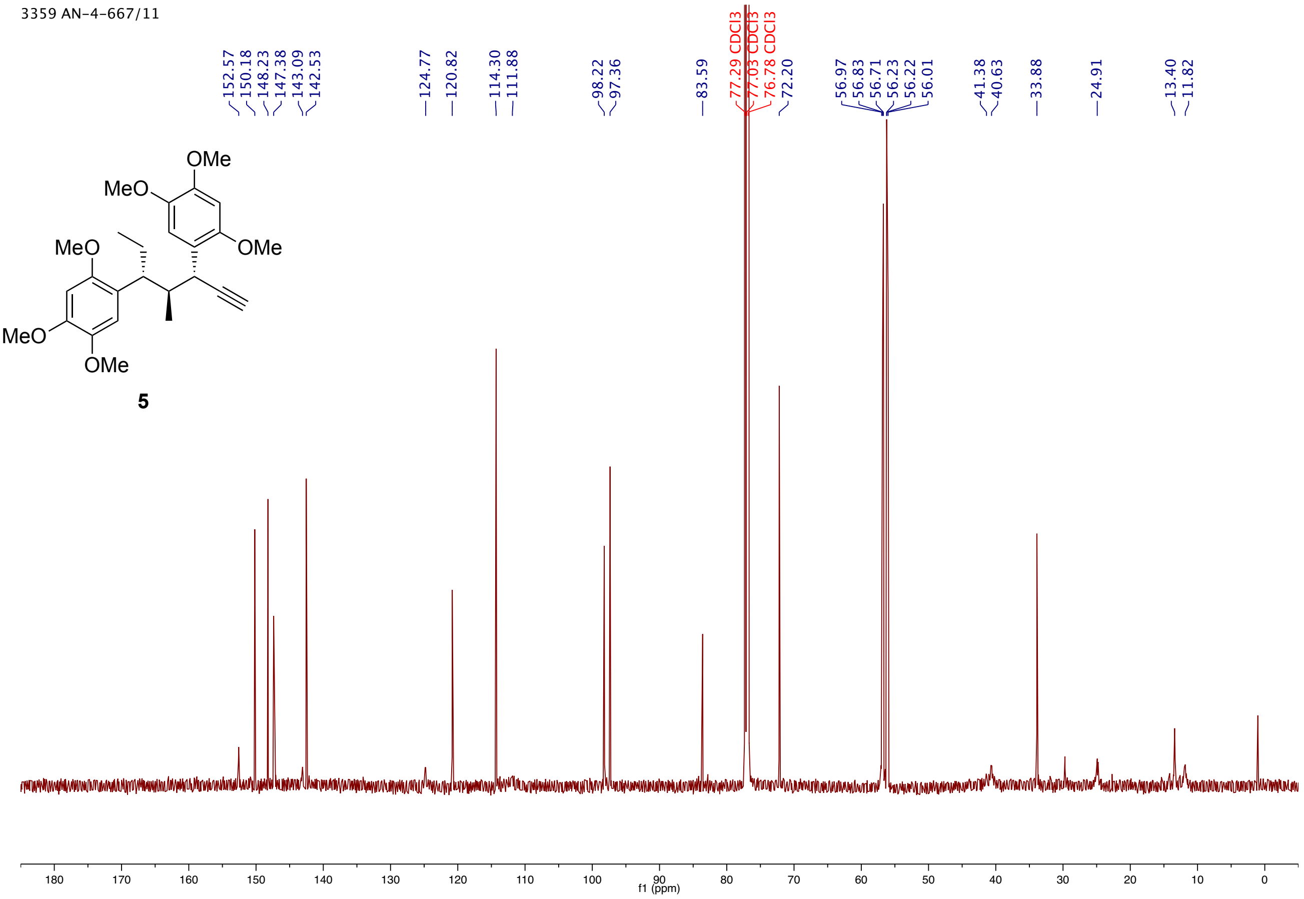

^{1}H NMR (500 MHz, CDCl$_3$):

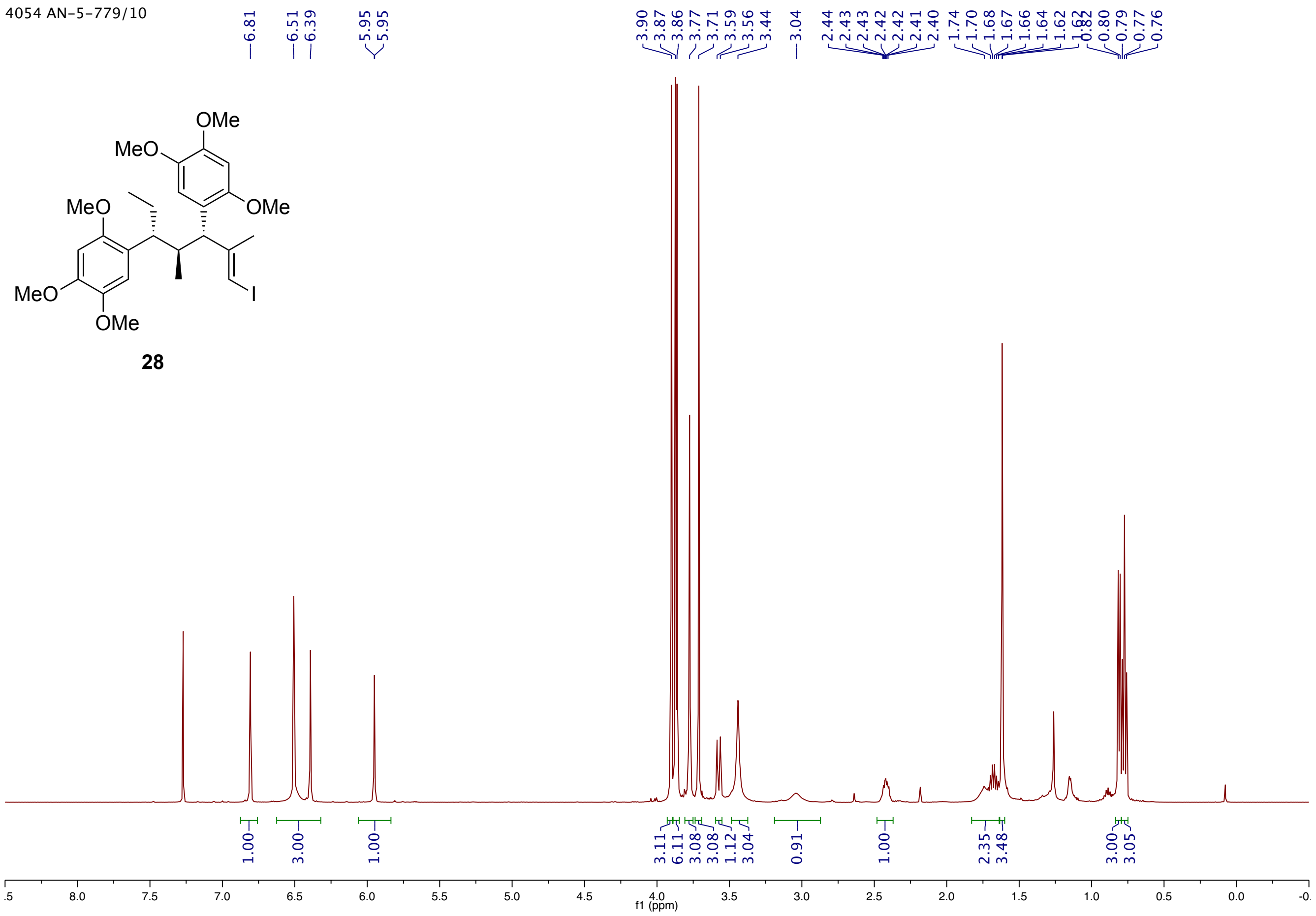

^{13}C NMR (126 MHz, $CDCl_3$):

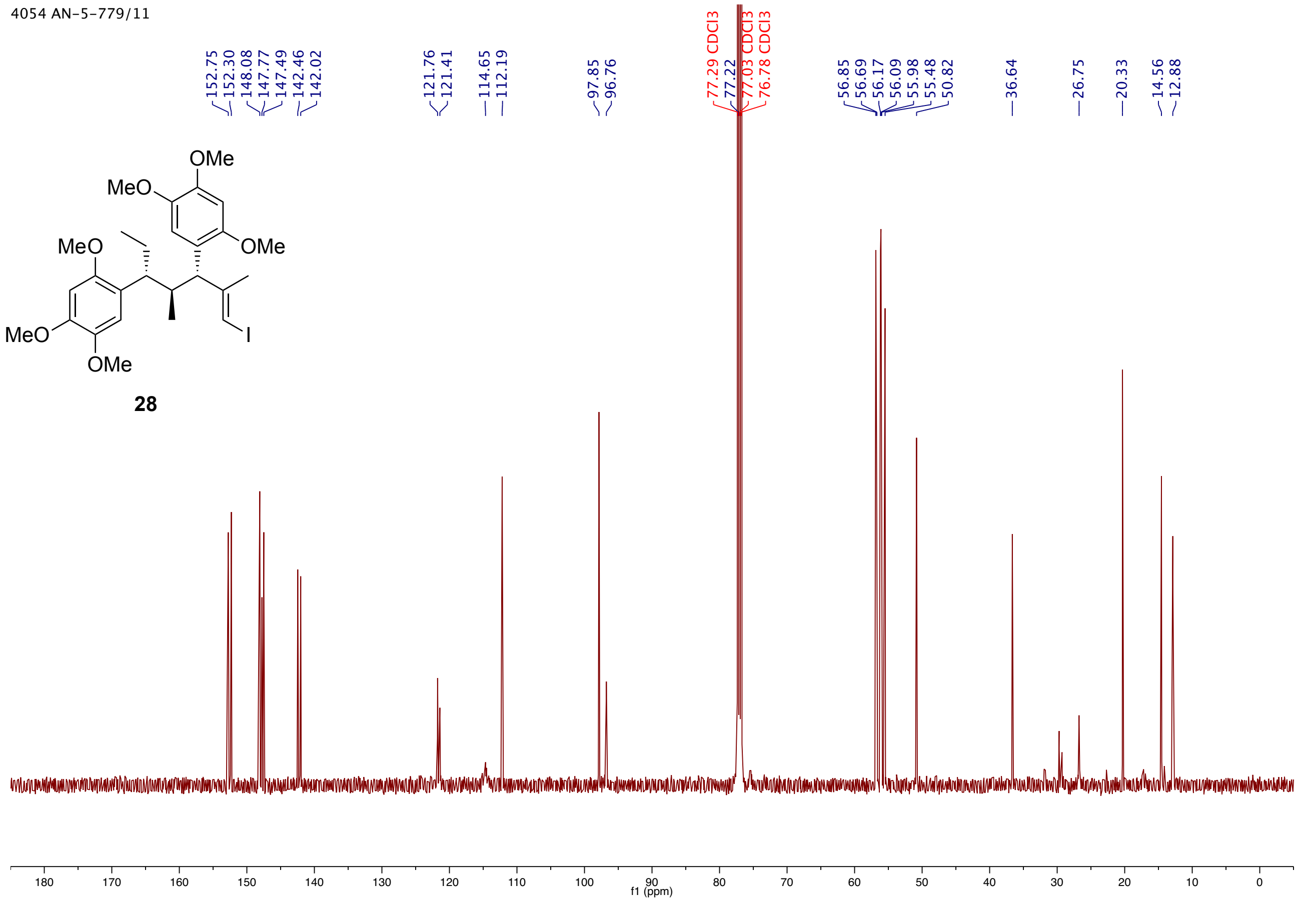

^{1}H NMR (500 MHz, $CDCl_3$):

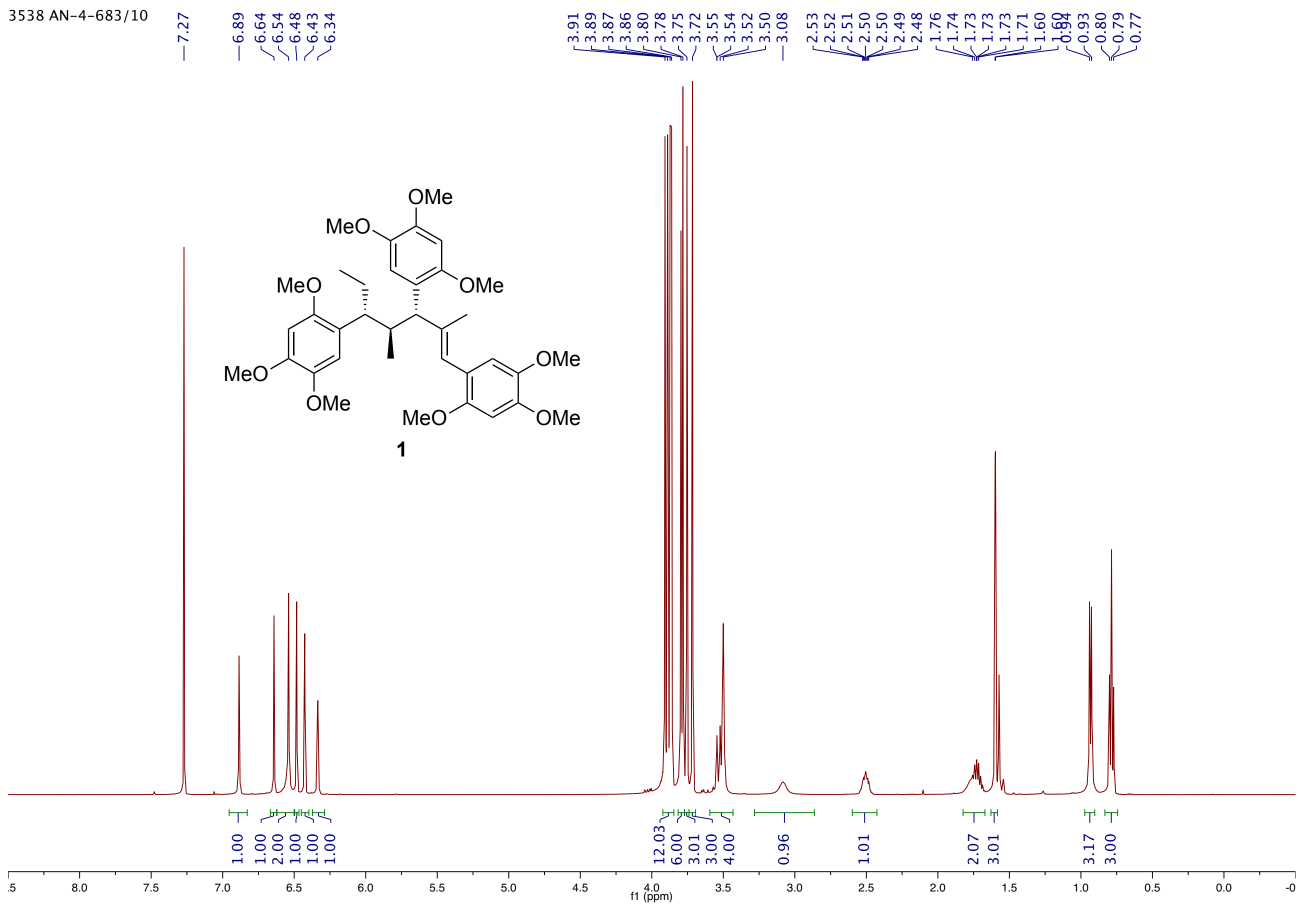

^{13}C NMR (126 MHz, $CDCl_3$):

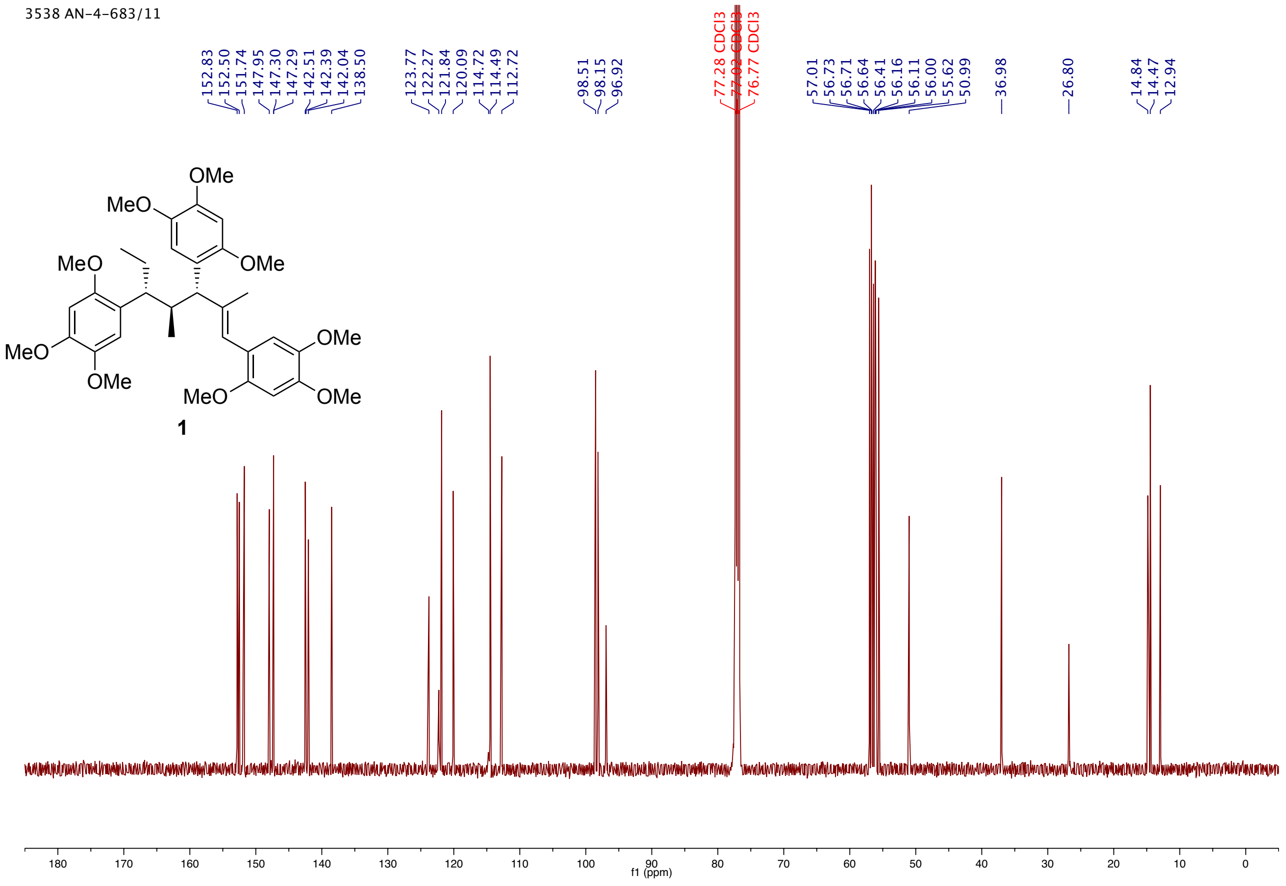

^{1}H NMR (500 MHz, CD_3OD):

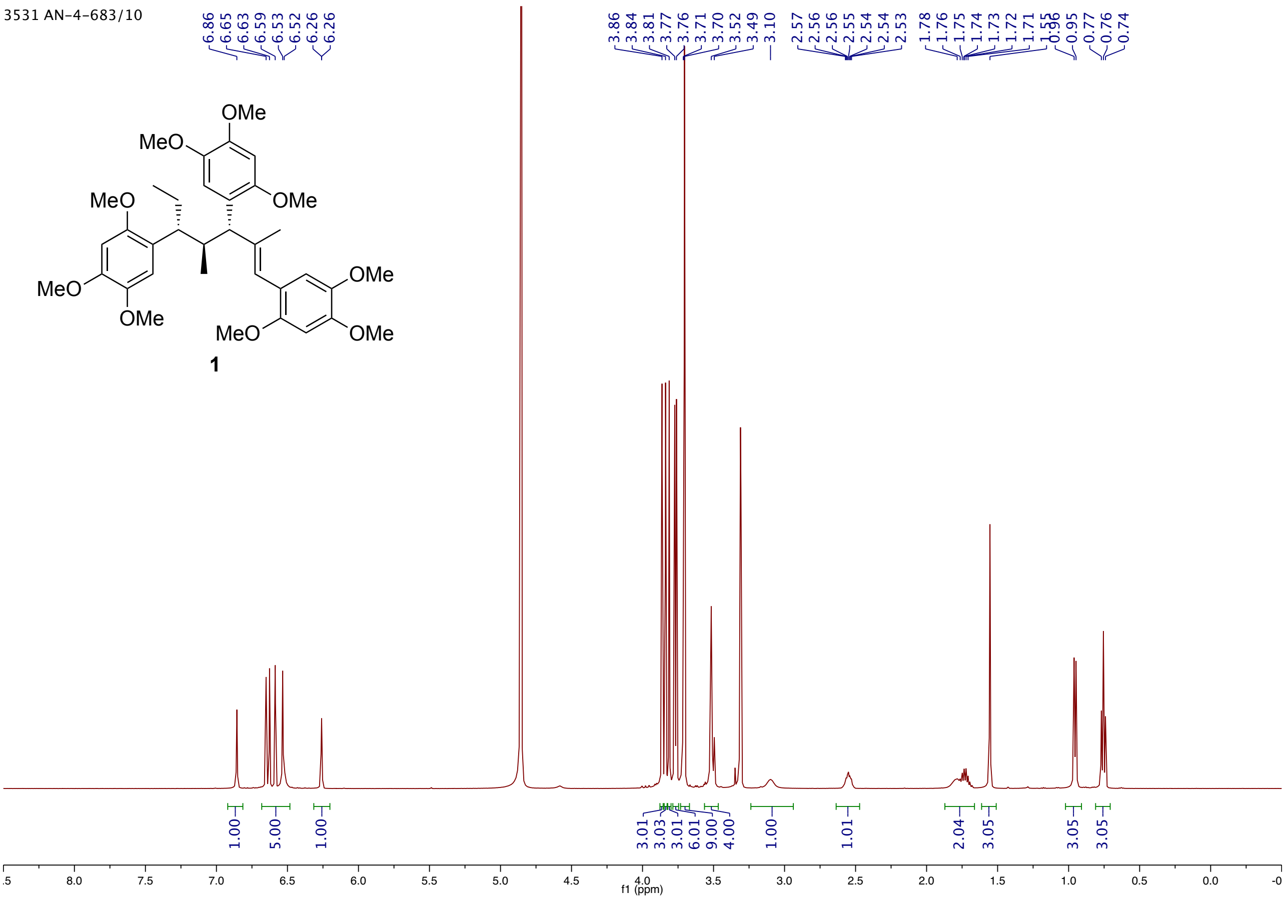

^{13}C NMR (126 MHz, CD_3OD):

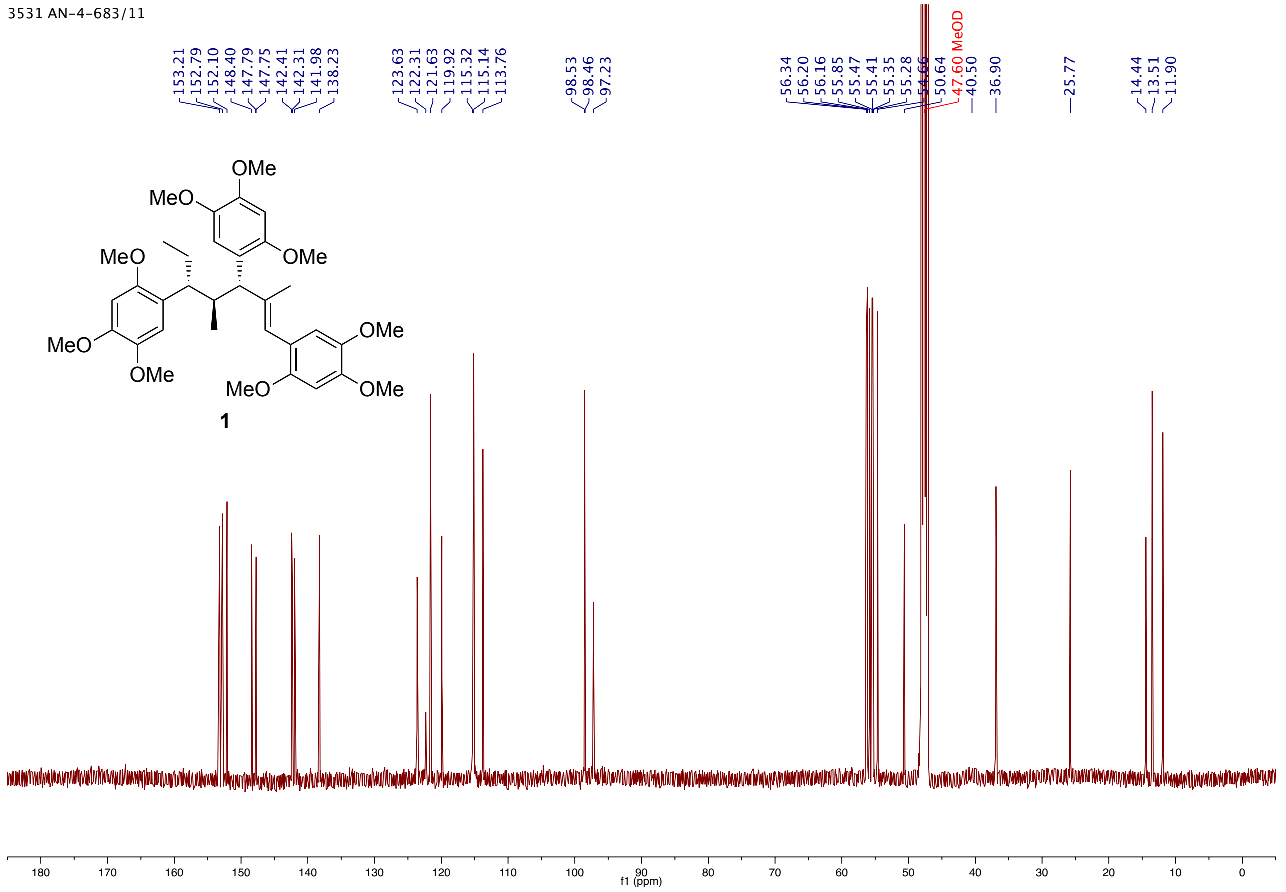

Supplement: Supplementary file 1 — Supplementary [file ANIE-55-15920-s001.pdf]
